# Glycan synthesis with SpyCatcher-SpyTag immobilized Leloir-glycosyltransferases

**DOI:** 10.1007/s00253-025-13602-2

**Published:** 2025-10-24

**Authors:** Philip Palm, Kai Philip Hussnaetter, Lothar Elling

**Affiliations:** Laboratory for Biomaterials, Institute for Biotechnology and Helmholtz-Institute for Biomedical Engineering, RWTH Aachen University, Aachen, Germany

**Keywords:** Leloir glycosyltransferases, Enzymatic glycan synthesis, Immobilization, SpyCatcher, SpyTag, Maleimide-activated agarose

## Abstract

Human milk oligosaccharides (HMOs) have been proven to play key roles in human development and health benefits. Further research requires high-purity HMOs in large quantities. While enzymatic glycan synthesis has been proven to be highly selective and efficient, it is often associated with costly enzyme production and loss of enzymes during synthesis. Immobilization of enzymes can counteract this disadvantage, making synthesis more cost-effective. In this study, we report a versatile immobilization technique for Leloir-glycosyltransferases (GTs) onto maleimide-activated agarose by employing the SpyCatcher-SpyTag technology. The five GTs: human β1,4-galactosyltransferase 1 (β4GalT), *Neisseria meningitidis* β1,3-*N*-acetylglucosaminyltransferase (LgtA), *Escherichia coli* O55:H7 β1,3-galactosyltransferase (WbgO), *Helicobacter pylori* α1,2-fucosyltransferase (FutC), and the human α1,3-*N*-acetylgalactosaminyltransferase GTA/R176G were equipped with SpyCatcher (SpyC), while SpyTag (SpyT) was covalently coupled to the agarose. SpyC-GTs were extensively characterized regarding their preferred buffer system and pH optimum, divalent cation, and substrate kinetics. We successfully immobilized these five SpyC-GTs onto SpyT-agarose, reaching yields between 67 and 100% while retaining their enzymatic activity. Long-term stability over 1 month was enhanced, and reusability for six reactions on three consecutive days was shown. One-pot syntheses employing immobilized SpyC-LgtA, SpyC-WbgO, and SpyC-β4GalT resulted in the primary formation of the tetrasaccharides Lacto-*N*-tetraose (LNT) and Lacto-*N*-neotetraose (LNnT). In sequential syntheses, we were able to produce the HMOs Lacto-*N*-triose II (LNT II), LNT, LNnT, Lacto-*N*-fucopentaose type I (LNFP I), Lacto-*N*-neofucopentaose type I (LNnFP I), and the blood group A antigen hexaoses type I and II (BGA I and II).

**Graphical Abstract:**

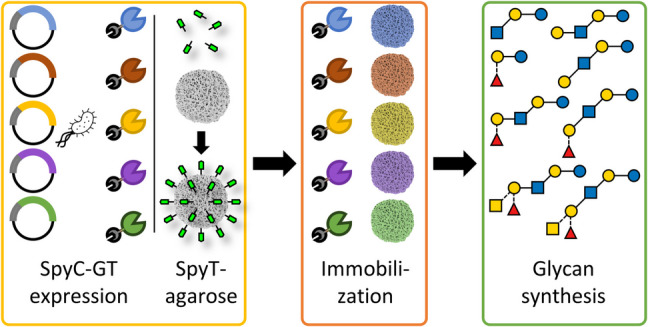

**Supplementary Information:**

The online version contains supplementary material available at 10.1007/s00253-025-13602-2.

## Key points

• *Fusion of Leloir-glycosyltransferases (GTs) with the SpyCatcher (SpyC)*

• *Immobilization of SpyC-GTs on SpyTag agarose*

• *Glycan/HMO synthesis with immobilized SpyC-GTs*

**Supplementary Information:**

The online version contains supplementary material available at 10.1007/s00253-025-13602-2.

## Introduction

Glycans range from simple linear oligosaccharide chains to complex branched structures. They play key roles in cell–cell interactions, attachment of bacteria and virus particles to host cells, cancer metastasis, and general immune response, and are crucial for glycoprotein and -lipid function (Shivatare et al. 2022; Varki 2017). Due to their structural diversity and functional significance, tailored glycan synthesis has become an important aim in biochemical and medical research or biotechnological applications (Hussnaetter et al. 2023). Human milk oligosaccharides (HMOs) are particularly important for health maintenance and development of the immune system in infants (Bode 2012; Chen 2015; Georgi et al. 2013). Furthermore, HMOs show prebiotic effects, inhibit pathogen adhesion, and promote gut barrier integrity. Over 200 distinct HMO structures have been identified, typically composed of lactose with possible extensions of fucose or sialic acid (Kobata 2010; Sprenger et al. 2017; Thurl et al. 2017). This complexity poses challenges to the scalable synthesis of pure HMOs (Petschacher and Nidetzky 2016). While chemical glycan synthesis often applies harsh conditions and lacks natural regio- and stereoselectivity, enzymatic approaches using glycosyltransferases provide high-specificity and mild reaction conditions (Panza et al. 2018).

Leloir-glycosyltransferases (GTs, EC 2.4.) catalyze the transfer of sugar donors from activated nucleotide sugars regio- and stereoselective (Nidetzky et al. 2018). To date, a manifold repertoire of native and engineered GTs is available. For example, the human β1,4-galactosyltransferase 1 (β4GalT) transfers d-galactose in a β1,4 position to *N*-acetylglucosamine (GlcNAc) terminated glycans, forming *N*-acetyllactosamine (Galβ1,4GlcNAc, LacNAc) type II structures and is a key enzyme in complex *N*-glycan synthesis (Sauerzapfe et al. 2008). The β1,3-*N*-acetylglucosaminyltransferase LgtA from *Neisseria meningitidis* adds GlcNAc in a β1,3 position to lactose or LacNAc, enabling the synthesis of lacto-*N*-glycans and HMOs (Hu et al. 2022; Li et al. 2016; Tseng et al. 2024). The β1,3-galactosyltransferases (WbgO) from *Escherichia coli* O55:H7 transfer d-galactose to GlcNAc-terminated glycans in a β1,3 position, facilitating the synthesis of LacNAc (Galβ1,3GlcNAc) type I oligomers (Fischöder et al. 2017; Liu et al. 2009). FutC, an α1,2-fucosyltransferase (α1,2-FucT) from *Helicobacter pylori*, adds l-fucose in an α1,2 position to the terminal galactose residue and is therefore essential in the synthesis of 2′-fucosyllactose (2′-FL), a precursor for AB0 and Lewis blood groups (Drouillard et al. 2006; Fang et al. 2018; Wang et al. 1999). GTA, a human α1,3-*N*-acetylgalactosaminyltransferase, transfers *N*-acetylgalactosamine (GalNAc) to Fucα1,2Galβ-OR, converting blood group H(0) to blood group A epitopes (Seto et al. 1997). Altogether, these GTs provide a versatile toolbox for a great variety of enzymatically synthesized glycans.

Besides costly nucleotide sugars as substrates, the glycosyltransferases themselves are major cost drivers of enzymatic glycan synthesis. Their production and purification are time-consuming processes, and activity may decline during storage. While solubility tags can enhance yields by improving expression (Sauerzapfe et al. 2009, 2008), long-term stability remains challenging. Immobilization of GTs offers a promising solution by improving enzyme stability, simplifying product isolation, and enabling reuse across multiple reactions. Immobilization processes typically involve anchoring glycosyltransferases onto solid-phase materials, such as resins or magnetic beads, allowing substrates to pass through reaction columns (Hussnaetter et al. 2023). Common immobilization strategies include non-covalent methods (e.g., His_6_-tag and Ni^2+^-NTA), covalent approaches (e.g., glutaraldehyde or sortase A crosslinking), as well as encapsulation methods (Ito et al. 2010; Nahalka et al. 2003; Ruzic et al. 2020; Wang et al. 2021). Magnetic beads allow for sequential reactions without cross-contamination between compartments, as shown in human natural killer cell-1 (HNK-1) synthesis (Heinzler et al. 2019). Other approaches employ cationic amphipathic peptides or encapsulation within microenvironments as used for branched HMOs and heparosan production (Naruchi and Nishimura 2011; Qiao et al. 2022). Entrapment in or on microgels enabled the synthesis of the Galili epitope and Lacto-*N*-glycans (Sommerfeld et al. 2024). Enzyme orientation on solid support remains challenging, as wrong alignment can hinder substrate access to active sites. Directed immobilization, using affinity tags or site-specific conjugation, improves enzyme accessibility and catalytic performance (Hussnaetter et al. 2023). A promising immobilization strategy is the SpyCatcher-SpyTag system in which a 113-amino acid SpyCatcher (SpyC, SI Sequence S1) forms an isopeptide bond with a 16-amino acid SpyTag (SpyT, SI Sequence S2), both derived from the CnaB2 domain of the fibronectin-binding protein FbaB from *Streptococcus pyogenes* (Keeble et al. 2019). This stable, biocompatible linkage is ideal for enzyme immobilization.

**Fig. 1:**
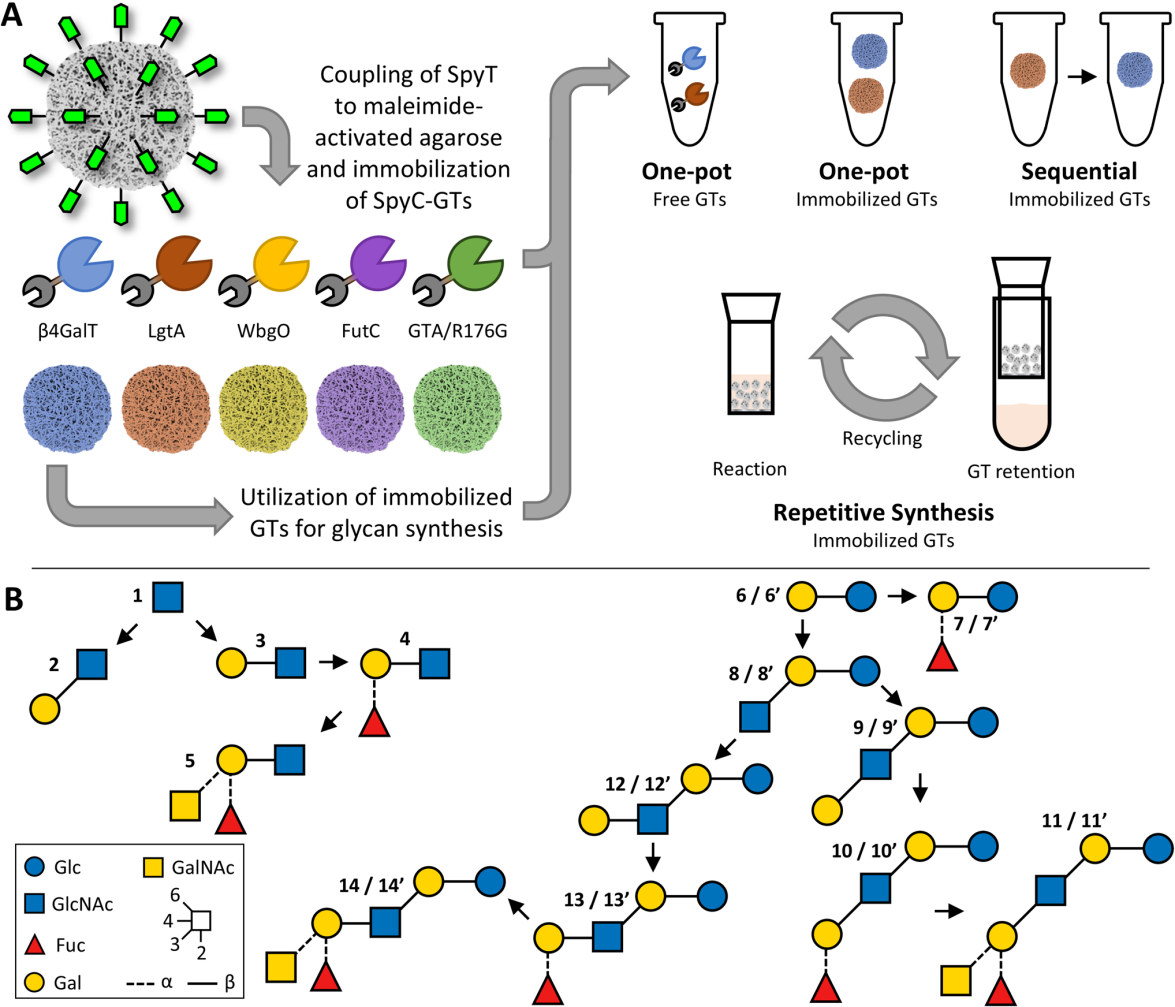
Schematic depiction of the experimental procedure of this work and synthesized/targeted glycans. **A** The Leloir-GTs SpyC-β4GalT, SpyC-LgtA, SpyC-WbgO, SpyC-FutC, and SpyC-GTA/R176G were immobilized onto maleimide-activated agarose via SpyC-SpyT interactions. SpyC-GT agaroses were characterized and utilized for glycan synthesis in one-pot, sequential, and repetitive processes. **B** Depiction of glycans synthesized with immobilized and free SpyC-GTs. The glycans were synthesized with a *t*Boc-linker (non-primed numbers) for HPLC analysis and without an analysis tag (primed numbers) for APTS labeling and analysis. **1**: GlcNAc-*t*Boc; **2**: LacNAc type I-*t*Boc; **3**: LacNAc type II-*t*Boc; **4**: H-antigen type II-*t*Boc; **5**: BGA type II-*t*Boc; starting from Lactosyl-*t*Boc (**6**) or lactose (**6’**); **7**: H-antigen type V-*t*Boc; **7’**: H-antigen type V; **8**: LNT II-*t*Boc; **8’**: LNT II; **9**: LNT-*t*Boc; **9’**: LNT; **10**: LNFP I-*t*Boc; **10’**: LNFP I; **11**: BGA hexaose I-*t*Boc; **11’**: BGA hexaose I; **12**: LNnT-*t*Boc; **12’**: LNnT; **13**: LNnFP I-*t*Boc; **13’**: LNnFP I; **14**: BGA hexaose II-*t*Boc; **14’**: BGA hexaose II (see SI Scheme S1)

In this study (Fig. 1), we evaluated SpyC-SpyT immobilization of five Leloir-GTs: β4GalT, LgtA, WbgO, FutC, and GTA/R176G regarding coupling efficiency, enzymatic activity, long-term stability, and reusability. SpyC was fused N-terminally to each GT, while SpyT was expanded by a GSG-linker and cysteine residue, and coupled site-specific to maleimide-activated agarose via a thiosuccinimide bond (Gunnoo and Madder 2016). The SpyC-GTs were immobilized onto the SpyT-resin. SpyC-β4GalT, SpyC-LgtA, SpyC-WbgO, and SpyC-GTA/R176G were used across six consecutive reactions over 3 days. Maleimide-activated agarose enabled efficient separation of enzymes from reaction mixtures, reducing downstream processing effort. We moreover synthesized several HMOs and Lacto-*N*-glycans (Fig. 1B). These results lay the foundation for (semi-) automated production systems, representing a significant step towards economically viable, cell-free, selective, and scalable enzymatic glycan synthesis.

## Materials and methods

### SpyC-GT plasmid construction

The constructs for the SpyC-GTs were designed for a similar expression plasmid structure, and plasmids were purchased from BioCat (Heidelberg, Germany) with a pETDuet-1 vector as a backbone. The first section of each SpyC-GT is the SpyCatcher003 from *Streptococcus pyogenes* (Keeble et al. 2019) (SI, Scheme S1), which functions as an immobilization tag and is located at the N-terminus of each fusion protein. Connected with a short flexible glycine-serine linker (Keeble and Howarth 2019), a solubility tag follows C-terminally. Here, either the lipase pro-peptide (LPP) from *Staphylococcus hyicus* (Sauerzapfe et al. 2008) or the maltose binding protein (*malE*, MBP) from *Escherichia coli* K-12 (Sauerzapfe et al. 2009) was used. Following another short 4 amino acid linker, the respective GT is located at the C-terminus. With the human β1,4-galactosyltransferase 1 (SpyC-β4GalT, SI Sequence S3) (Sauerzapfe et al. 2008), the β1,3-*N*-acetylglucosaminyltransferase (SpyC-LgtA, SI Sequence S4) from *Neisseria meningitidis* (Hu et al. 2022), the β1,3-galactosyltransferase (SpyC-WbgO, SI Sequence S5) from *E. coli* O55:H7 (Fischöder et al. 2017), the α1,2-fucosyltransferase (SpyC-FutC, SI Sequence S6) from *Helicobacter pylori* (Heine et al. 2022; Wang et al. 1999), and the human α1,3-*N*-acetylgalactosaminyltransferase (SpyC-GTA/R176G, variant according to Seto et al. 1997: R176G, SI Sequence S7) (Seto et al. 1997), five different SpyC-GT constructs were designed for this work (SI, Table S1). The GTA/R176G was demonstrated to strongly increase UDP-GalNAc conversion.

### Production of SpyC-GTs

The plasmids were transformed into different chemically competent *E. coli* expression strains via heat-shock transformation according to the manufacturer’s manual. SpyC-WbgO, SpyC-FutC, and SpyC-GTA/R176G were transformed into BL21 (DE3) (NEB, Ipswich, MA, USA). SpyC-LgtA was transformed into Rosetta 2 (DE3) pLysS (Merck, Darmstadt, Germany), and SpyC-β4GalT was transformed into SHuffle T7 Express (NEB, Ipswich, MA, USA) (SI, Table S1). Heterologous protein expression and the following purification were performed as previously described (Fischöder et al. 2017). In short, transformed *E. coli* were pre-cultivated in a 20 mL LB Liquid culture overnight, transferred to a 1 L TB Liquid culture in a 5 L baffled flask, and cultivated at 37 °C and 80 rpm. Upon reaching an optical density (OD_600_) between 0.5 and 0.8, gene expression was induced by adding 0.1 mM isopropyl-β-d-thiogalactopyranoside (IPTG). Further cultivation and synthesis of recombinant proteins was carried out at 25 °C for 20 to 24 h. Cells were harvested by centrifugation, and the pellet was stored at -20 °C. For purification, 4 to 5 g of cells were diluted in IMAC lysis/binding buffer to a 40% w/v solution and sonicated subsequently. The buffer composition was chosen depending on the stability of the respective GT during purification (SI, Table S1). After centrifugation (26,900 × g, 30 min), the supernatant was filtered (0.8 µm, low protein binding) and applied to a 5 mL HisTrap HP Ni^2+^-NTA column for purification via His_6_-tag on an Äkta system (Cytiva, Marlborough, MA, USA). Bound protein was eluted with the respective lysis/binding buffer containing 500 mM imidazole. A buffer change to the respective storage buffer was carried out by dialysis overnight (SI, Table S2). Protein identity was verified via activity assays, SDS-PAGE, and Immunoblot for detection of the His_6_-tag via anti-His_6_ antibody. SpyC-GTs were stored at 4 °C for several days. For a long-term storage, GTs, solved in respective storage buffer, were lyophilized (-60 °C, 0.003 mPa, 20 h), stored at -80 °C, and resuspended in MQ-H_2_O for usage.

### SpyC-GT activity assays

Assessment of enzymatic activity was carried out on a microliter scale using lyophilized SpyC-GTs and either GlcNAc-*t*Boc (compound **1**, Fig. 1B) (Sauerzapfe et al. 2009), Lactosyl-*t*Boc (Lac-*t*Boc, **6**) (Slamova et al. 2023), or lactose-based glycans as acceptor substrates, together with the respective nucleotide sugars as donor substrates (see SI for details). The lyophilized SpyC-GTs were freshly diluted in MQ water and added to the respective reaction buffer (SI, Table S2; acceptor substrates: SpyC-β4GalT: GlcNAc-*t*Boc; SpyC-LgtA: Lac-*t*Boc (**6**, Fig. 1B) or lactose (**6’**); SpyC-WbgO: GlcNAc-*t*Boc (**1**); SpyC-FutC: Lac-*t*Boc (**6**) or lactose (**6’**); SpyC-GTA/R176G: 2’-FL-*t*Boc (**7**) or 2’-FL (**7’**)), and incubated for up to 24 h at 30 °C. At given time points, samples were taken, denatured at 95 °C for 5 min, centrifuged (12,050 × g, 5 min), and the supernatant was analyzed via high-performance liquid chromatography (HPLC, see SI) for *t*Boc-linked sugars or via capillary electrophoresis with LED-induced fluorescence detection (CE-LIF, see SI) for lactose-based glycans. Enzyme kinetics were performed as described in the supplementary information (see SI Table S3 for reaction buffers).

### Immobilization of SpyC-GTs on maleimide-activated agarose via SpyC-SpyT interaction

Maleimide-activated agarose was purchased from CubeBiotech (Monheim, Germany). The SpyT that was used for coupling to the agarose was derived from SpyTag003 (Keeble et al. 2019), elongated N-terminally by a short GS-spacer and an additional cysteine (underlined) (peptide sequence: CGSGRGVPHIVMVDAYKRYK), and purchased from BioCat (Heidelberg, Germany). Maleimide-activated agarose was equilibrated in SpyT coupling buffer (100 mM sodium acetate, 150 mM NaCl, pH 6.5) according to the manufacturer, and SpyT was added with a concentration of 5 mg·mL^−1^ agarose with a subsequent incubation at room temperature overnight in an end-over-end shaker. The coupling reaction was stopped by several washing and centrifugation steps (1 mL SpyT coupling buffer, 12,050 × *g*, 2 min) while the supernatant was gathered and pooled for the determination of excess protein via Bradford assay. Subsequently, the SpyT-agarose was equilibrated in the respective SpyC-GT coupling buffer (which equals the storage buffer, see SI Table S2) for the immobilization of SpyC-GTs. Lyophilized SpyC-GTs were diluted to a concentration of 2 mg∙mL^−1^, and 1.5 mg of enzyme was added to 1 mL SpyT-agarose. The coupling reaction was incubated at 4 °C overnight in an end-over-end shaker and stopped by several washing and centrifugation steps. Coupling yield was determined via Bradford assay, and the enzymatic activity of immobilized SpyC-GTs was ascertained by an activity assay (SI, Table S2). SpyC-GTs immobilized to SpyT-agarose were stored in the respective coupling buffer at 4 °C (SI, Table S2).

### Syntheses of Lacto-N-glycans

#### One-pot syntheses

The *t*Boc-linked LNT (**9**, Fig. 1B) and LNnT (**12**) were synthesized with SpyT-agarose immobilized SpyC-GTs in a one-pot reaction and with free SpyC-GTs as a comparative approach. For LNT (**9**) synthesis, SpyC-GT-agaroses or free SpyC-GTs, respectively, were homogenously mixed in a 1:5 unit ratio (2 mU SpyC-WbgO, 10 mU SpyC-LgtA). The reaction mixture, containing 100 mM HEPES pH 7.5, 25 mM KCl, 5 mM MnCl_2_, 5 mM MgCl_2_, 5 mM Lac-*t*Boc (**6**), 6.5 mM UDP-Gal, 6.5 mM UDP-GlcNAc, and 5 U FastAP (Thermo Fisher, Waltham, MA, USA), was added to the enzymes to start the reaction. For LNnT (**12**) synthesis, SpyC-GT-agaroses or free SpyC-GTs, respectively, were homogenously mixed in a 2:1 unit ratio (13 mU SpyC-β4GalT, 7 mU SpyC-LgtA). The reaction mixture, containing 100 mM glycine, pH 10, 5 mM MnCl_2_, 5 mM Lac-*t*Boc (**6**), 6.5 mM UDP-Gal, 6.5 mM UDP-GlcNAc, and 5 U FastAP, was also added to the enzymes to start the reaction. Reactions were incubated at 30 °C for 24 h with intermediate sampling and a subsequent HPLC analysis (see SI).

#### Sequential syntheses by a combination of immobilized and free GTs

Lactose-based glycans were synthesized with immobilized SpyC-GTs and lactose (**6’**, Fig. 1B) as the initial acceptor substrate in a sequential procedure. The LNT II (**8’**) reaction mixture contained 100 mM glycine, pH 10, 5 mM MnCl_2_, 5 mM lactose, 6.5 mM UDP-GlcNAc, 3 U FastAP, and 105 µg SpyC-LgtA (100 µL SpyC-LgtA agarose) in a final volume of 200 µL. For LNT (**9’**) synthesis, 100 mM HEPES pH 7.5, 25 mM KCl, 5 mM MgCl_2_, 6.5 mM UDP-Gal, 3 U FastAP, and 112 µg SpyC-WbgO (100 µL SpyC-WbgO agarose) were added to the supernatant of the previous reaction. For LNnT (**12’**) synthesis, 100 mM glycine, pH 10, 5 mM MnCl_2_, 6.5 mM UDP-Gal, 3 U FastAP, and 112 µg SpyC-β4GalT (100 µL SpyC-β4GalT agarose) were added to the supernatant of the previous reaction. The fucosylated Lacto-*N*-glycans LNFP I (**10’**) and LNnFP I (**13’**) were synthesized in a subsequent fucosylation step of LNT (**9’**) and LNnT (**12’**) with a non-SpyC FutC (His_6_-LPP-FutC). Five microliters of the LNT or LNnT reaction, respectively, were added to the reaction mixture containing 100 mM TRIS pH 7.5, 25 mM KCl, 5 mM MgCl_2_, 5 mM MnCl_2_, 6.5 mM GDP-Fuc, 3 U FastAP, and 100 µg His_6_-LPP-FutC in a total volume of 100 µL. Conversion of LNFP I (**10’**) and LNnFP I (**13’**) to BGA hexaose type I (**11’**) and BGA hexaose type II (**14’**) by SpyC-GTA/R176G was targeted in a reaction mixture containing 100 mM MOPS pH 7, 20 mM MgCl_2_, 6.5 mM UDP-GalNAc, 3 U FastAP, 1 mg·mL^−1^ bovine serum albumin (BSA), and 75 µg SpyC-GTA/R176G (37.5 µL SpyC-GTA/R176G agarose) with 80 µL of the previous approaches in a total volume of 200 µL. Incubation of all reactions was done for 24 h at 30 °C with intermediate sampling. The reactions were halted by enzyme denaturation at 95 °C for 5 min, followed by centrifugation (12,050 × g, 5 min). Product formation was verified by CE-LIF analysis (see SI).

For HPLC–ESI–MS analytics (see SI), *t*Boc-based hexaoses BGA I (**11**, Fig. 1B) and II (**14**) were also synthesized with SpyC-GTs and in a sequential procedure. Here, the method used is the same as for the lactose-based sequential synthesis, except 5 mM Lac-*t*Boc was used as the initial acceptor substrate, and free SpyC-GTs (100 µg each; 75 mg SpyC-GTA/R176G) were used. Again, LNFP I and LNnFP I were acquired by fucosylation of LNT and LNnT by His_6_-LPP-FutC.

## Results

### Production and activity of SpyC-GTs

All five SpyC-GTs were successfully produced in the respective *E. coli* strains. Besides verification of the expression via SDS-PAGE and Western blot (SI, Fig. S1-S10), their catalytic activity toward their substrates was demonstrated (SI, Fig. S11-S15). Also, the activity of lyophilized enzymes was confirmed (SI, Table S4). We could prove that all five SpyC-GTs retained their activity over lyophilization, and with that, the enzymes can be stored for several months.

### Characterization of SpyC-GTs

SpyC-β4GalT, SpyC-LgtA, SpyC-FutC, and SpyC-GTA/R176G were individually screened for their optimal buffer system, pH value, and co-factor concentration. Moreover, substrate kinetics were evaluated. For SpyC-WbgO, the reaction parameters for the LPP-WbgO fusion protein were used (Fischöder et al. 2017).

#### pH screening of SpyC-GTs

**Fig. 2:**
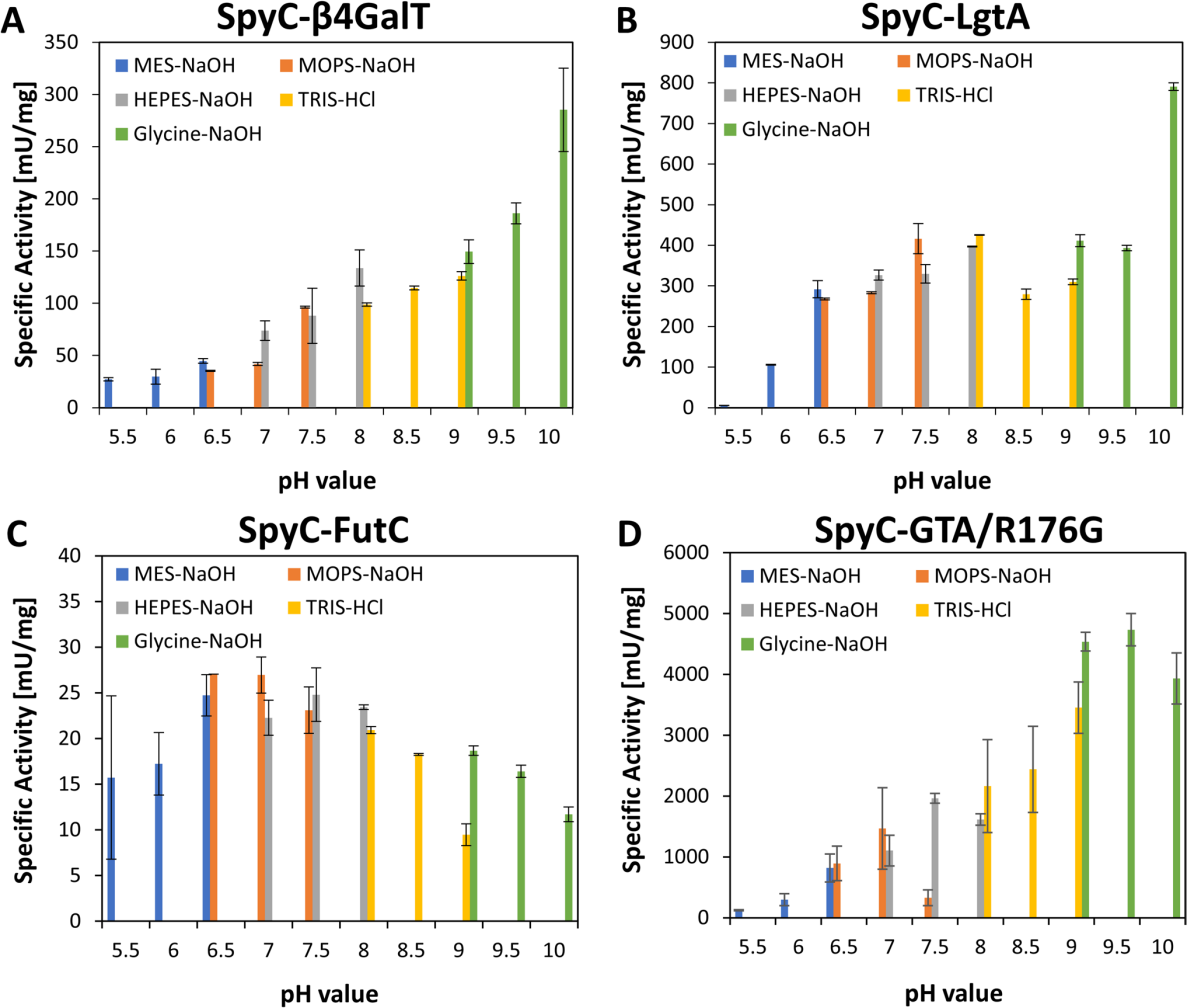
Screening of the optimal buffer system and pH value for SpyC-GTs. Specific activity [mU∙mg^−1^] in the dependency of buffer component and pH value is shown (SI, Table S5). Highest enzymatic activity of: **A** SpyC-β4GalT: 285 mU∙mg^−1^ in glycine-NaOH pH 10; **B** SpyC-LgtA: 791 mU∙mg^−1^ in glycine-NaOH pH 10; **C** SpyC-FutC: 27 mU∙mg^−1^ in MOPS-NaOH pH 6.5; **D** SpyC-GTA/R176G: 4734 mU∙mg.^−1^ in glycine-NaOH pH 9.5 (SI, Table S6)

Five different buffer systems spanning a pH range from 5.5 to 10 were evaluated (SI, Table S5). SpyC-β4GalT and SpyC-LgtA show their highest activity at pH 10 in a glycine–NaOH buffer (Fig. 2A and B and SI, Table S6). SpyC-FutC maintains relatively high shares of its maximum activity throughout the whole measured pH range, with its highest activity in MOPS at pH 6.5-7 (Fig. 2C). For SpyC-GTA/R176G, the highest activity is measured at pH 9.5 in glycine-NaOH (Fig. 2D and SI, Table S6).

#### Co-factor screening of SpyC-GTs

**Fig. 3:**
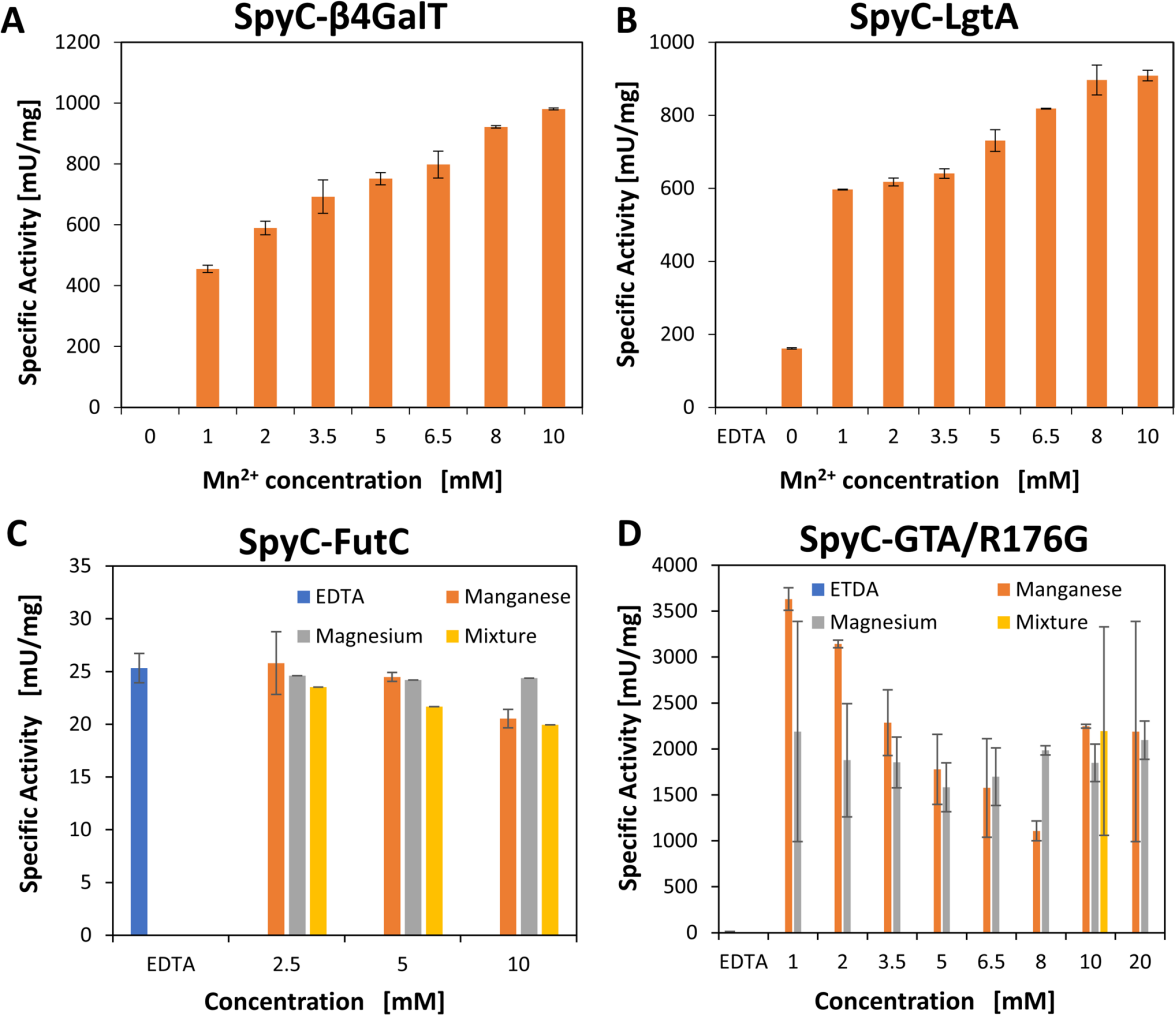
Screening of the optimal cofactor concentration for Spy-GTs. Specific activity [mU∙mg^−1^] in the presence of different divalent cation concentrations and EDTA is shown (see SI, Table S7). Highest activity for: **A** SpyC-β4GalT: 10 mM Mn^2+^; **B** SpyC-LgtA: 10 mM Mn^2+^; **C** SpyC-FutC: no clear preference can be observed; **D** SpyC-GTA/R176G: 1 mM Mn^2+^

SpyC-GTs were screened for their activity with Mn^2+^ and/or Mg^2+^ as cofactors. Moreover, an evaluation of general dependency on metal ions was conducted by depleting soluble metal ions with EDTA (SI, Table S7). SpyC-β4GalT needs Mn^2+^ and shows increasing enzymatic activity with an increasing Mn^2+^ concentration in a range from 1 to 10 mM (Fig. 3A). The screening of SpyC-LgtA with Mn^2+^ revealed a similar behavior, but we could see residual activity when no divalent cation was present (Fig. 3B). SpyC-FutC exhibited equal activity in the presence of Mn^2+^ and Mg^2+^, as well as with EDTA in the reaction mix (Fig. 3C), showing that a co-factor is not needed, but divalent cations are tolerated. The screening for SpyC-GTA/R176G shows a clear dependency on divalent metal ions and a preference for Mn^2+^ over Mg^2+^. Low cofactor concentrations result in a higher catalytic activity (Fig. 3D).

#### Kinetic characterization

The kinetics of SpyC-GTs were evaluated concerning their donor and acceptor substrates (Table 1). For SpyC-β4GalT, the UDP-Gal substrate kinetic approaches saturation with high concentrations. The modest slope is also represented by the *K*_*M*_ value of 9.79 mM. For the acceptor substrate GlcNAc-*t*Boc (**1**, Fig. 1), a substrate excess inhibition was observed (SI, Fig. S16), with an optimal substrate concentration of 2.34 mM. The early saturation of the donor substrate curve of SpyC-LgtA is represented by the *K*_*M*_ value of 0.89 mM, and a substrate excess inhibition was seen for the acceptor Lac-*t*Boc (**6**) with an optimal substrate concentration of 6.5 mM (SI, Fig. S17). For SpyC-FutC, the GDP-Fuc substrate kinetics show a high initial slope and saturation of enzyme activity at GDP-Fuc concentrations higher than 0.5 mM, which is reflected by a low *K*_*M*_ of 0.52 mM. For the acceptor substrate LacNAc-*t*Boc (**3**), a decrease in activity was observed at 25 mM compared to 20 mM, indicating saturation or the onset of a product inhibition (SI, Fig. S18). SpyC-GTA/R176G shows similar *K*_*M*_ values for both donor (UDP-GalNAc) and acceptor (2′-FL-*t*Boc, **7**) substrates with 2.26 and 2.28 mM, respectively. Substrate excess inhibition was observed for the acceptor substrate 2′-FL-*t*Boc (**7**) with a *K*_*i*_ of 6.77 mM (SI, Fig. S19).

**Table 1:** Substrate kinetics of the SpyC-GTs. Michaelis-Menten constant (*K*_*M*_), inhibition constant (*K*_*i*_), turnover number (*k*_cat_), catalytic efficiency (*k*_cat_∙*K*_*M*_^−1^), optimal substrate concentration ([*S*]_opt_)

**Enzyme**	**Substrate**^**a**^	***K***_***M***_ [mM]	***k***_**cat**_ [s^−1^]	***k***_**cat**_**/*K***_***M***_ [mM^−1^∙s^−1^]	***K***_***i***_ [mM]	**[*S*]**_**opt**_ [mM]
SpyC-β4GalT	UDP-Gal	9.79	1.66	0.17	-	-
	GlcNAc-*t*Boc (**1**)	2.34	5.86	2.5	2.34	2.34
SpyC-LgtA	UDP-GlcNAc	0.89	9.34	10.52	-	-
	Lac-*t*Boc (**6**)	6.5	30.71	4.72	6.5	6.5
SpyC-FutC	GDP-Fuc	0.52	0.06	0.12	-	-
	LacNAc-*t*Boc (**3**)	15.66	0.29	0.02	-	-
SpyC-GTA/R176G	UDP-GalNAc	2.26	62.36	27.59	-	-
	2′-FL-*t*Boc (**7**)	2.28	93.36	41.01	6.77	3.93

^a^ Numbers in parentheses depict the compounds in Fig. 1B

### Immobilization of SpyC-GTs on SpyT-agarose

**Fig. 4:**
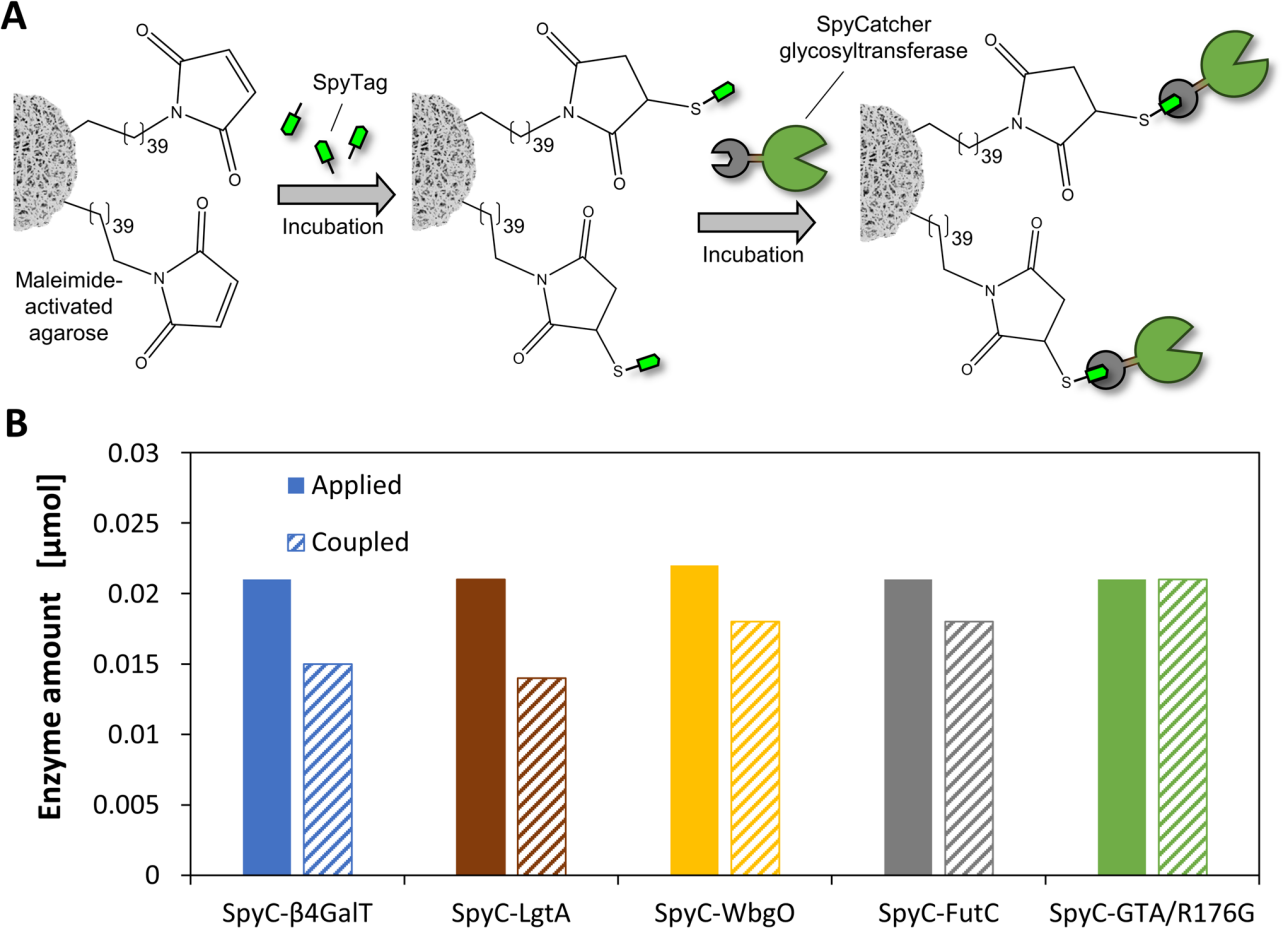
Coupling reaction of SpyT and SpyC-GTs on maleimide-activated agarose and evaluation of coupling efficiency. **A** Coupling reactions of SpyT and SpyC were carried out overnight in an end-over-end shaker. SpyT was coupled at room temperature and a pH of 6.5 in the first incubation step (see SI, Table S8), while subsequently the respective SpyC-GT was immobilized at 4 °C in the corresponding coupling buffer. **B** Coupling efficiencies of the immobilized SpyC-GTs (see SI, Table S9)

Immobilization of SpyC-GTs was performed in two steps, where first SpyT was coupled to the maleimide-activated agarose, followed by the immobilization of SpyC-GTs (Fig. 4A). In nearly all approaches, 5 mg SpyT could be coupled to 1 mL maleimide-activated agarose, resulting in an overall coupling efficiency of more than 99% (SI, Table S8). All GTs were successfully coupled to the SpyT-agarose (coupling yields: SpyC-LgtA 67%, SpyC-β4GalT 71%, SpyC-WbgO 82%, SpyC-FutC 86%, SpyC-GTA/R176G 100%, Fig. 4B and SI, Table S9). Comparative activity assays of the immobilized and the soluble SpyC-GTs showed that all SpyC-GTs remained active after coupling, although a loss in activity was measured. With 49%, SpyC-WbgO retained the highest amount of activity, while SpyC-LgtA retained the least with 20% (SI, Table S10).

### Long-term stability of SpyT-agarose immobilized SpyC-GTs

**Fig. 5:**
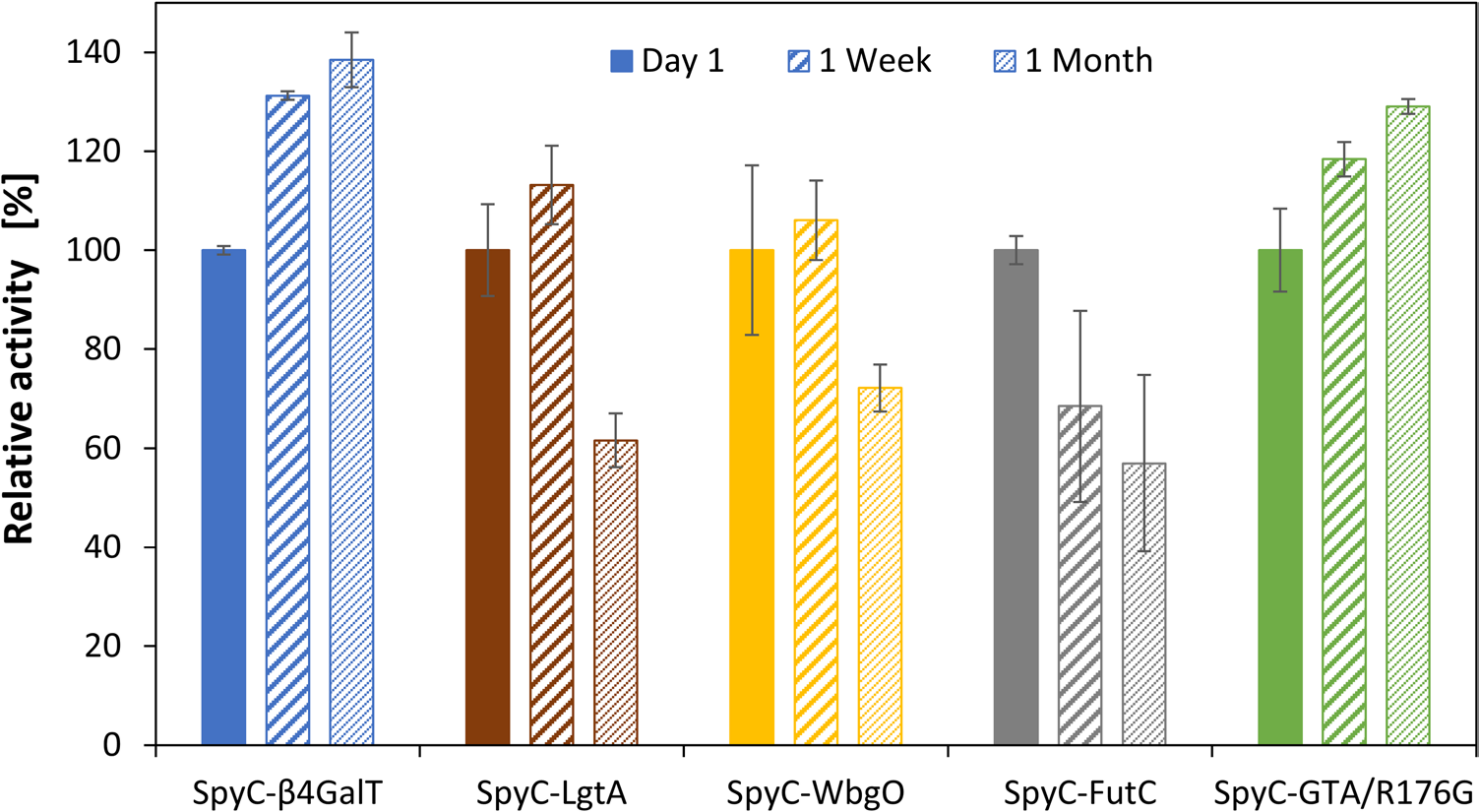
Relative activities of immobilized SpyC-GTs. Specific activities were normalized to the initial activity (100%) on day one. SpyC-β4GalT: 1 week 131%, 1 month 138%; SpyC-LgtA: 1 week 113%, 1 month 62%; SpyC-WbgO: 1 week 106%, 1 month 72%; SpyC-FutC: 1 week 68%, 1 month 57%; SpyC-GTA/R176G: 1 week 118%, 1 month 129% (SI, Table S11)

All SpyC-GTs remained active over the observed time (Fig. 5 and SI, Fig. S20 and Table S11). After 1 week, in comparison to the initial activities, SpyC-β4GalT, SpyC-LgtA, SpyC-WbgO, and SpyC-GTA/R176G showed an increase of 31%, 13%, 6%, and 18%, respectively, while SpyC-FutC activity decreased by 31%. After 1 month, SpyC-LgtA, SpyC-WbgO, and SpyC-FutC showed decreased activities, with the highest loss of 57% for SpyC-FutC. Comparison with the free enzymes reveals stabilization of SpyC-β4GalT, SpyC-LgtA, SpyC-WbgO, and SpyC-FutC by immobilization on SpyT-agarose. With less than 5% of the initial activity left for the free SpyC-WbgO and SpyC-FutC, immobilization has the highest stabilizing effect for these GTs (SI, Fig. S20).

### Reusability of SpyT-agarose immobilized SpyC-GTs

**Fig. 6:**
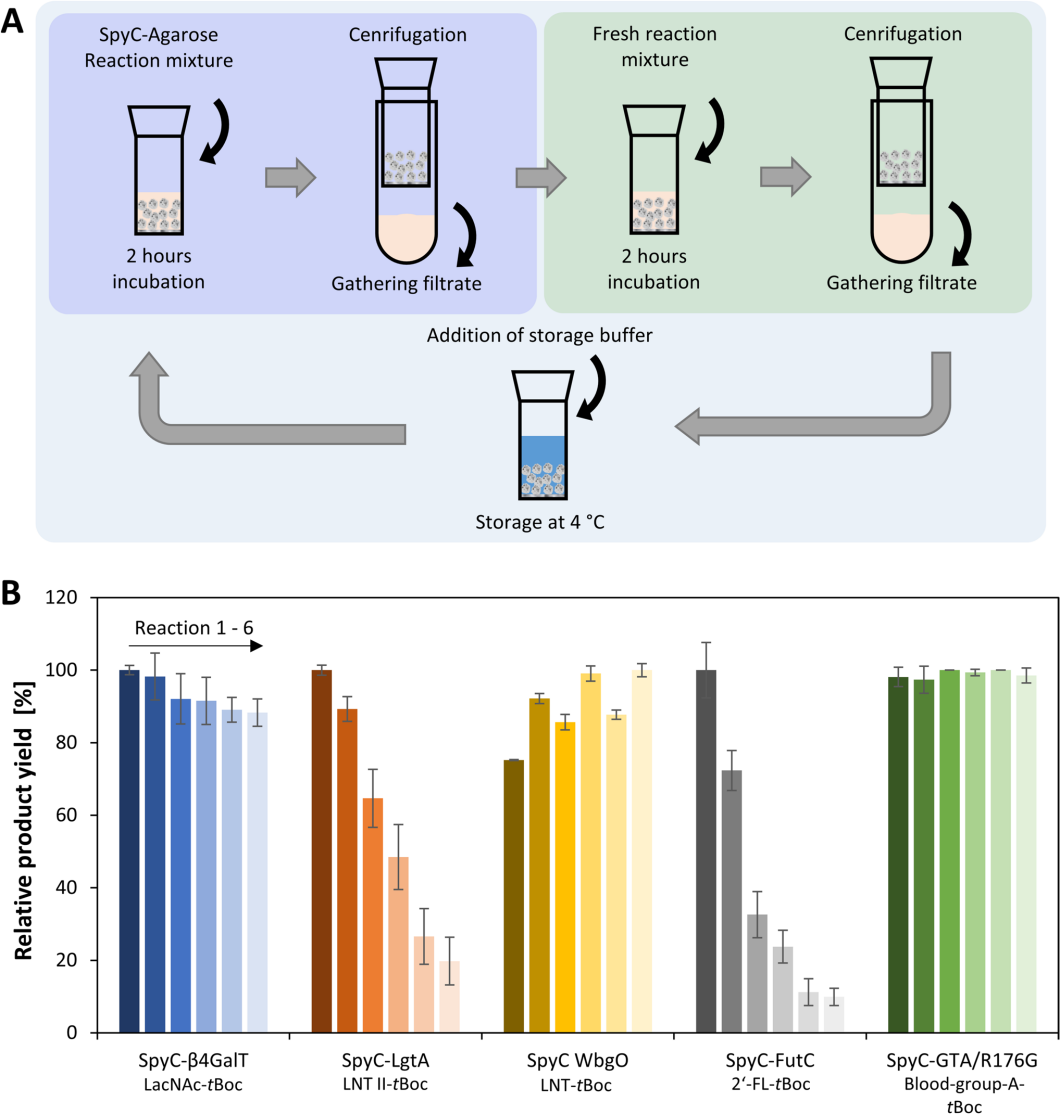
Assessment of reusability of SpyT-agarose immobilized SpyC-GTs. **A** Schematic depiction of the experimental procedure. The reaction mixture was added to the SpyC-GT agarose, incubated for 2 h, centrifuged, and the filtrate was gathered for analytics. For the second reaction, fresh reaction mixture was added. SpyC-GT agaroses were stored at 4 °C in the respective storage buffer. The cycle was repeated on 3 days. **B** Comparison of enzyme reusability. Values of absolute product yields were normalized to the highest yield of all six reactions (see SI, Table S12)

For each SpyT-agarose immobilized SpyC-GT, an assessment of reusability was done for six consecutive reactions over three days (Fig. 6A). The product formation (SI, Fig. S21-S25) was analyzed, and the absolute and relative product yields were compared (Fig. 6B and SI, Fig. S26 and Table S12). SpyC-GTA/R176G shows the highest reusability, as the relative product yields vary in a range from 97.4 to 100%. Similarly, SpyC-β4GalT retained sufficient activity with an 11.7% reduction in relative product yield. SpyC-WbgO exhibited an increased product yield throughout the reaction cycles. Starting with 75.2% relative product yield, the last reaction shows an activity increase of nearly 25%. In contrast, SpyC-LgtA shows a loss of activity during the six reaction cycles. While the relative product yield of SpyC-LgtA decreased by nearly 11% after the first reaction, only 19.8% of the initial product yield was left on the third day. SpyC-FutC also continuously declines in activity with achieving 10% of its initial relative product yield. In general, SpyT-agarose immobilized SpyC-FutC shows very low activity with only 1.5% absolute product formation during 2 h.

### Syntheses of Lacto-N-glycans

#### One-pot syntheses

**Fig. 7:**
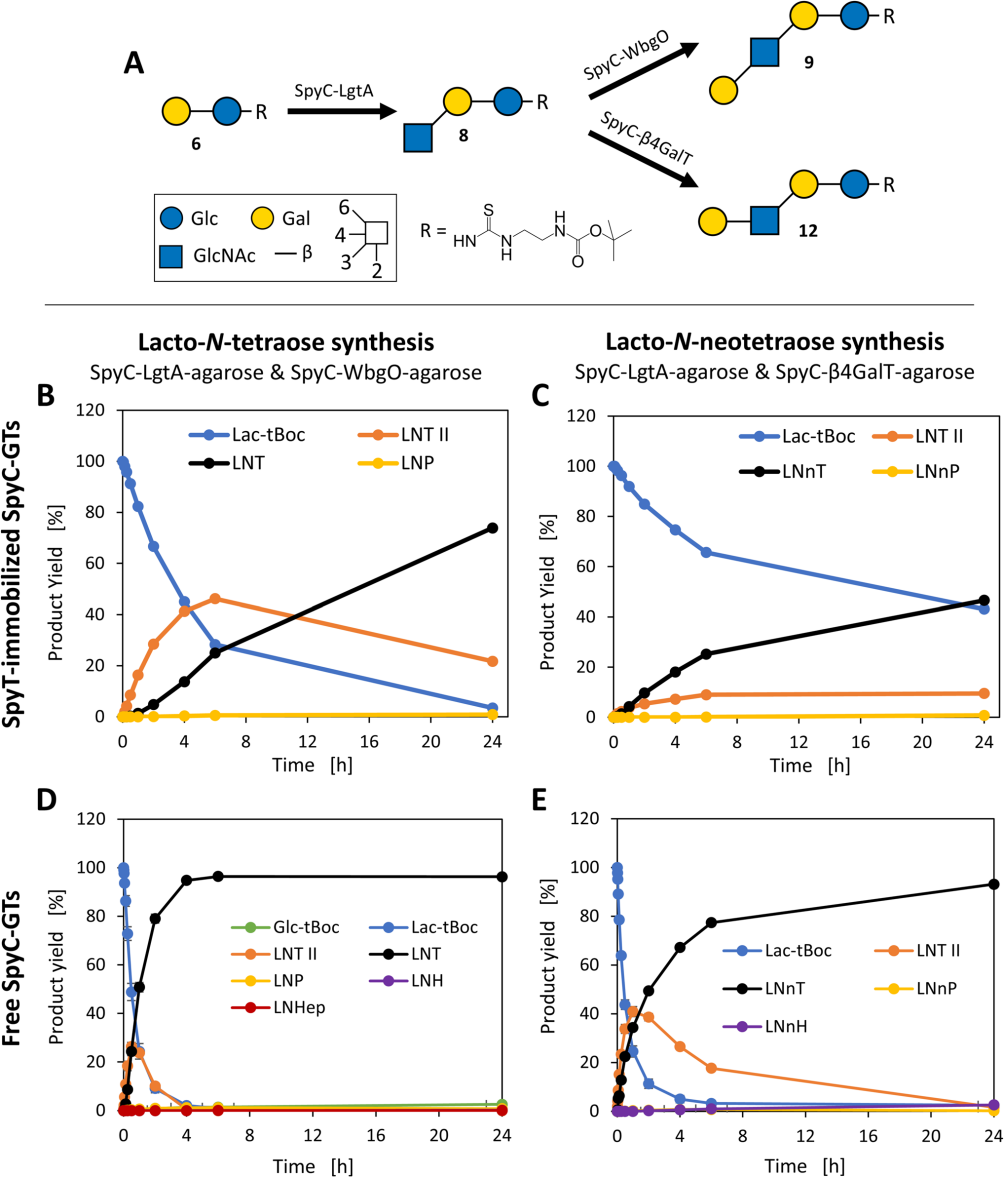
Glycan syntheses with SpyC-GTs. **A** Schematic depiction of LN(n)T syntheses. Lac-*t*Boc (**6**) is converted to LNT II-*t*Boc (**8**) by SpyC-LgtA, and further elongated either by SpyC-WbgO to LNT-*t*Boc (**9**) or SpyC-β4GalT to LNnT-*t*Boc (**12**). Visualization according to Symbol Nomenclature for Glycans (SNFG) is explained in Fig. 1. **B** Synthesis with immobilized SpyC-WbgO and SpyC-LgtA. Yields after 24 h: Lac-*t*Boc: 4%, LNT II: 22%, LNT: 74%, LNP: < 1%. **C** Synthesis with SpyT-agarose immobilized SpyC-β4GalT and SpyC-LgtA. Yields after 24 h: Lac-*t*Boc: 43%, LNT II: 10%, LNnT: 47%, LNnP: < 1%. **D** Synthesis with free SpyC-WbgO and SpyC-LgtA. Yields after 24 h: Glc-*t*Boc: 3%, Lac-*t*Boc: < 1%, LNT II: < 1%, LNT: 96% (reached after 3 h), LNP < 1%, LNHex: < 1%, LNHep: < 1%. **E** Synthesis with free SpyC-β4GalT and SpyC-LgtA. Yields after 24 h: Lac-*t*Boc: 2%, LNT II: 2%, LNnT: 93%, LNnP: < 1%, LNnH: 3%

As a proof-of-principle for the selective glycan synthesis with SpyT-agarose immobilized SpyC-GTs, one-pot syntheses of lacto-*N*-glycans were performed. To assess the impact of immobilization on the product formation, comparative reactions using free SpyC-GTs were also performed. Lac-*t*Boc (**6**) is first converted to LNT II-*t*Boc (**8**) by SpyC-LgtA, and subsequently galactose is added either in β1,3 or β1,4 linkage by SpyC-WbgO or SpyC-β4GalT, resulting in LNT-*t*Boc (**9**) or LNnT-*t*Boc (**12**), respectively (Fig. 7A). These tetrasaccharides are further elongated by the subsequent addition of GlcNAc and Gal, leading to the formation of pentaoses (LNP and LNnP), hexaoses (LNH and LNnH), and heptaoses (LNHep). Trials before this work showed that a ratio regarding specific activity for SpyC-WbgO and SpyC-LgtA of 1:5 yields the highest LNT-*t*Boc amount (SI, Fig. S27A). Based on our preliminary findings, the reaction conditions for the synthesis with SpyC-β4GalT and SpyC-LgtA (both glycine, pH 10) were chosen with a ratio of 2:1 (SI, Fig. S27B).

The synthesis with immobilized SpyC-WbgO and SpyC-LgtA reaches a maximum yield of 46% LNT II-*t*Boc (**8**) within the first 6 h (Fig. 7B). After 24 h, the amount decreased to 22% due to further conversion to LNT-*t*Boc (**9**), which increased almost Linearly throughout the reaction, resulting in a yield of 74% (1.7 mM) after 24 h. In addition, 4% residual Lac-*t*Boc (**6**) and LNP-*t*Boc, with less than 1% were detected (Fig. 7B and SI, Fig. S28). The synthesis with free SpyC-GTs shows LNT II-*t*Boc (**8**) peaking at 26% after 30 min with a subsequent decrease. During the first 2 h, the LNT-*t*Boc (**9**) content increased up to 79%, and after 6 h, the product yield reached 96% (4.9 mM). In addition to residual Lac-*t*Boc (**6**) and LNT II-*t*Boc (**8**), the glycans LNP-*t*Boc, LNH-*t*Boc, and LNHep-*t*Boc were detected, all three yielding less than 1% (Fig. 7D and SI, Fig. S29).

In the synthesis with immobilized SpyC-β4GalT and SpyC-LgtA, the LNnT-*t*Boc (**12**) content exceeds that of LNT II-*t*Boc (**8**) already after 2 h (Fig. 7C). After 24 h, the LNnT-*t*Boc yield reaches 47% (1.1 mM) with 43% residual Lac-*t*Boc (**6**) and 10% LNT II-*t*Boc (**8**). In addition to Lac-*t*Boc (**6**), LNT II-*t*Boc (**8**), and LNnT-*t*Boc (**12**), the formation of LNnP-*t*Boc was also detected (SI, Fig. S30). In the synthesis using free SpyC-GTs, the LNT II-*t*Boc (**8**) content peaks at 41% after 1 h and decreases thereafter (Fig. 7E). The content of LNnT-*t*Boc (**12**) increases to 77% after 6 h, and yields 93% (2.9 mM) after 24 h. In addition to low levels of residual Lac-*t*Boc (**6**) and LNT II-*t*Boc (**8**), the formation of LNnP-*t*Boc and LNnH-*t*Boc was detected (Fig. 7E and SI, Fig. S31).

#### Sequential syntheses by a combination of immobilized and free GTs

**Fig. 8:**
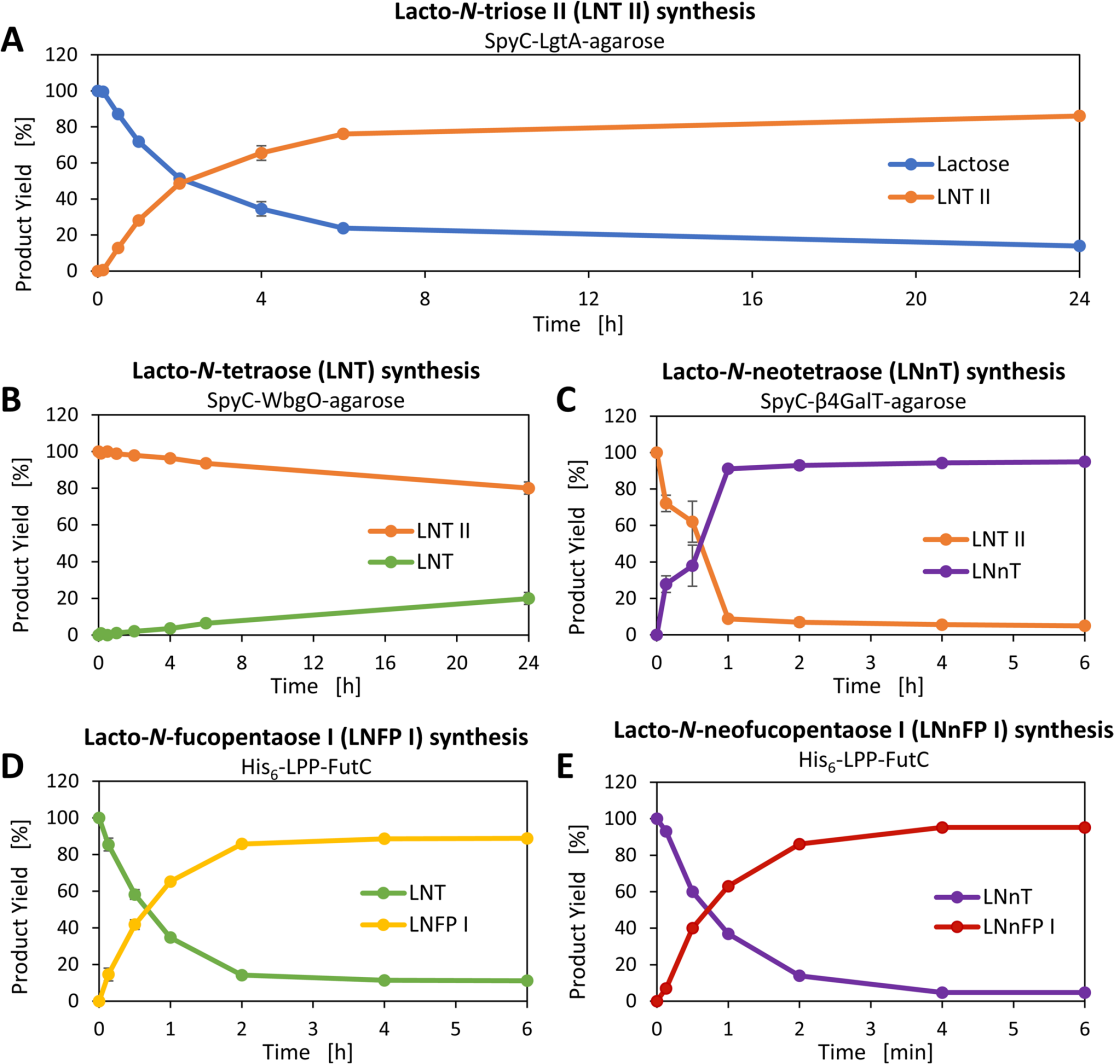
Sequential glycan syntheses (SI, Fig. S32-S38). **A** LNT II synthesis with SpyT-agarose immobilized SpyC-LgtA. LNT II yield after 24 h: 86%.** B** LNT synthesis with SpyT-agarose immobilized SpyC-WbgO. LNT yield after 24 h: 19%. **C** LNnT synthesis with SpyT-agarose immobilized SpyC-β4GalT, LNnT yield after 1 h: 91%. **D** LNFP I synthesis with His_6_-LPP-FutC. LNFP I yield after 2 h: 85%. **E** LNnFP I synthesis with His_6_-LPP-FutC. LNnFP I yield after 2 h: 86%

In addition to the one-pot syntheses, sequential syntheses were carried out. Starting with plain lactose, the products LNT (**9’**, Fig. 1B) and LNnT (**12’**) were additionally fucosylated (compounds **10’** and **13’**), and further converted to BGA hexaose type I (**11’**) and BGA hexaose II (**14’**) (SI, Fig. S32-S38). Moreover, for analytical purposes, this sequential synthesis was also performed with only free SpyC-GTs and Lac-*t*Boc (**6**) (SI, Fig. S39-S45). The LNT II (**8’**, Fig. 1B) synthesis reached a product yield of 86% after 24 h (Fig. 8A). In the LNT (**9’**) synthesis with SpyC-WbgO agarose, a product yield of 20% was achieved after 24 h (Fig. 8B). During the LNnT (**12’**) synthesis with SpyC-β4GalT agarose, a product yield of 91% was achieved after 1 h (Fig. 8C). The syntheses of LNFP I (**10’**) and LNnFP I (**13’**) show high yields of 85% (Fig. 8D) and 86% (Fig. 8E), respectively, after 2 h. For the BGA hexaose syntheses with immobilized SpyC-GTA/R176G, BGA hexaose type I (**11’**) could not be detected as a distinguished peak in the CE-LIF measurement (SI, Fig. S37), but we measured the full conversion of LNnFP I (**13’**) to BGA hexaose type II (**14’**) after 1 h (SI, Fig. S38).

The sequential syntheses with free SpyC-GTs and Lac-*t*Boc as the initial acceptor substrate resulted in a yield of over 90% for nearly all products after 30 min. Only for LNFP I (**12**), this value was reached after 2 h (SI, Fig. S46). All products are confirmed by HPLC-ESI-MS (Table 2 and SI, Fig. S47-S53).

**Table 2:**
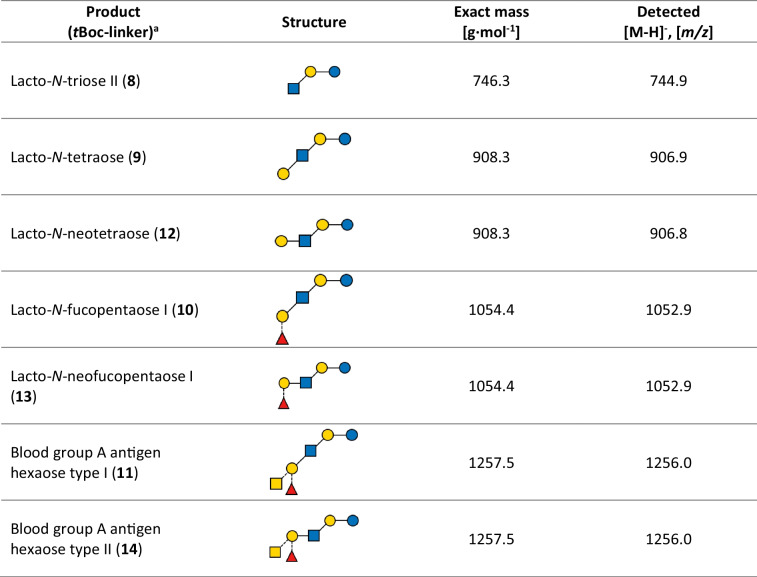
Mass spectrometry analysis of the glycans obtained by sequential synthesis. HPLC-ESI-MS product detection was done in negative mode (SI, Fig. S47-S53). Visualization according to Symbol Nomenclature for Glycans (SNFG) is explained in Fig. 1, blue circle: Glc; yellow circle: Gal; blue square: GlcNAc; yellow square: GalNAc; red triangle: Fuc

^a^ Numbers in parentheses depict the compounds in Fig. 1B

## Discussion

### Production of SpyC-GTs

In the immunoblots of the SpyC-GTs (Fig. S2, S4, S6, S8, and S10), additional lower-molecular-weight bands are observed. These likely arise from incomplete translation products or from partial proteolytic degradation during sample handling and storage, as the N-terminal His_6_-tag is detected. Such species may slightly reduce the apparent specific activity, resulting in an underestimation of the *k*_cat_, and can be immobilized on the SpyT-agarose. However, since the agarose has a high capacity that is not fully occupied during the preparation, we expect no limitations for the immobilization of active GTs.

Relocating the His_6_-tag to the C-terminus and optimization of expression and purification conditions could minimize these effects, but this was beyond the scope of the present study. Moreover, some of our previous trials indicated a complete deactivation of Leloir-GTs by a C-terminal His_6_-tag.

Despite these considerations, the general construct of the SpyC-GTs appears well thought out, as all SpyC-GTs were expressed as soluble and active enzymes in *E. coli*.

### Characterization of SpyC-GTs

Finding the optimal buffer and pH for the highest possible activity is a key part of enzyme characterization, since this is one of the most influential reaction parameters. Furthermore, a cofactor screening gives valuable insights into enzyme characteristics. Depending on their protein fold, Leloir-GTs are categorized into the GT-A and GT-B superfamilies (Carbohydrate Active Enzyme database, CAZy, https://www.cazy.org/) (Drula et al. 2022). GT-A-fold GTs require divalent metal ions (e.g., Mn^2+^ or Mg^2+^) for their catalytic activity, while members of the GT-B family are cofactor-independent, as nucleotide sugar binding is mediated by basic amino acid residues (Lairson et al. 2008). Kinetic characterization is especially important in the planning of enzymatic syntheses, where a maximum outcome of product with a minimum input of enzyme is strived for. Moreover, substrate excess inhibitions can be detected and bottlenecks in multi-enzyme cascades can be identified, allowing adjustment of enzyme or substrate amounts for optimal product formation.

For SpyC-β4GalT, we could show the highest activity at pH 10 in a glycine-NaOH buffer (Fig. 2). Previous studies on the LPP-β4GalT fusion protein suggested an optimal pH value between 6.8 and 8 (Sauerzapfe et al. 2008). However, there were only two different buffer systems evaluated (50 mM citrate, pH 5–6.8 and 50 mM HEPES-NaOH, pH 6.8–8.2), not testing basic pH values and other buffer components. Moreover, these previous studies demonstrated that Mn^2+^ as a cofactor is preferred as a divalent metal ion. In this work, we also obtained increasing activity for SpyC-β4GalT with increasing Mn^2+^ concentrations, underscoring the previous findings. A substrate inhibition for GlcNAc-*t*Boc, as seen in this work, was also shown for other GlcNAc acceptor substrates carrying hydrophobic groups (Bn-GlcNAc, *p*NP-GlcNAc), whereas pure GlcNAc up to 50 mM did not inhibit β4GalT (Sauerzapfe et al. 2008).

In previous studies on LgtA, by screening the buffers MES pH 5–6.5, TRIS-HCl pH 7–9, and CAPS pH 10–11, a pH optimum at pH 7.8–8 with residual activity at pH 10 was shown. Moreover, a clear preference for Mn^2+^ over Mg^2+^ and Ca^2+^ or any other divalent cation with the highest activity at 10 mM Mn^2+^ was described (Li et al. 2016). In kinetic studies for several acceptor substrates, reactions with *p*NP-lactose resulted in a higher activity compared to plain lactose, suggesting a beneficial effect of hydrophobic tags (Blixt et al. 1999; Li et al. 2016). Our results show the highest activity at pH 10 with glycine-NaOH, suggesting that the SpyCatcher influences LgtA activity, as it shifts the pH optimum of the enzyme (Fig. 2). The cofactor screening of SpyC-LgtA showed no activity with EDTA in the reaction mix and an increasing activity with increasing Mn^2+^ concentration, underscoring the previous findings (Fig. 3). We could detect a substrate inhibition for Lac-*t*Boc concentrations above 6.5 mM (Table 1), whereas it was previously shown that 10 mM lactose does not inhibit LgtA reactivity. However, the authors did not further investigate substrate inhibition for any other acceptor substrate.

Earlier characterization studies on FutC were done for HEPES (Stein et al. 2008) or TRIS (Liu et al. 2022) buffers, neglecting other buffer substances and their respective buffer ranges (SI, Table S6). Moreover, structural analyses have classified FutC to belong to the GT-B superfamily (Sun et al. 2007). We could prove its independence on the divalent metal ions Mn^2+^ and Mg^2+^, exclude negative impacts of both ions, and measure activity throughout a pH range from 5.5 to 10. (Fig. 2 and Fig. 3).

While in earlier publications on GTA/R176G, the focus was laid on structural analyses and substrate preferences, kinetic data was obtained at pH 7 in the presence of 20 mM Mn^2+^, without further looking into different buffer systems, divalent metal ion preferences, and different pH values (Alfaro et al. 2008; Blackler et al. 2017; Lee et al. 2005). The reported turnover numbers (*k*_cat_) of 55 (Seto et al. 1997) and 48 (Alfaro et al. 2008; Lee et al. 2005) are similar to our results. For an optimal evaluation of the specific activity, a high dilution of SpyC-GTA/R176G is required. Especially during the screening with divalent metal ions, this necessity resulted in suboptimal reproducibility of the activity assays. However, under synthesis conditions above a pH of 7, the catalytic activity of SpyC-GTA/R176G far exceeds that of the other enzymes, regardless of the divalent metal ion concentration. Consequently, SpyC-GTA/R176G does not represent a bottleneck in the synthesis process, and the optimal concentrations of divalent metal ions for the other SpyC-GTs also enable high substrate conversion with SpyC-GTA/R176G. Interestingly, there was, to the best of our knowledge, no further investigation on the human GTA/R176G regarding its application in the synthesis of blood group A, impeding a complete comparison of the SpyC-GTA/R176G and the non-tagged version.

We could show that the SpyCatcher domain shifts the pH optimum of β4GalT and LgtA to basic pH values, increasing their activity, particularly in ranges from pH 8 to 10 (Fig. 2), compared to pH 6.8 to 8 for β4GalT and 7.8 to 8 for LgtA (Li et al. 2016; Sauerzapfe et al. 2008). Our kinetic data lay a profound basis for the application of SpyC-GTs in glycan synthesis, as we could identify substrate inhibition for SpyC-LgtA, SpyC-β4GalT, and SpyC-GTA/R176G (Table 1). While analytical tags, like the *t*Boc-linker, are a useful tool for fast and efficient analysis, they appear to have a significant influence on enzyme kinetics. Therefore, the choice of substrate is important.

### Immobilization of SpyCatcher-GTs

#### Immobilization

Our observations show a very well-working coupling procedure and imply that even higher amounts of SpyT can be coupled to the resin. The formation of the covalent iso-peptide bond of the SpyC-SpyT system was previously shown at pH ranges between 5 and 8 (Zakeri et al. 2012). SpyC-GTs were immobilized in various buffers ranging from pH 7.5 to 10. Hereby, the robustness of the SpyC-SpyT technology was further assessed. We could also expand the repertoire of immobilization techniques of GTs on polymeric resins (Sommerfeld et al. 2024).

#### Long-term stability

After 1 month, increased activities were observed for SpyC-β4GalT (38%) and SpyC-GTA/R176G (29%) (Fig. 5). The reasons for higher enzyme activities remain unclear, as the structural properties of the agarose matrix were not further investigated. Most probably, compression of the agarose resin during storage due to the porous-viscoelastic properties of the beads, the displacement of water from the pores, and breakage of agarose cross-links leads to a more compact structure (Caccavo et al. 2017; Ed-Daoui and Snabre 2021). Thus, an increased enzyme amount within the same resin volume may explain the increase in activity. In general, we could show that the immobilized SpyC-GTs retained more activity than their free counterparts, with SpyC-GTA/R176G being the only exception.

#### Reusability

The purpose of resin-immobilized enzymes is the application in reactors for operational modes like repetitive batch or continuous processes (Hussnaetter et al. 2023). The reusability of SpyC-β4GalT, SpyC-WbgO, and SpyC-GTA/R176G underscores our findings for long-term stability (Fig. 6), proving these as well-suited for immobilization and application in industrial processes. For SpyC-WbgO, the addition of 5 mM dithiothreitol (DTT) to the storage buffer was proposed for elevated storage stability (Fischöder et al. 2017). The observed activity pattern is most likely due to multiple buffer changes, as done here. The decline in SpyC-LgtA activity could be attributed to the oxidation of Mn^2+^ ions to manganese oxide (MnO_2_) at pH 10, hampering the activity. However, MnO_2_ formation was also observed for SpyC-β4GalT reactions, but no inactivating influence was measured. Since SpyC-LgtA appears to be more susceptible to MnO_2_, optionally, Mg^2+^ could be used as a cofactor. For SpyC-FutC, the multiple buffer changes for reaction and storage, and the temperature changes from 4 to 30 °C seem to impact the structural integrity, leading to a high loss of activity.

### Syntheses of Lacto-N-glycans

#### One-pot syntheses

For SpyC-WbgO, previous studies have shown optimal activity in HEPES pH 7.5 (Fischöder et al. 2017). To evade possible limitations due to the less active SpyC-WbgO, this reaction buffer was chosen. In both cases, the reaction with immobilized enzymes shows a lower product yield than that with free enzymes (Fig. 7). Additionally, an enzyme ratio of SpyC-β4GalT 2:1 SpyC-LgtA leads to a bottleneck in the synthesis with immobilized enzymes, as an insufficient amount of intermediate LNT II-*t*Boc is formed. Regarding the overall lower activity of immobilized SpyC-GTs, the enzyme amount and optimal enzyme ratio should be reconsidered to enhance the product yield. Also, in this work, the synthesis of longer linear glycan structures was detected in HPLC analysis (Fig. 7), similar to previous studies on poly-LacNAc type II oligomer syntheses. Here, poly-LacNAc oligomers of up to six LacNAc units, predominated by unevenly numbered glycans, were described for *Hp*β3GlcNAcT and β4GalT (Rech et al. 2011). For both enzyme combinations with SpyC-LgtA, this elongation capability is only seen to a very minor extent, while the even-numbered tetraoses predominate regardless of the enzyme ratio, as was seen in our previous experiments (SI, Fig. S27).

#### Sequential syntheses by a combination of immobilized and free GTs

The focus here was not on the enzyme interplay, but on the combinations of the best GT candidates (free or immobilized) for the sequential synthesis of HMOs (Fig. 1B). While most reactions of the sequential syntheses succeed, the LNT synthesis lags far behind (Fig. 8). Since SpyC-WbgO has a low affinity for LNT II-*t*Boc, an influence of the linker seems most plausible. Here, it would be useful to investigate the acceptance of different linkers and labels (Cbz-linker, 2-aminobenzoic acid (2-AA), and 2-aminobenzamide (2-AB)). As we could not detect BGA hexaose type I (**11’**), but the *t*Boc-tagged product (**11**) was successfully produced using the free SpyC-GTA/R176G, we propose that the separation performance is not sufficient to separate LNFP I (**10’**) and BGA hexaose I (**11’**) in CE-LIF analysis. Here, other analytical methods (e.g., xCGE-LIF) could be more insightful for future syntheses (Kottler et al. 2013). To further increase product yields and improve purity during sequential syntheses, either additional nucleotide sugars can be added to the reaction mixture, or they could be regenerated in situ (Elling 2025).

Despite the increased stability, the synthesis with SpyT-agarose-immobilized SpyC-GTs cannot fully compete with the synthesis with free enzymes under the tested conditions (Fig. 7). This shows the limitations imposed by immobilization, which affect the reaction environment and activity. The generally slower synthesis process with SpyT-agarose-immobilized SpyC-GTs could be attributed to conformational changes and diffusion limitations. While immobilized enzymes are restricted in their mobility, this can also affect other reaction compounds. This phenomenon was already described as mass-transfer effects (Tischer and Kasche 1999). Here, internal transfer limitations appear inside the agarose network. Moreover, the partition effect can lead to concentration gradients forming inside and outside of the particles. The diffusion of substrates within the reaction volume is reduced, limiting their availability to the immobilized enzymes. In addition, the agarose settles to the bottom of the reaction vessel throughout the reaction, while residual dissolved substrates remain above the agarose, further restricting their accessibility to the enzymes.

Our findings demonstrate that SpyCatcher-SpyTag immobilization enhances long-term stability and reusability of Leloir-GTs for various applications and provides valuable insights for the development of immobilized enzyme systems, underscoring the importance of optimizing enzyme selection, immobilization techniques, and reaction conditions. To improve the product yield, homogenization of the agarose and reaction solution by continuously shaking in an end-over-end shaker or employing a (semi-) continuous process with a fixed flow rate or in pulsed bursts should be considered. Also, the choice of the reaction vessel affects the conversion. A cylindrical vessel, as used for the reusability assay, which has the same base area over its entire height, distributes the agarose bed more evenly over the entire reaction space than a conical Eppendorf vessel, which tapers at the bottom. Additionally, increasing the amount of enzymes immobilized on the agarose could compensate for diffusion limitations. Therefore, the enzyme amount and ratio should also be considered when further refining sequential or one-pot synthesis processes.

## Conclusion

In this study, the immobilization of Leloir-GTs via the SpyCatcher-SpyTag system was evaluated for its influence on enzyme characteristics, reaction parameters, and glycan synthesis. Five different GTs were equipped with the SpyCatcher and actively expressed in *E. coli*. Characterization of the four enzymes SpyC-β4GalT, SpyC-LgtA, SpyC-FutC, and SpyC-GTA/R176G revealed the optimal reaction parameters, including pH values and cofactor concentrations. The kinetic parameters illustrated the very different activities of the SpyC-GTs. The immobilization onto maleimide-activated agarose using the SpyCatcher-SpyTag immobilization system was demonstrated to be highly efficient, and immobilized SpyC-GTs were shown to have improved storage stability and reusability, enabling convenient and cost-effective handling. The SpyT was coupled with an efficiency of over 99%. The coupling efficiency of the SpyC-GTs ranged from 67% (SpyC-LgtA) to 100% (SpyC-GTA/R176G). The long-term stability of all five SpyC-GTs was observed over 1 month, and an increased stability of SpyT-agarose immobilized SpyC-GTs compared to the free enzymes was measured in all cases. Experiments on the reusability of immobilized SpyC-GTs showed that especially SpyC-β4GalT, SpyC-WbgO, and SpyC-GTA/R176G can be reused for several reactions on three consecutive days. Despite immobilized enzymes showing higher stability, one-pot syntheses with immobilized enzymes achieved lower product yields compared to those with free SpyC-GTs. Moreover, the initial acceptor substrate influences the enzyme’s performance, as a hydrophobic tag like the *t*Boc-linker can impact the enzymatic activity, and with that, the initial acceptor substrate concentration has to be carefully adjusted. In sequential syntheses, we were able to produce the hexasaccharides blood group A antigen hexaose types I and II.

The results confirm the overall potential of immobilized SpyC-GTs for use in enzymatic syntheses despite the challenges posed by diffusion limitations. Further adaptations and optimizations of the reaction parameters may be required and could tackle these obstacles to increase productivity. The versatility and flexibility of the immobilization approach presented in this study enable the implementation of various sequential or one-pot reaction cascades and pave the way for further applications with immobilized GTs, offering a promising avenue for producing specific HMOs and glycans in general at larger scales in (semi-) automated, versatile, and scalable processes.

## Supplementary Information

Below is the link to the electronic supplementary material.

Supplementary material 1

## Data Availability

The authors declare that data supporting the findings of this study are available within the article and its supplementary information or can be requested from the corresponding author.
