# Supplementary material for: Glycan synthesis with SpyCatcher-SpyTag immobilized Leloir-glycosyltransferases
Source: Appl Microbiol Biotechnol. 2025 Oct 24;109:234. doi: 10.1007/s00253-025-13602-2 (PMC12552311; doi:10.1007/s00253-025-13602-2)
Supplement: Supplementary file 1 [file 253_2025_13602_MOESM1_ESM.pdf]

Supplementary Information

Glycan Synthesis with SpyCatcher-SpyTag immobilized Leloir-glycosyltransferases

Philip Palm, Kai Philip Hussnaetter, Lothar Elling*

Laboratory for Biomaterials, Institute of Biotechnology and Helmholtz-Institute for Biomedical Engineering, RWTH Aachen University, Aachen, Germany

*corresponding author. E-mail address: l.elling@biotec.rwth-aachen.de

Table of contents

1. **Additional Materials and Methods S2**
2. **Schemes S6**
3. **Sequence information S7**
4. **Tables S9**
5. **Figures S16**
6. **References S43**

1. Additional materials and methods

Glycan acceptor substrates and nucleotide sugars

The acceptor substrates GlcNAc-*t*Boc (**1**) and Lactosyl-*t*Boc (**6**) (Scheme S1) were kindly provided by Prof. Ing. Vladimír Křen, PhD., DSc., FRSC, and Doc. RNDr. Pavla Bojarová, PhD, from the Laboratory of Biotransformation at the Institute of Microbiology of the Czech Academy of Sciences in Prague (Sauerzapfe et al. 2009; Slamova et al. 2023).

For activity assays of SpyC-GTA/R176G using the H-antigen type V-*t*Boc (also referred to as 2'-fucosyllactose-*t*Boc, 2'-FL-*t*Boc, **7**, Fig. 1B) as acceptor substrate and for glycan syntheses starting with Lacto-*N*-triose II (LNT II-tBoc, **8**), provided precursor substrates were enzymatically converted to the respective acceptor substrate: 2'-FL-*t*Boc (**7**) was synthesized using Lactosyl-*t*Boc (5 mM) (Sauerzapfe et al. 2009; Slamova et al. 2023) and GDP-Fuc (6.5 mM), in-house synthesis as explained below (Frohnmeyer et al. 2022). The reaction mixture contained 100 mM Tris-HCl (pH 6.0), 25 mM KCl, 5 mM $MnCl_2$, 5 mM $MgCl_2$, 3 U FastAP, and 200 µg/mL FutC (non-SpyCatcher His_6-LPP-tagged version). After 24 hours of incubation at 30 °C, the reaction was terminated at 95 °C for 5 min. The reaction products were purified using a Sep-Pak C18 cartridge (Waters, Milford, MA, USA) according to the manufacturer's protocol. In short, the column was equilibrated with 50 % methanol in water, and the reaction mixture, dissolved in water, was loaded onto the column. The flow-through was discarded, and unbound hydrophilic components were washed away with water. The bound, synthesized oligosaccharides were eluted with 50 % acetonitrile. The eluate was concentrated using a vacuum rotary evaporator and subsequently lyophilized. Further purification and removal of precursors were performed by size-exclusion chromatography using a BioGel P2 column (BioRad, Hercules, CA, USA). Here, the lyophilized oligosaccharides were diluted in water and applied to the BioGel P2 column (column height 100 cm, column diameter 2.6 cm, bed volume 530 mL). Purification was carried out in water at 0.125 $mL\cdot min^{-1}$, gathering fractions of 2 mL. HPLC analysis (as described below) was performed for each fraction, and the fractions containing the target compound were collected, pooled, and again lyophilized.

The acceptor substrate LNT II-*t*Boc (**8**, Fig. 1B) was produced in a two-step repetitive batch synthesis with a reaction volume of 10 mL for each batch. The initial batch contained 100 mM glycine, pH 10, 5 mM $MnCl_2$, 6.5 mM UDP-GlcNAc, 5 mM Lactosyl-*t*Boc (**6**), 20 U FastAP, and 0.3 $mg\cdot mL^{-1}$ SpyC-LgtA. The reaction was carried out in a VivaSpin centrifugal concentrator (30 kDa MWCO) at 30 °C and a horizontal rotation of 100 rpm. The reaction was stopped after 4 hours by centrifugation (8525 × g, 4 °C) to a residual volume of 1.5 mL. The second batch was started by the addition of fresh substrate solution (100 mM glycine, pH 10, 5 mM $MnCl_2$, and 6.5 mM UDP-GlcNAc). For maximum product formation, 20 U FastAP and 3 mg SpyC-LgtA were added during the second batch. After 4 hours, batches 1 and 2 were pooled, and 3.5 mM UDP-GlcNAc was added. After another 16 hours of incubation, the synthesis was stopped again by centrifugation (4 °C and 8525 × g). For volume reduction, the filtrate was lyophilized, resuspended in MQ-Water, and centrifuged for pelletizing insoluble particles (10 min, 4 °C, 8525 × g). The supernatant was applied to a BioGel P2 column, and LNT II was purified by size-exclusion chromatography as stated above. Fractions containing the target compound were again collected, pooled, and lyophilized for storage at -20 °C.

In general, the nucleotide sugars were purchased from suppliers (UDP-Gal and UDP-GlcNAc: Biosynth, formerly Carbosynth, Staad, Switzerland; UDP-GalNAc: Biolog Life Science Institute, Bremen, Germany). GDP-Fuc was acquired through in-house synthesis based on the process described by Frohnmeyer et al. (2022), modified for a one-pot synthesis (Frohnmeyer et al. 2022). In short, the bifunctional enzyme L-fucokinase/GDP-fucose pyrophosphorylase (FKP) from *Bacteroides fragilis* (Yi et

al. 2009) was heterologously expressed in *E. coli* BL21 (DE3) and purified via IMAC. The elution fraction was concentrated using Vivaspin centrifugal concentrators (30 kDa MWCO, 45 min, 4 °C, 8525 × g) and buffer-exchanged into 50 mM Tris-HCl, 100 mM NaCl (pH 7.5). For the enzymatic synthesis of GDP-Fuc, a reaction mixture was prepared containing 100 mM HEPES (pH 8.0), 20 mM $MnCl_2$, 10 mM ATP, 10 mM GTP, 10 mM L-fucose, 1 U·mL^{-1} pyrophosphatase (PPase from *Saccharomyces cerevisiae*, Roche, Basel, Switzerland), and the purified FKP. The reaction was incubated at 4 °C for 72 h. Following incubation, the enzymes and precipitated pyrophosphate were removed by centrifugation, stopping the reaction (Vivaspin 30 kDa, 45 min, 4 °C, 5000 rpm). The filtrate containing GDP-Fuc was adjusted to pH 8.0 using NaOH. Residual nucleotides were degraded by the addition of 20 U FastAP and incubated for 2 h at 4 °C, followed by the addition of $MgCl_2$ to a final concentration of 100 mM. For purification of GDP-Fuc, the reaction mixture was supplemented with pre-chilled isopropanol (-80 °C) to a final concentration of 40 %, incubated at -80 °C for 30 min, and centrifuged (30 min, 4 °C, 5000 rpm). The supernatant, containing GDP-Fuc, was collected and further precipitated by adding isopropanol (-80 °C) to a final concentration of 90 %, followed by incubation at -80 °C for 30 min. GDP-Fuc was recovered by centrifugation (45 min, 4 °C, 7200-8525 × g), and the resulting pellet was lyophilized overnight. The dried GDP-Fuc was dissolved in 3 mL MQ water. The concentration of GDP-Fuc was determined using multiplexed capillary electrophoresis (MP-CE).

Glycan Analysis

HPLC (High-performance liquid chromatography)

HPLC samples were analyzed on an UltiMate 3000 system via Chromeleon 7.2.10 evaluation software (Thermo Fisher, Waltham, MA, USA). Samples were diluted 1:5 in MQ-H_2O, and glycans were separated on a Silica-C18 column (MultoKrom 100-5 C18, 250 × 4 mm, CS Chromatographie, Langerwehe, Germany) in a 15 % acetonitrile polar mobile phase for 30 min (monosaccharide-*t*Boc acceptor substrates) or 20 min, respectively (disaccharide-*t*Boc or longer acceptor substrates) at a flow rate of 1 mL·min^{-1}. *t*Boc-linked sugars were detected via UV measurement at a wavelength of 254 nm. The relative peak area of the educt and product peaks at given time points was used for the calculation of the enzyme activity. Here, volumetric activity (U·mL^{-1}) was derived from the linear regression slope of the first 10 % product formation. The specific enzyme activity (U·mg^{-1}) was then calculated with consideration of the protein concentration. One unit (U) is defined as the product amount in µmole that is formed by the enzyme in one minute.

CE-LIF (Capillary electrophoresis with LED-induced fluorescence detection)

Glycans based on plain lactose were labeled with 8-aminopyrene-1,3,6-trisulfonic acid (APTS) (Hennig et al. 2015) at the reducing end. For this, a 2 µL sample was mixed with 2 µL 2-Picoline borane complex solution (0.2 M picoline borane in DMSO) and 2 µL 20 mM APTS in 3.5 M citric acid. 1 mM maltose was used as an internal standard. The labeling mixture was incubated at 37 °C for at least 16 hours and subsequently stopped by the addition of 94 µL 80 % (v/v) acetonitrile. APTS-labeled glycans were analyzed on a 7100 CE system by Agilent Technologies, Santa Clara, CA, USA, using the software OpenLAB CDS Chemstation Edition 01.07 and a PVA-fused silica capillary (64.5 cm total length, 56 cm effective length, 50 µm diameter). Detection of APTS was done with a Zetalif LIF 5A-05 Detector (Adelis – former Picometrics, Grables, France) at 450 nm. Samples were diluted accordingly, injected at 30 mbar for 10 sec, and separated for 20 min at -20 kV with a CE-LIF running buffer consisting of 40 mM ε-aminocaproic acid and 0.02 % (w/v) hydroxypropyl methylcellulose at a pH of 4.5. Again, the relative peak area of the educt and product peaks at given time points was used for the calculation of the enzyme activity and product formation.

MP-CE (Multiplexed capillary electrophoresis)

GDP-Fuc concentration was determined using a cePRO 9600™ System MP-CE system (Advanced Analytical Technologies, Ames, Iowa, USA) (Wahl et al. 2016). This system allows for parallel analysis of 96 samples, employing an array of 96 fused silica capillaries (80 cm total length, 55 cm effective length, 50 µm diameter). The GDP-Fuc was diluted accordingly, injected at -48 mbar for 10 sec, and separated for 20 min at 8 kV with a CE running buffer consisting of 50 mM ammonium acetate at a pH of 9.2, containing 2 mM EDTA. Additionally, 1 mM para-aminobenzoic acid (PABA) and 1 mM para-aminophthalic acid (PAPA) were used as internal standards. Analytes were monitored at 254 nm and evaluated using the software pK_a-Analyzer (Advanced Analytical Technologies, Ames, Iowa, USA). As done for the CE-LIF analysis, product concentration was calculated using the peak areas.

HPLC-MS (HPLC-Mass spectrometry)

For further product verification, HPLC coupled to electrospray ionization mass spectrometry (HPLC-ESI-MS) was done using a Dionex HPLC (P680 Pump, ASI100 Automated Sample Injector, UVD170U UV detector) and a Thermo Finnigan Surveyor MSQ via Chromeleon 6.80 evaluation software (Thermo Fisher, Waltham, Massachusetts, USA). Reaction components and glycans were separated on a Silica-C18 column (MultoKrom 100-5 C18, 250 × 4 mm, CS Chromatographie, Langerwehe, Germany) in a 15 % acetonitrile polar mobile phase for 120 min at a flow rate of 0.2 $mL \cdot min^{-1}$. MS product detection, based on the mass/charge ratio (m/z), was done in negative mode with 4 kV needle voltage at 400 °C and 100 V cone voltage (Fischöder et al. 2019).

Characterization of SpyCatcher glycosyltransferases

SpyC-β4GalT, SpyC-LgtA, SpyC-FutC, and SpyC-GTA/R176G were characterized for their optimal buffer system, pH, and co-factor concentration. Moreover, donor- and acceptor-substrate kinetics were measured. For buffer system and pH characterizations, activity assays were conducted as stated above in the buffers MES-NaOH (pH 5.5, 6, 6.5), MOPS-NaOH (pH 6.5, 7, 7.5), HEPES-NaOH (pH 7, 7.5, 8), TRIS-HCl (pH 8, 8.5, 9), and glycine-NaOH (pH 9, 9.5, 10). The optimal co-factor concentration for manganese or magnesium ions ($MgCl_2$ or $MnCl_2$) was determined in a concentration range of 0 to a maximum of 20 mM. Additionally, reactions in the presence of 2 mM EDTA were carried out. Enzyme kinetics were measured at 30 °C reaction temperature. Activity assays for donor substrate kinetics (SpyC-β4GalT: UDP-Gal; SpyC-LgtA: UDP-GlcNAc; SpyC-FutC: GDP-Fuc; SpyC-GTA/R176G: UDP-GalNAc) were carried out with a constant acceptor-substrate concentration of 5 mM and varying donor concentrations (0.5, 1, 2.5, 5, 6.5, 8, 10, 15, 20, and 25 mM). For acceptor-substrate kinetics (SpyC-β4GalT: GlcNAc-*t*Boc (**1**); SpyC-LgtA: Lactosyl-*t*Boc (**6**); SpyC-FutC: LacNAc-*t*Boc (**3**); SpyC-GTA/R176G: 2'FL-*t*Boc (**7**)), the donor concentration was kept at 5 mM, whereas the acceptor concentration was varied (0.5, 1, 2.5, 5, 6.5, 8, 10, 15, 20, and 25 mM). The residual reaction components were chosen in similarity to the activity assays described above (Table S3). The kinetic parameters maximum reaction velocity (v_{max}) and Michaelis-Menten constant (K_M) were calculated with Microsoft Excel 2019 (Version 1808) using the Solver add-in for non-linear regression with a least-square-fit for the Michaelis-Menten equation. The inhibition constant K_i was calculated in a similar manner using the Haldane equation for single-substrate inhibition (Sonnad and Goudar 2004). The additional parameters turnover number (k_{cat}), catalytic activity $k_{cat} \cdot K_M^{-1}$, and optimal substrate concentration ($[S]_{opt}$) were calculated accordingly.

Long-term stability of immobilized SpyC-GTs

The evaluation of long-term stability was done over a one-month time course, during which the SpyT-agarose immobilized SpyC-GTs and non-immobilized SpyC-GTs were stored in their respective storage

buffer at 4 °C (Table S2). One day, one week, and one month after coupling, a 40 µL sample of the SpyC-GTs (10 µL for SpyC-GTA/R176G) was taken and applied for activity assay as stated above (see Table S2; acceptor substrates: SpyC-β4GalT: GlcNAc-*t*Boc (**1**), SpyC-LgtA: Lactosyl-*t*Boc (**6**), SpyC-WbgO: LNT II-*t*Boc (**8**), SpyC-FutC: Lactosyl-*t*Boc (**6**), SpyC-GTA/R176G: 2'-FL-*t*Boc(**7**)). The residual resin and non-immobilized SpyC-GTs were kept in storage conditions. For comparison of immobilized and non-immobilized SpyC-GTs, the specific activity was calculated as stated.

Reusability of immobilized SpyC-GTs

For the assessment of enzyme reusability, the product yields of immobilized SpyC-GTs were measured for a total of 6 reactions on 3 consecutive days. Therefore, SpyC-GT-agaroses (enzyme amounts of immobilized GTs: SpyC-β4GalT: 54 µg; SpyC-LgtA: 52 µg; SpyC-WbgO: 62 µg; SpyC-FutC: 66 µg; SpyC-GTA/R176G: 75 µg) were filled into a PureCube 1-step mini-column (CubeBiotech, Monheim, Germany) and 50 µL of the respective reaction mix (see Table S2; acceptor substrates: SpyC-β4GalT: GlcNAc-*t*Boc (**1**), SpyC-LgtA: Lactosyl-*t*Boc (**6**), SpyC-WbgO: LNT II-*t*Boc (**8**). SpyC-FutC: Lactosyl-*t*Boc (**6**), SpyC-GTA/R176G: 2'-FL-*t*Boc (**7**)) was added, yielding a total reaction volume of 100 µL. The column resembles a single reaction chamber and allows batch and repetitive batch operations on a laboratory scale. After an incubation of 2 hours, the reaction was stopped by centrifugation for 60-120 seconds at 600 × g. While the SpyC-GT-agarose is retained by the PVDF membrane (0.1-0.2 µm pore size) of the column, products and residual educts are filtered and subsequently processed for HPLC analysis. To start the second reaction cycle, 50 µL of the fresh reaction mixture was added to the SpyC-GT-agarose and again incubated for 2 hours. After the second centrifugation, 200 µL storage buffer was added to the SpyC-GT-agarose, and the whole column was stored at 4 °C overnight. Two reaction cycles of 2 hours each were repeated on the second and third day, again with storage overnight between days two and three. HPLC analysis was done for all six filtrates, and a comparison of the product yields was conducted.

2. Schemes

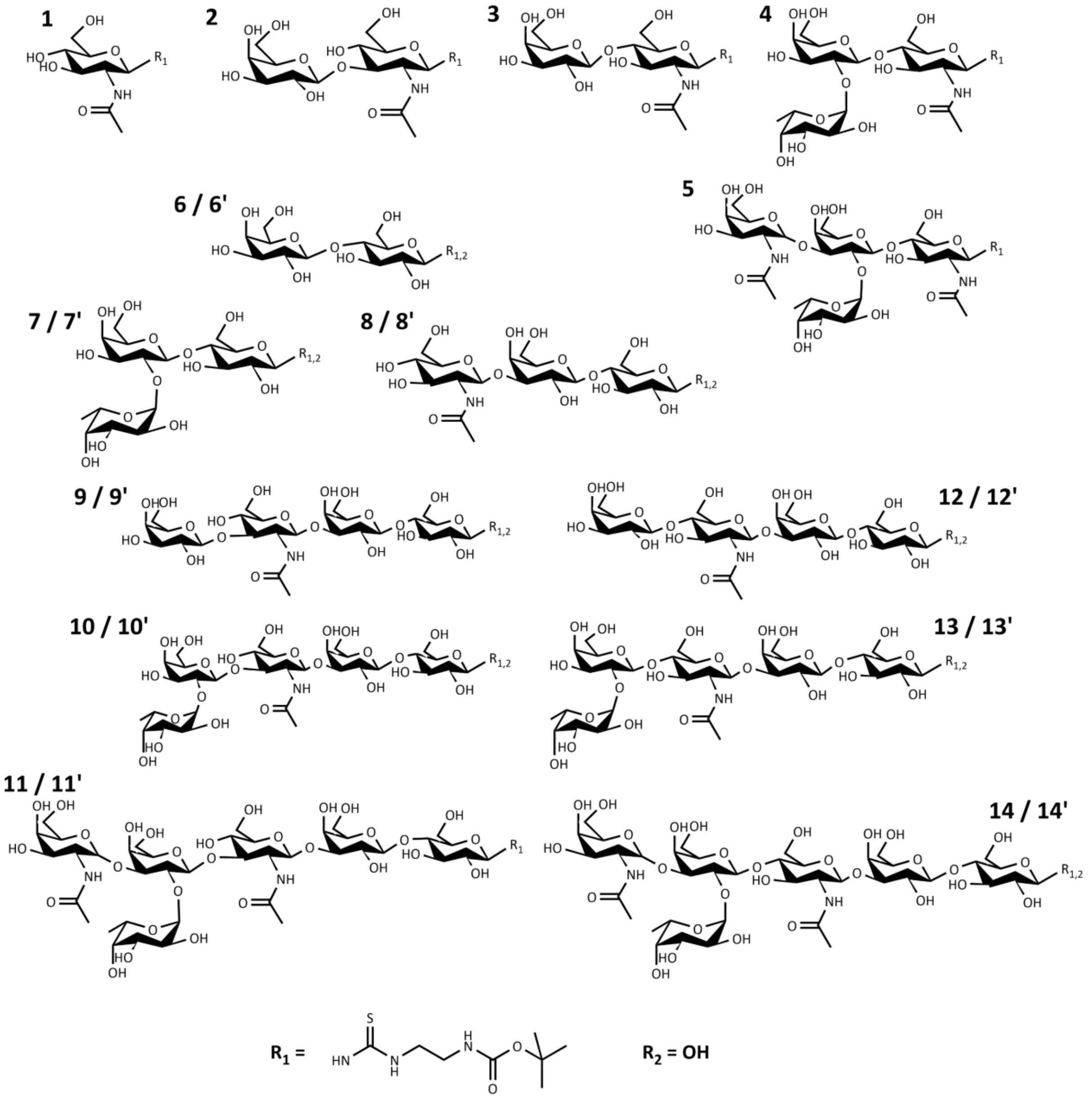

Scheme S1 Chemical structures of the synthesized/targeted glycans in this work. All glycans were synthesized with a *t*Boc-linker (R_1) for HPLC analysis: **1**: *N*-acetylglucosamine-*t*Boc (GlcNAc-*t*Boc); **2**: *N*-acetyllactosamine (LacNAc) type I-*t*Boc; **3**: LacNAc type II-*t*Boc; **4**: H-antigen type II-*t*Boc; **5**: Blood group A antigen (BGA) type II-*t*Boc; **6**: Lactosyl-*t*Boc; **7**: H-Antigen type V-*t*Boc; **8**: Lacto-*N*-triose II-*t*Boc (LNT II-*t*Boc); **9**: Lacto-*N*-tetraose-*t*Boc (LNT-*t*Boc); **10**: Lacto-*N*-fucopentaose I-*t*Boc (LNFP I-*t*Boc); **11**: Blood group A antigen hexaose type I-*t*Boc (BGA hexaose I-*t*Boc); **12**: Lacto-*N*-neotetraose-*t*Boc (LNnT-*t*Boc); **13**: Lacto-*N*-neofucopentaose I-*t*Boc (LNnFP I-*t*Boc); **14**: Blood group A antigen hexaose type II-*t*Boc (BGA hexaose II-*t*Boc); Moreover, glycans were synthesized without an analysis tag (R_2) and later on labeled with APTS for CE-LIF analysis: **6'** :lactose; **7'**: H-Antigen type V; **8'**: Lacto-*N*-triose II (LNT II); **9'**: Lacto-*N*-tetraose (LNT); **10'**: Lacto-*N*-fucopentaose I (LNFP I); **11'**: Blood group A antigen hexaose type I (BGA hexaose I); **12'**: Lacto-*N*-neotetraose (LNnT); **13'**: Lacto-*N*-neofucopentaose I (LNnFP I); **14'**: Blood group A antigen hexaose type II (BGA hexaose II)

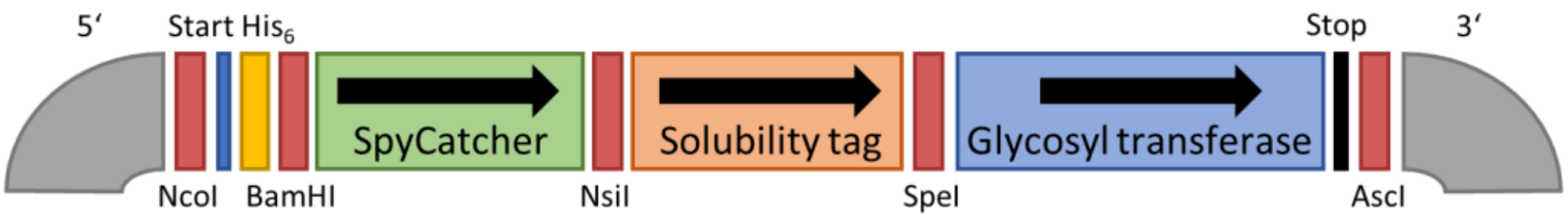

Scheme S2 General construct of the SpyCatcher glycosyltransferases. SpyC-GTs are embedded in the first multiple cloning site (MCS) of a pETDuet-1 vector. From 5' to 3', the segments are structured as follows: His_6-tag, SpyCatcher from *Streptococcus pyogenes*, solubility tag (namely the *Escherichia coli* K12 maltose binding protein (MBP) or the *Staphylococcus hyicus* lipase propeptide (LPP)), and the respective glycosyltransferase. Restriction sites were chosen for their unique appearance in the whole vector, facilitating a specific and convenient interchange of different solubility tags and glycosyltransferases

3. Sequence information

1	VTTLSGLSGEQGPSGDMTTEEDSATHIKFSKRDEDGRELAGATMELRDSSGKTISTWISD	60
61	GHVKDFYLYPGKYTFVETAAPDGYEVATPIEFTVNEDGQVTVDGEATEGDAHT	113

Sequence S1 Peptide sequence of the SpyCatcher003, coding sequence is part of expression constructs.

1	CGSGRGVPHIVMVDAYKRYK	20

Sequence S2 Peptide sequence of the SpyTag. Red: Cysteine for binding on maleimide group, Gray: spacer sequence, Blue: SpyTag003.

1	MGSSHHHHHHSQDPVTTLSGLSGEQGPSGDMTTEEDSATHIKFSKRDEDGRELAGATMEL	60
61	RDSSGKTISTWISDGHVKDFYLYPGKYTFVETAAPDGYEVATPIEFTVNEDGQVTVDGEA	120
121	TEGDAHTGSGGSGGSGMHNDSTTQTTTPLEVAQTSQQETHTHQTPVTSLHTATPEHVDDS	180
181	KEATPLPEKAESPKTEVTVQPSSHTQEVPALHKKTQQQPAYKDKTVPESTIASKSVESNK	240
241	ATENEMSPVEHHASNVEKREDRLETNETTPPSVDREFSHKIINNAHVNPKTDGQTNVNVD	300
301	TKTIDTVSPKDDRIDTAQPKQVDAPKENTTAQNKFTSQASQLRTGGARPPPPLGASSQPR	360
361	PGGDSSPVVDSGPGPASNLTSVPVPHTTALSLPACPEESPLLVGPMLIEFNMPVDLELVA	420
421	KQNPNVKMGGRYAPRDCVSPHKVAIIIPFRNRQEHLKYWLYYLHPVLQRQQLDYGVYVIN	480
481	QAGDTIFNRAKLLNVGFQEALKDYDYTCFVFSDVDLIPMNDHNAYRCFSQPRHISVAMDK	540
541	FGFSLPYVQYFGGVSALSKQQFLTINGFPNNYWGWGGEDDDIFNRLVFRGMSISRPNAVV	600
601	GRCRMIRHSRDKKNEPNPQRFDRIAHTKETMLSDGLNSLTYQVLDVQRYPLYTQITVDIG	660
661	TPS	663

Sequence S3 Protein sequence of the SpyC-β4GalT fusion protein. Purple: His_6-tag, Green: SpyCatcher, Gray: GS-Linker, Red: Propeptide, Blue: β4GalT. GenBank ID for β4GalT: X14085.1

1	MGSSHHHHHHSQDPVTTLSGLSGEQGPSGDMTTEEDSATHIKFSKRDEDGRELAGATMEL	60
61	RDSSGKTISTWISDGHVKDFYLYPGKYTFVETAAPDGYEVATPIEFTVNEDGQVTVDGEA	120
121	TEGDAHTGSGGSGGSGMHMKIEEGKLVIWINGDKGYNGLAEVGKKFEKDTGIKVTVEHPD	180
181	KLEEKFPQVAATGDGPDIIFWAHDRFGGYAQSGLLAEITPDKAFQDKLYPFTWDAVRYNG	240
241	KLIAYPIAVEALSLIYNKDLLPNPPKTWEEIPALDKELKAKGKSALMFNLQEPYFTWPLI	300
301	AADGGYAFKYENGKYDIKDVGVDNAGAKAGLTFLVDLIKNKHMNADTDYSIAEAAFNKGE	360
361	TAMTINGPWAWSNIDTSKVNYGVTVLPTFKGQPSKPFVGVLSAGINAASPNKELAKEFLE	420
421	NYLLTDEGLEAVNKDKPLGAVALKSYEEELAKDPRIAATMENAQKGEIMPNIPQMSAFWY	480
481	AVRTAVINAASGRQTVDEALKDAQTRITKGTSGMGQPLVSVLICAYNVEKYFAQSLAAVV	540
541	NQTWRNLDILIVDDGSTDGTLAIAQRFQEQDGRIRILAQPRNSGLIPSLNIGLDELAKSG	600
601	GGGEYIARTDADDIAAPDWIEKIVGEMEKDRSIIAMGAWLEVLSEEKDGNRLARHHEHGK	660
661	IWKKPTRHEDIADFFPFGNPIHNNTMIMRRSVIDGGLRYNTERDWAEDYQFWYDVSKLGR	720
721	LAYYPEALVKYRLHANQVSSKYSIRQHEIAQGIQKTARNDFLQSMGFKTRFDSLEYRQIK	780
781	AVAYELLEKHLPEEDFELARRFLYQCFKRTDTLPAGAWLDFAADGRMRRLFTLRQYFGIL	840
841	HRLLKNR	847

Sequence S4 Protein sequence of the SpyC-LgtA fusion protein. Purple: His_6-tag, Green: SpyCatcher, Gray: GS-Linker, Red: MBP, Blue: LgtA. GenBank ID for LgtA: AAC44084.1

1	MGSSHHHHHHSQDPVTTLSGLSGEQGPSGDMTTEEDSATHIKFSKRDEDGRELAGATMEL	60
61	RDSSGKTISTWISDGHVKDFYLYPGKYTFVETAAPDGYEVATPIEFTVNEDGQVTVDGEA	120
121	TEGDAHTGSGGSGGSGMHNDSTTQTTTPLEVAQTSQQETHTHQTPVTSLHTATPEHVDDS	180
181	KEATPLPEKAESPKTEVTVQPSSHTQEVPALHKKTQQQPAYKDKTVPESTIASKSVESNK	240
241	ATENEMSPVEHHASNVEKREDRLETNETTPPSVDREFSHKIINNAHVNPKTDGQTNVNVD	300
301	TKTIDTVSPKDDRIDTAQPKQVDAPKENTTAQNKFTSQASTSMIIDEAESAESTHPVVSV	360
361	ILPVNKKNPFLDEAINSILSQTFSSFEIIIVANCCTDDFYNELKHKVNDKIKLIRTNIAY	420
421	LPYSLNKAIDLSNGEFIARMDSDDISHPDRFTKQVDFLKNNPYVDVVGTNAIFIDDKGRE	480
481	INKTKLPEENLDIVKNLPYKCCIVHPSVMFRKKVIASIGGYMFSNYSEDYELWNRLSLAK	540
541	IKFQNLPEYLFYYRLHEGQSTAKKNLYMVMVNDLVIKMKCFFLTGNINYLFGGIRTIASF	600
601	IYCKYIK	607

Sequence S5 Protein sequence of the SpyC-WbgO fusion protein. Purple: His_6-tag, Green: SpyCatcher, Gray: GS-Linker, Red: Propeptide, Blue: WbgO. GenBank ID for WbgO: BAG11838.1

1	MGSSHHHHHHSQDPVTTLSGLSGEQGPSGDMTTEEDSATHIKFSKRDEDGRELAGATMEL	60
61	RDSSGKTISTWISDGHVKDFYLYPGKYTFVETAAPDGYEVATPIEFTVNEDGQVTVDGEA	120
121	TEGDAHTGSGGSGGSGMHNDSTTQTTTPLEVAQTSQQETHTHQTPVTSLHTATPEHVDDS	180
181	KEATPLPEKAESPKTEVTVQPSSHTQEVPALHKKTQQQPAYKDKTVPESTIASKSVESNK	240
241	ATENEMSPVEHHASNVEKREDRLETNETTPPSVDREFSHKIINNAHVNPKTDGQTNVNVD	300
301	TKTIDTVSPKDDRIDTAQPKQVDAPKENTTAQNKFTSQASTSMAFKVVQICGGLGNQMFQ	360
361	YAFAKSLQKHLNTPVLLDTTSFDWSNRKMQLELFPIDLPYANAKEIAIAKMQHLPKLVRD	420
421	ALKYIGFDRVSQEIVFEYEPKLLKPSRLTYFFGYFQDPRYFDAISSLIKQTFTLPPPPEN	480
481	NKNNNKKEEEYQRKLSLILAAKNSVFVHIRRGDYVGIGCQLGIDYQKKALEYMAKRVPNM	540
541	ELFVFCEDLKFTQNLDLGYPFTDMTTRDKEEEAYWDMLLMQSCKHGIIANSTYSWWAAYL	600
601	MENPEKIIIGPKHWLFGHENILCKEWVKIESHFEVKSQKYNA	607

Sequence S6 Protein sequence of the SpyC-FutC fusion protein. Purple: His_6-tag, Green: SpyCatcher, Gray: GS-Linker, Red: Propeptide, Blue: FutC. GenBank ID for FutC: AAC99764.1

1	MGSSHHHHHHSQDPVTTLSGLSGEQGPSGDMTTEEDSATHIKFSKRDEDGRELAGATMEL	60
61	RDSSGKTISTWISDGHVKDFYLYPGKYTFVETAAPDGYEVATPIEFTVNEDGQVTVDGEA	120
121	TEGDAHTGSGGSGGSGMHNDSTTQTTTPLEVAQTSQQETHTHQTPVTSLHTATPEHVDDS	180
181	KEATPLPEKAESPKTEVTVQPSSHTQEVPALHKKTQQQPAYKDKTVPESTIASKSVESNK	240
241	ATENEMSPVEHHASNVEKREDRLETNETTPPSVDREFSHKIINNAHVNPKTDGQTNVNVD	300
301	TKTIDTVSPKDDRIDTAQPKQVDAPKENTTAQNKFTSQASTSVREPDHLQRVSLPRMVYP	360
361	QPKVLTPCRKDVLVVTPWLAPIVWEGTFNIDILNEQFRLQNTTIGLTVFAIKKYVAFLKL	420
421	FLETAEKHFMVGHRVHYYVFTDQPAAVPRVTLGTGRQLSVLEV**G**AYKRWQDVSMRRMEMI	480
481	SDFCERRFLSEVDYLVCVDVDMEFRDHVGVEILTPLFGTLHPGFYGSSREAFTYERRPQS	540
541	QAYIPKDEGDFYYLGGFFGGSVQEVQRLTRACHQAMMVDQANGIEAVWHDESHLNKYLLR	600
601	HKPTKVLSPEYLWDQQLLGWPAVLRKLRFTAVPKNHQAVRNPHHHHH	607

Sequence S7 Protein sequence of the SpyC-GTA/R176G fusion protein. Purple: His_6-tag, Green: SpyCatcher, Gray: GS-Linker, Red: Propeptide, Blue: GTA, bold, underlined: mutated site R176G according to Seto (Seto et al. 1997). GenBank ID for GTA: Y11891.1

4. Tables

Table S1 Listing of the glycosyltransferases used in this work, and production and purification conditions. For all SpyC-GTs, the pET-Duet1 plasmid was used as an expression vector. All enzymes were expressed in *Escherichia coli* strains

Enzyme name	Enzyme type	Origin organism	N-terminal solubility tag	Expression strain	IMAC Buffer
SpyC-β4GalT	β1,4-galactosyl-transferase 1	*Homo sapiens*	Lipase pro-peptide (*S. hyicus*)	SHuffle T7 Express	20 mM Na_2HPO_4, 500 mM NaCl, 10, 500 mM Imidazole, pH 7.4
SpyC-LgtA	β1,3-*N*-acetylglucos-aminyltransferase	*Neisseria meningi-tidis*	Maltose binding protein (*E. coli* K-12)	Rosetta 2 (DE3) pLysS	50 mM TRIS-HCl, 500 mM NaCl, 30, 500 mM Imidazole, pH 7.5
SpyC-WbgO	β1,3-galactosyl-transferase	*E. coli* O55:H7	Lipase pro-peptide (*S. hyicus*)	BL21 (DE3)	20 mM Na_2HPO_4, 500 mM NaCl, 30, 500 mM Imidazole, pH 7.4 (0.2 % Triton-X-100 for elution)
SpyC-FutC	α1,2-fucosyltransferase	*Helico-bacter pylori*	Lipase pro-peptide (*S. hyicus*)	BL21 (DE3)	50 mM TRIS-HCl, 500 mM NaCl, 20, 500 mM Imidazole, pH 7.5
SpyC-GTA/R176G	α1,3-*N*-acetylgalactos-aminyltransferase	*H. sapiens*	Lipase pro-peptide (*S. hyicus*)	BL21 (DE3)	50 mM TRIS-HCl, 500 mM NaCl, 20, 500 mM Imidazole, pH 7.5

Table S2 Listing of the glycosyltransferases used in this work, and composition of storage buffers and reaction mixtures for glycosyltransferase activity assays. All Enzymes were obtained as described in Table S1, and activity assays were conducted in the described buffers

Enzyme name	Enzyme type	Storage buffer	Substrates	Reaction buffer
SpyC-β4GalT	β1,4-galactosyl-transferase 1	100 mM glycine, pH 10	UDP-Gal, GlcNAc-*t*Boc	100 mM Glycine pH 10, 5 mM $MnCl_2$, 6.5 mM UDP-Gal, 5 mM GlcNAc-*t*Boc, 3 U FastAP
SpyC-LgtA	β1,3-*N*-acetylglucos-aminyltransferase	100 mM glycine, pH 10	UDP-GlcNAc, Lactosyl-*t*Boc / lactose	100 mM Glycine pH 10, 5 mM $MnCl_2$, 6.5 mM UDP-GlcNAc, 5 mM Lactosyl-*t*Boc / lactose, 3 U FastAP
SpyC-WbgO	β1,3-galactosyl-transferase	50 mM NaH_2PO_4, 500 mM NaCl, 5 mM Dithiothreitol (DTT), pH 7.5	UDP-Gal, GlcNAc-*t*Boc	100 mM HEPES, 25 mM KCl, 5 mM $MgCl_2$, 6.5 mM UDP-Gal, 5 mM GlcNAc-*t*Boc, 3 U FastAP
SpyC-FutC	α1,2-fucosyl-transferase	50 mM TRIS-HCl, 100 mM NaCl, pH 7.5	GDP-Fuc, Lactosyl-*t*Boc / LacNAc-*t*Boc / lactose	100 mM TRIS-HCl pH 6, 25 mM KCl, 5 mM $MnCl_2$ and $MgCl_2$, 6.5 mM GDP-Fuc, 5 mM Lactosyl-*t*Boc / lactose, 3 U FastAP
SpyC-GTA/R176G	α1,3-*N*-acetylgalactos-aminyltransferase	50 mM TRIS-HCl, 100 mM NaCl, pH 7.5	UDP-GalNAc, 2'-FL-*t*Boc / 2'-FL	100 mM MOPS pH 7, 20 mM $MgCl_2$, 6.5 mM UDP-GalNAc, 5 mM 2'-FL-*t*Boc / 2'-FL, 3 U FastAP, 1 mg·mL^{-1} BSA

Table S3 Listing of the reaction components, donor, and acceptor substrates for the kinetic characterization of SpyC-GTs. Donor substrate kinetics were carried out with a constant acceptor-substrate concentration of 5 mM and varying donor concentrations (0.5, 1, 2.5, 5, 6.5, 8, 10, 15, 20, and 25 mM). Acceptor-substrate kinetics were carried out with a constant donor concentration of 5 mM and a varying acceptor concentration (0.5, 1, 2.5, 5, 6.5, 8, 10, 15, 20, and 25 mM)

Enzyme name	Acceptor	Donor	Reaction buffer
SpyC-β4GalT	GlcNAc-*t*Boc	UDP-Gal	100 mM glycine-NaOH pH 10, 5 mM $MnCl_2$, 5 U FastAP
SpyC-LgtA	Lactosyl-*t*Boc	UDP-GlcNAc	100 mM glycine-NaOH pH 10, 5 mM $MnCl_2$, 3 U FastAP
SpyC-FutC	LacNAc-*t*Boc	GDP-Fuc	100 mM TRIS-HCl pH 6, 25 mM KCl, 5 mM $MnCl_2$, 5 mM $MgCl_2$, 1 U FastAP
SpyC-GTA/R176G	2’-FL-*t*Boc	UDP-GalNAc	100 mM MOPS-NaOH pH 7, 20 mM $MgCl_2$, 1 U FastAP, 1 mg·mL^{-1} BSA

Table S4 Enzymatic activity of lyophilized and non-lyophilized SpyC-GTs. Purified SpyC-GTs were lyophilized, and a comparative activity assay was done for the lyophilized and the non-lyophilized enzymes

Enzyme	Acceptor substrate	Non-lyophilized [mU·mg^{-1}]	Lyophilized [mU·mg^{-1}]	Activity yield [%]
SpyC-β4GalT	GlcNAc-*t*Boc	732.54	677.5	92.5
SpyC-LgtA	Lactosyl-*t*Boc	899.15	843.34	93.8
SpyC-WbgO	LNT II-*t*Boc	346.88	374.17	107.8
SpyC-FutC	Lactosyl-*t*Boc	2.2	10.88	494.6
SpyC-GTA/R176G	2’-FL-*t*Boc	3559.76	3192.66	89.7

Table S5 Buffer system and pH screening for SpyC-GTs. Specific activity [$mU\cdot mg^{-1}$] in the dependency of buffer component and pH value is shown

**Enzyme**	**Buffer**	**pH**									
		**5.5**	**6**	**6.5**	**7**	**7.5**	**8**	**8.5**	**9**	**9.5**	**10**
SpyC-β4GalT	MES-NaOH	27.2 ± 1.5	29.76 ± 7.19	44.75 ± 2.16	-	-	-	-	-	-	-
	MOPS-NaOH	-	-	35.23 ± 0.5	41.84 ± 1.41	96.28 ± 0.97	-	-	-	-	-
	HEPES-NaOH	-	-	-	73.77 ± 9.32	88.02 ± 26.47	133.73 ± 17.28	-	-	-	-
	TRIS-HCl	-	-	-	-	-	98.83 ± 1.61	114.63 ± 1.86	126.25 ± 4.07	-	-
	Glycine-NaOH	-	-	-	-	-	-	-	149.51 ± 11.29	186.16 ± 9.99	285.33 ± 40.05
SpyC-LgtA	MES-NaOH	5.44 ± 0.31	106.03 ± 0.92	291.66 ± 21.16	-	-	-	-	-	-	-
	MOPS-NaOH	-	-	267.91 ± 2.34	283.28 ± 2.34	416.16 ± 37.09	-	-	-	-	-
	HEPES-NaOH	-	-	-	326.66 ± 12.53	329.63 ± 23	397.09 ± 1.16	-	-	-	-
	TRIS-HCl	-	-	-	-	-	425.25 ±0.75	279.53 ± 12.53	309.91 ± 7.03	-	-
	Glycine-NaOH	-	-	-	-	-	-	-	411.19 ± 14.88	393.38 ± 6.5	791.11 ± 9.25
SpyC-FutC	MES-NaOH	15.73 ± 8.93	17.25 ± 3.42	24.73 ± 2.25	-	-	-	-	-	-	-
	MOPS-NaOH	-	-	27.04	26.95 ± 1.97	23.11 ± 2.54	-	-	-	-	-
	HEPES-NaOH	-	-	-	22.27 ± 1.92	24.79 ± 2.93	23.43 ± 0.25	-	-	-	-
	TRIS-HCl	-	-	-	-	-	20.92 ± 0.38	18.26 ± 0.11	9.48 ± 1.18	-	-
	Glycine-NaOH	-	-	-	-	-	-	-	18.67 ± 0.53	16.41 ± 0.67	11.7 ± 0.81
SpyC-GTA/R176G	MES-NaOH	124.58 ± 8.56	298.37 ± 99.16	818.8 ± 229.79	-	-	-	-	-	-	-
	MOPS-NaOH	-	-	893.43 ± 285.1	1466 ± 669	327.31 ± 129.5	-	-	-	-	-
	HEPES-NaOH	-	-	-	1105 ± 252	1961 ± 80.64	1613 ± 93.29	-	-	-	-
	TRIS-HCl	-	-	-	-	-	2164 ± 763	2439 ± 707	3454 ± 422	-	-
	Glycine-NaOH	-	-	-	-	-	-	-	4537 ± 155	4734 ± 265	3932 ± 419

Table S6 Comparison of optimal buffer system and pH value for SpyC-GTs and their non-SpyC counterparts. Results for non-SpyC-GTs were gathered in previous publications. Results for SpyC-GTs were obtained for this work (see Table S5)

**Enzyme**	**Optimal buffer system and pH value**	
	**Non-SpyC-GT**	**SpyC-GT**
β4GalT	HEPES-NaOH pH 7.6 (Sauerzapfe et al. 2008)	Glycine-NaOH pH 10
LgtA	TRIS-HCl pH 8 (Naruchi et al. 2006)	Glycine-NaOH pH 10
FutC	HEPES-NaCl pH 5 (Stein et al. 2008)<br>TRIS-HCl pH 7.5 (Liu et al. 2022)	MOPS-NaOH pH 6.5-7
GTA/R176G	MOPS-NaOH pH 7 (Blackler et al. 2017)	Glycine-NaOH pH 9.5

Table S7 Co-factor screening for SpyC-GTs. Specific activity [mU·mg^{-1}] in the dependency of co-factor concentration and in the presence of EDTA is shown

**Enzyme**	**Co-factor**	**Concentration [mM]**									
		**0**	1	2	2.5	3.5	5	6.5	8	10	**20**
SpyC-β4GalT	Mn^{2+}	0	455 ± 11.99	589.6 ± 22.48	-	692.24 ± 54.94	751.8 ± 20.1	798.1 ± 44.44	921.99 ± 3.91	921.59 ± 3.11	-
SpyC-LgtA	Mn^{2+}	161.38 ± 1.6	596.64 ± 1.36	617.61 ± 10.75	-	640.43 ± 13	730.82 ± 29.89	818.25 ± 1.18	896.54 ± 41.11	908.79 ± 14.36	-
	EDTA	-	-	0	-	-	-	-	-	-	-
SpyC-FutC	EDTA	-	-	25.33 ± 1.39	-	-	-	-	-	-	-
	Mn^{2+}	-	-	-	25.79 ± 2.97	-	24.48 ± 0.43	-	-	20.55 ± 0.88	-
	Mg^{2+}	-	-	-	24.62 ± 0.24	-	24.19 ± 0.28	-	-	24.38 ± 0.53	-
	Mix	-	-	-	23.53 ± 0.46	-	21.68 ± 0.18	-	-	19.95 ± 0.73	-
SpyC-GTA/R176G	EDTA	-	-	14.41 ± 0.31	-	-	-	-	-	-	-
	Mn^{2+}	-	3632 ± 122	3143 ± 42	-	2286 ± 356	1777 ± 380	1576 ± 536	1107 ± 107	2247 ± 20	2188 ± 1198
	Mg^{2+}	-	2188 ± 1198	1877 ± 616	-	1853 ± 276	1582 ± 265	1698 ± 313	1984 ± 50	1848 ± 203	2096 ± 209
	Mix	-	-	-	-	-	-	-	-	2195 ± 1134	-

Table S8 Efficiency of SpyT-coupling to maleimide-activated agarose. Applied SpyT, measured excess SpyT, the balance, and final yield are shown for each batch of SpyT agarose. Due to a more convenient processability, larger batches were divided into several approaches ranging from 1-2.5 mL. Excess SpyTag was measured via Bradford assay. All approaches were processed identically

Agarose Batch	Approach	Agarose Volume [mL]	Applied SpyTag [mg]	Measured excess SpyTag [mg]	Balance [mg]	Yield [%]
Batch 1	Approach 1	1.1	2.5	0	2.5	100
Batch 2	Approach 1	1	5	0	5	100
Batch 3	Approach 1	1	5	0	5	100
	Approach 3	1	5	0	5	100
	Approach 3	1	5	0	5	100
Batch 4	Approach 1	1	5	0.08	4.92	98.4
	Approach 2	1	5	0	5	100
Batch 5	Approach 1	1	5	0.02	4.98	99.6
Batch 6	Approach 1	2	10	0	10	100
	Approach 2	2	10	0	10	100
	Approach 3	2	10	0	10	100
Batch 7	Approach 1	2	10	0	10	100
Batch 8	Approach 1	2.5	12.5	0.1	12.4	99.2
	Approach 2	2.5	12.5	0.4	12.46	99.7
					**Overall yield:**	**99.8 ± 0.4**

Table S9 Yields for the coupling of SpyC-GTs to the SpyTag-agarose. The best result for the coupling of each SpyC-GT is shown. Excess SpyC-GT was measured via Bradford assay. All coupling procedures were handled identically

Enzyme	Amount applied		Amount coupled		
	[mg]	[µmol]	[mg]	[µmol]	Yield [%]
SpyC-β4GalT	1.54	0.021	1.1	0.015	71.4
SpyC-LgtA	1.98	0.021	1.32	0.014	66.7
SpyC-WbgO	1.49	0.022	1.22	0.018	81.8
SpyC-FutC	1.52	0.021	1.3	0.018	85.7
SpyC-GTA/R176G	1.52	0.021	1.52	0.021	100

Table S10 Comparison of the specific activity of soluble SpyC-GTs with SpyC-GTs immobilized on SpyT-agarose. Activity assays for both variants of each enzyme were performed identically

Enzyme	Activity of free SpyC-GT [mU·mg^{-1}]	Activity of coupled SpyC-GT [mU·mg^{-1}]	Activity yield [%]
SpyC-β4GalT	267.5	107.4	40.2
SpyC-LgtA	433.8	85.1	19.6
SpyC-WbgO	197.67	96.85	49.0
SpyC-FutC	15.1	3.2	21.3
SpyC-GTA/R176G	1312.1	473.9	36.1

Table S11 Long-term stability of SpyC-GT agaroses. Specific activity [mU·mg^{-1}] and relative activity [%] are shown in the dependency of storage time over a time course of one month. For relative activities, specific activities were normalized to the initial activity (100 %) on day one

Enzyme	Activity	Time		
		Day 1	One week	One month
SpyC-β4GalT	Specific [mU·mg^{-1}]	107.44 ± 0.95	141.01 ± 1.24	148.75 ± 8.22
	Relative [%]	100	131.2 ± 0.9	138.5 ± 5.5
SpyC-LgtA	Specific [mU·mg^{-1}]	92.95 ± 8.68	105.22 ± 8.32	57.29 ± 3.08
	Relative [%]	100	113.2 ± 7.9	61.6 ± 5.4
SpyC-WbgO	Specific [v]	96.85 ± 16.65	102.72 ± 8.23	69.85 ± 3.3
	Relative [%]	100	106.1 ± 8.0	72.1 ± 4.7
SpyC-FutC	Specific [mU·mg^{-1}]	1.06 ± 0.03	0.72 ± 0.14	0.6 ± 0.11
	Relative [%]	100	68.5 ± 19.2	56.9 ± 17.8
SpyC-GTA/R176G	Specific [mU·mg^{-1}]	473.98 ± 39.91	561.24 ± 19.58	611.53 ± 9.12
	Relative [%]	100	118.4 ± 3.5	129.0 ± 1.5

Table S12 Reusability of SpyC-GT agaroses. Absolute and relative product yields [%] are shown for 6 consecutive reactions over a time course of 3 days. For relative product yields, absolute product yields were normalized to the highest product yield of all 6 reactions (see Figure S6)

Enzyme	Product yield	Reaction #					
		1	2	3	4	5	6
SpyC-β4GalT	Absolute [%]	83.73 ± 1.07	82.26 ± 5.4	77.09 ± 5.81	76.64 ± 5.46	74.59 ± 2.87	73.93 ± 3.14
	Relative [%]	100 ± 1.3	98.2 ± 6.5	92.1 ± 6.9	91.5 ± 6.5	89.1 ± 3.4	88.3 ± 3.8
SpyC-LgtA	Absolute [%]	86.94 ± 1.2	77.64 ± 2.97	56.23 ± 6.95	42.17 ± 7.79	23.1 ± 6.66	17.18 ± 5.71
	Relative [%]	100 ± 1.4	89.3 ± 3.4	64.7 ± 8.0	48.5 ± 9.0	26.6 ± 7.7	19.8 ± 6.6
SpyC-WbgO	Absolute [%]	53.18 ± 0.12	65.17 ± 0.97	60.57 ± 1.49	70.05 ± 1.5	62.04 ± 0.89	70.72 ± 1.29
	Relative [%]	75.2 ± 0.2	92.2 ± 1.4	85.7 ± 2.1	99.1 ± 2.1	87.7 ± 1.3	100 ± 1.8
SpyC-FutC	Absolute [%]	1.54 ± 0.12	1.12 ± 0.08	0.5 ± 0.1	0.37 ± 0.07	0.17 ± 0.06	0.15 ± 0.04
	Relative [%]	100 ± 7.6	72.4 ± 5.5	32.6 ± 6.4	23.8 ± 3.7	11.2 ± 3.7	9.9 ± 2.4
SpyC-GTA/R176G	Absolute [%]	98.13 ± 2.64	97.37 ± 3.72	100	99.37 ± 0.89	100	98.53 ± 2.08
	Relative [%]	98.1 ± 2.4	97.4 ± 3.7	100	99.4 ± 0.9	100	98.5 ± 2.1

5. Figures

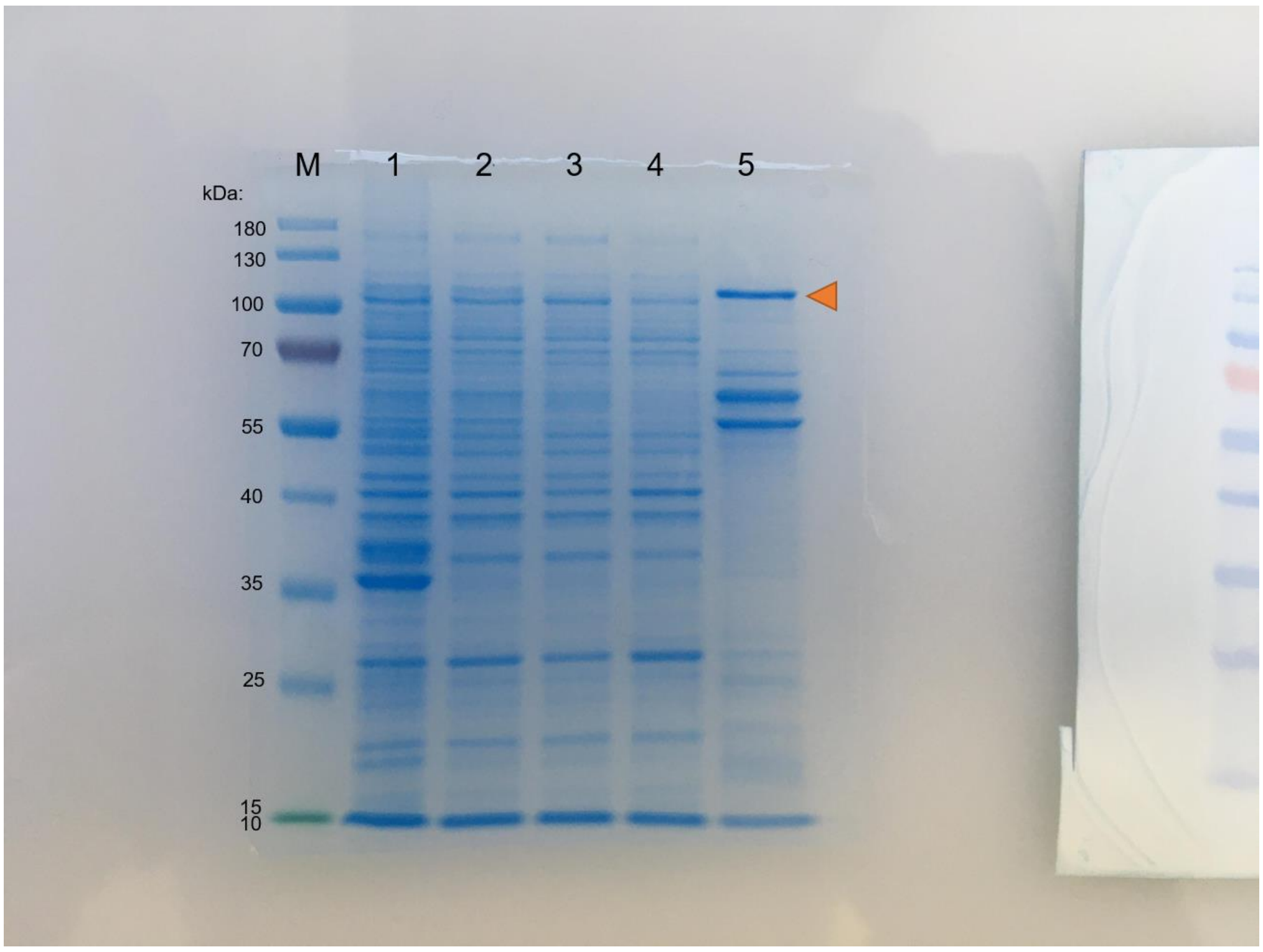

Fig. S1 SDS-PAGE of SpyC-β4GalT. The orange arrow indicates the band of the purified enzyme. Lane layout: M: Marker; 1: Pellet; 2: Raw extract; 3: IMAC Flow; 4: IMAC Wash; 5: IMAC Eluate.

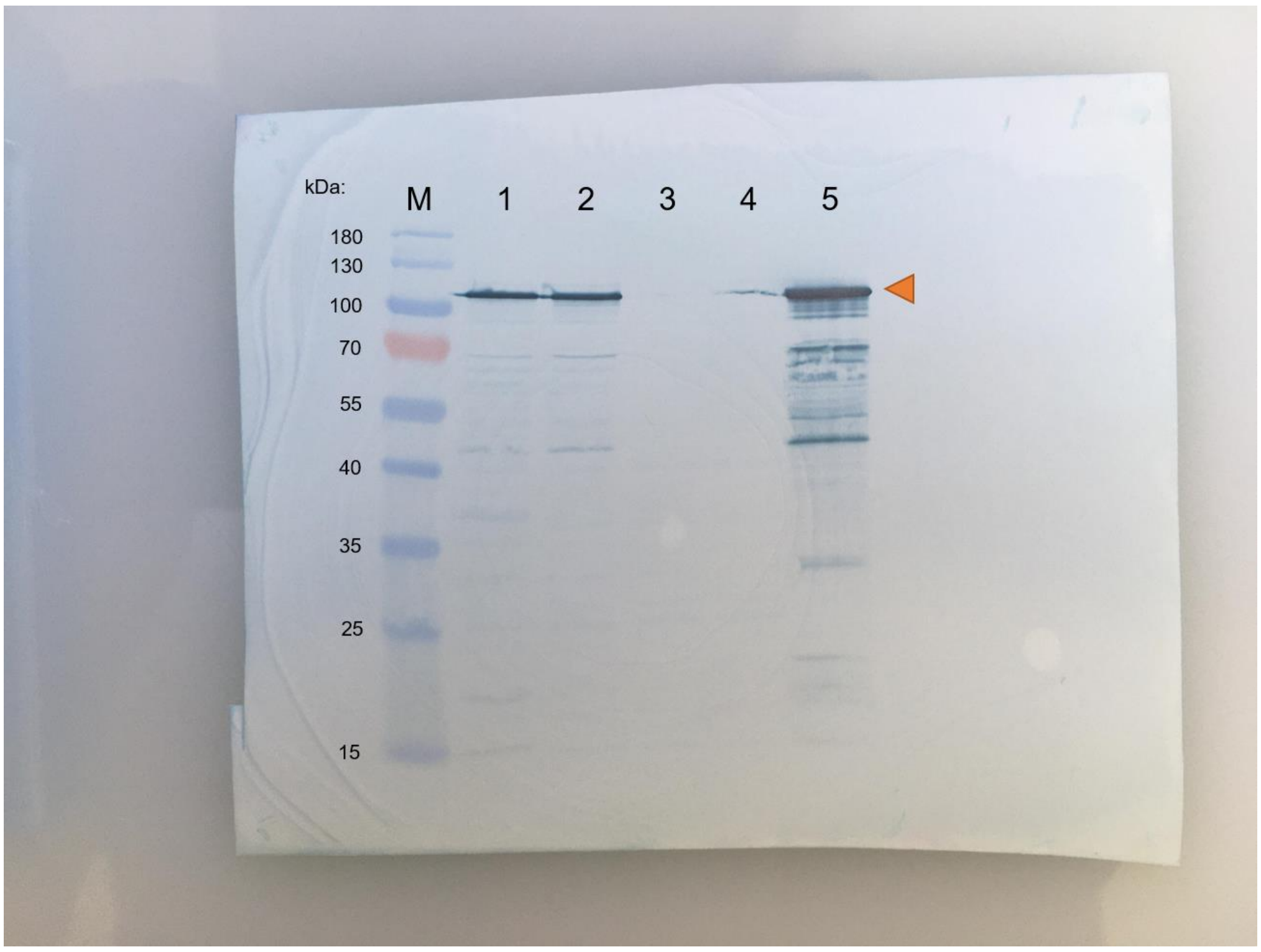

Fig. S2 Western blot of SpyC-β4GalT. The orange arrow indicates the band of the purified enzyme. Lane layout: M: Marker; 1: Pellet; 2: Raw extract; 3: IMAC Flow; 4: IMAC Wash; 5: IMAC Eluate.

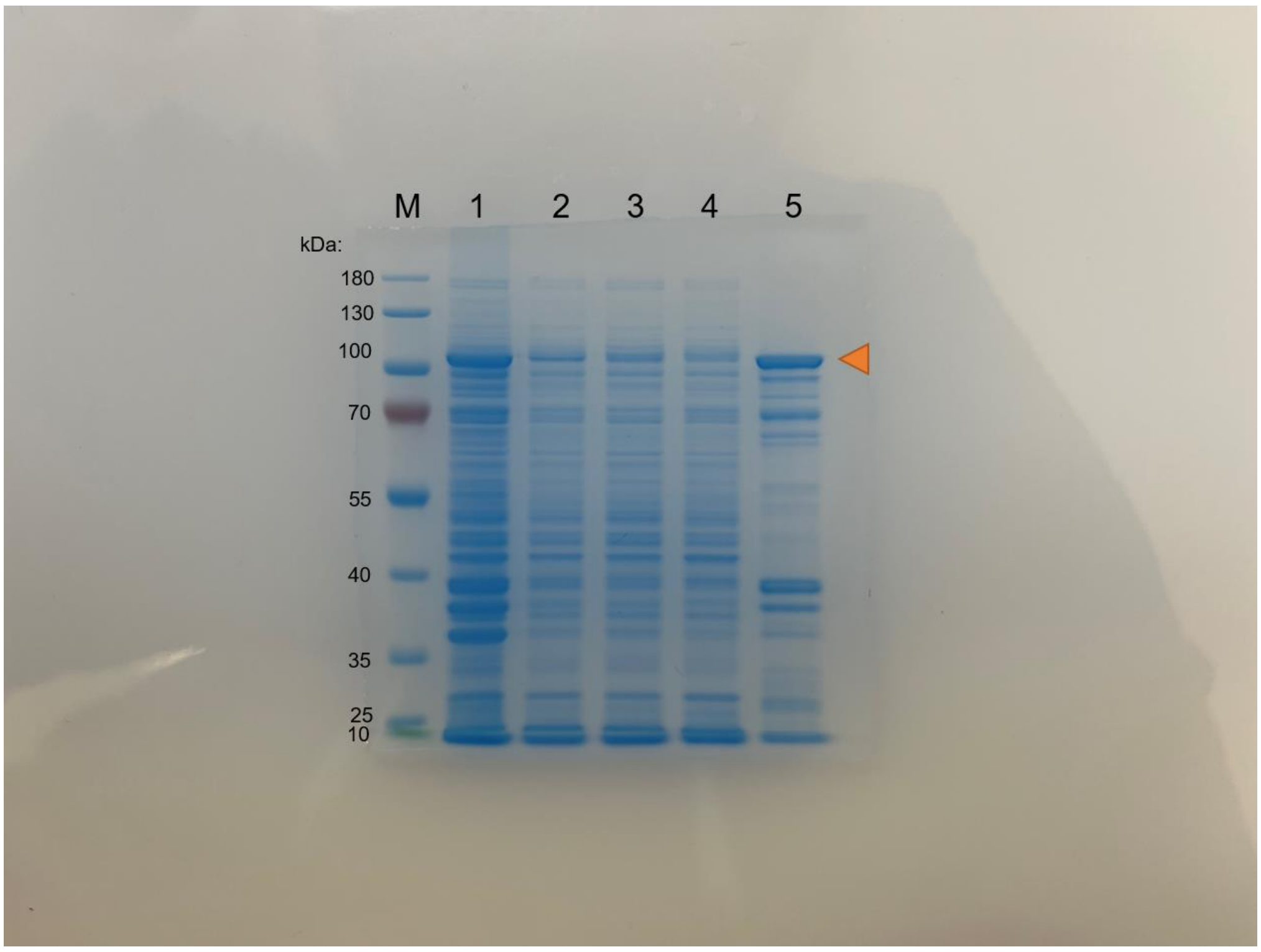

Fig. S3 SDS-PAGE of SpyC-LgtA. The orange arrow indicates the band of the purified enzyme. Lane layout: M: Marker; 1: Pellet; 2: Raw extract; 3: IMAC Flow; 4: IMAC Wash; 5: IMAC Eluate.

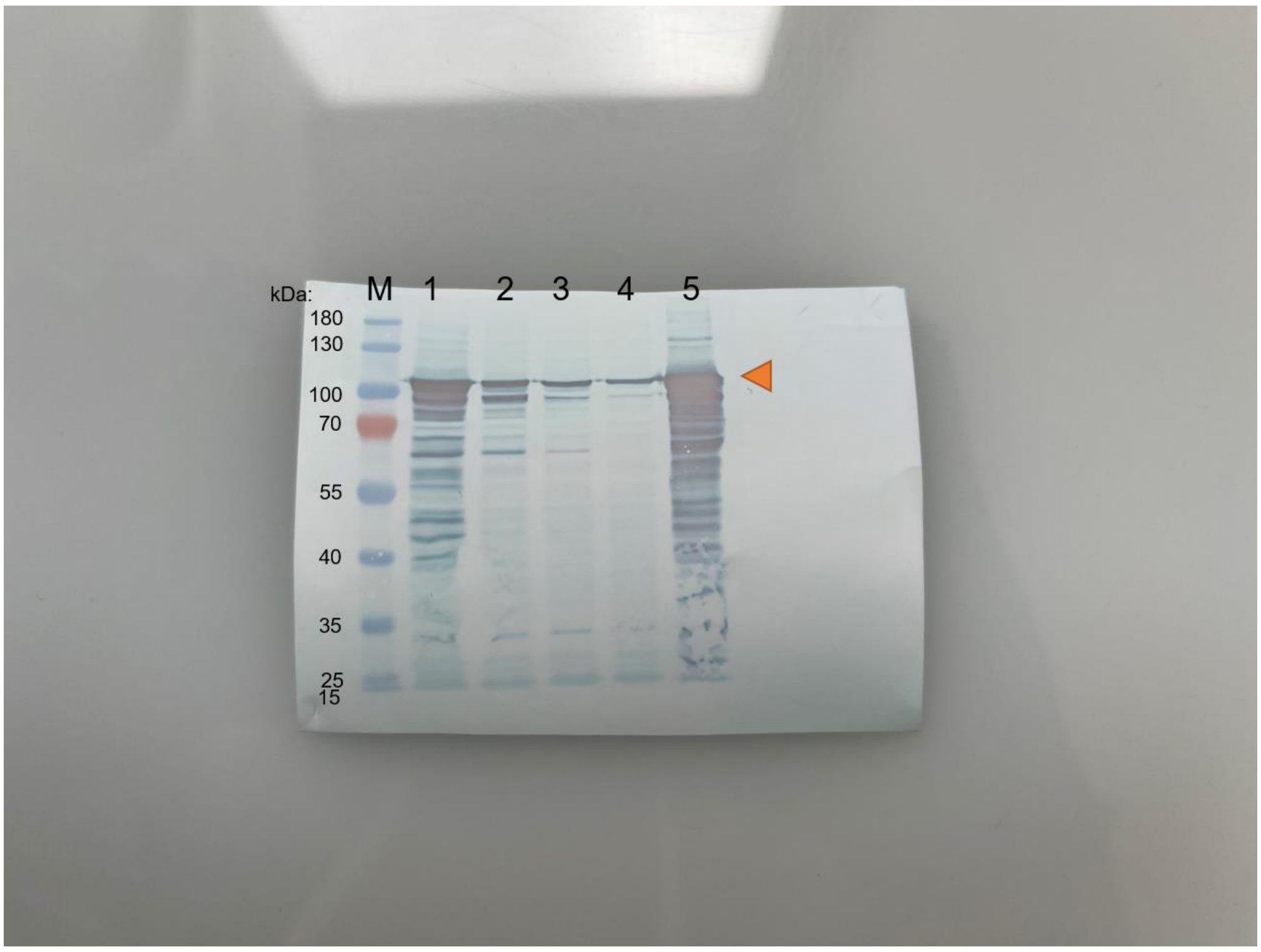

Fig. S4 Western blot of SpyC-LgtA. The orange arrow indicates the band of the purified enzyme. Lane layout: M: Marker; 1: Pellet; 2: Raw extract; 3: IMAC Flow; 4: IMAC Wash; 5: IMAC Eluate.

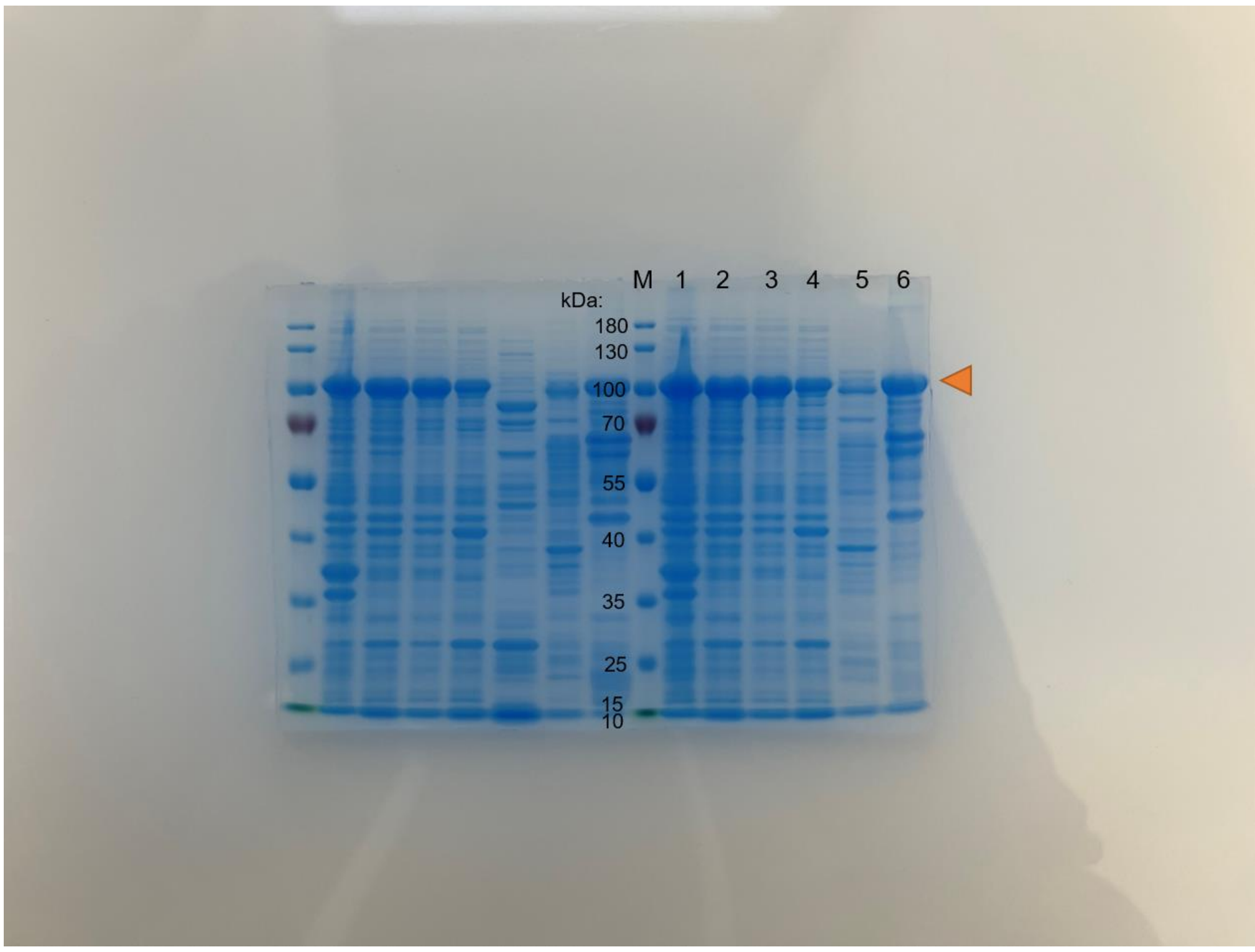

Fig. S5 SDS-PAGE of SpyC-WbgO. The orange arrow indicates the band of the purified enzyme. Lane layout: M: Marker; 1: Pellet; 2: Raw extract; 3: IMAC Flow; 4: IMAC 1. Wash; 5: IMAC 2. Wash; 6: IMAC Eluate.

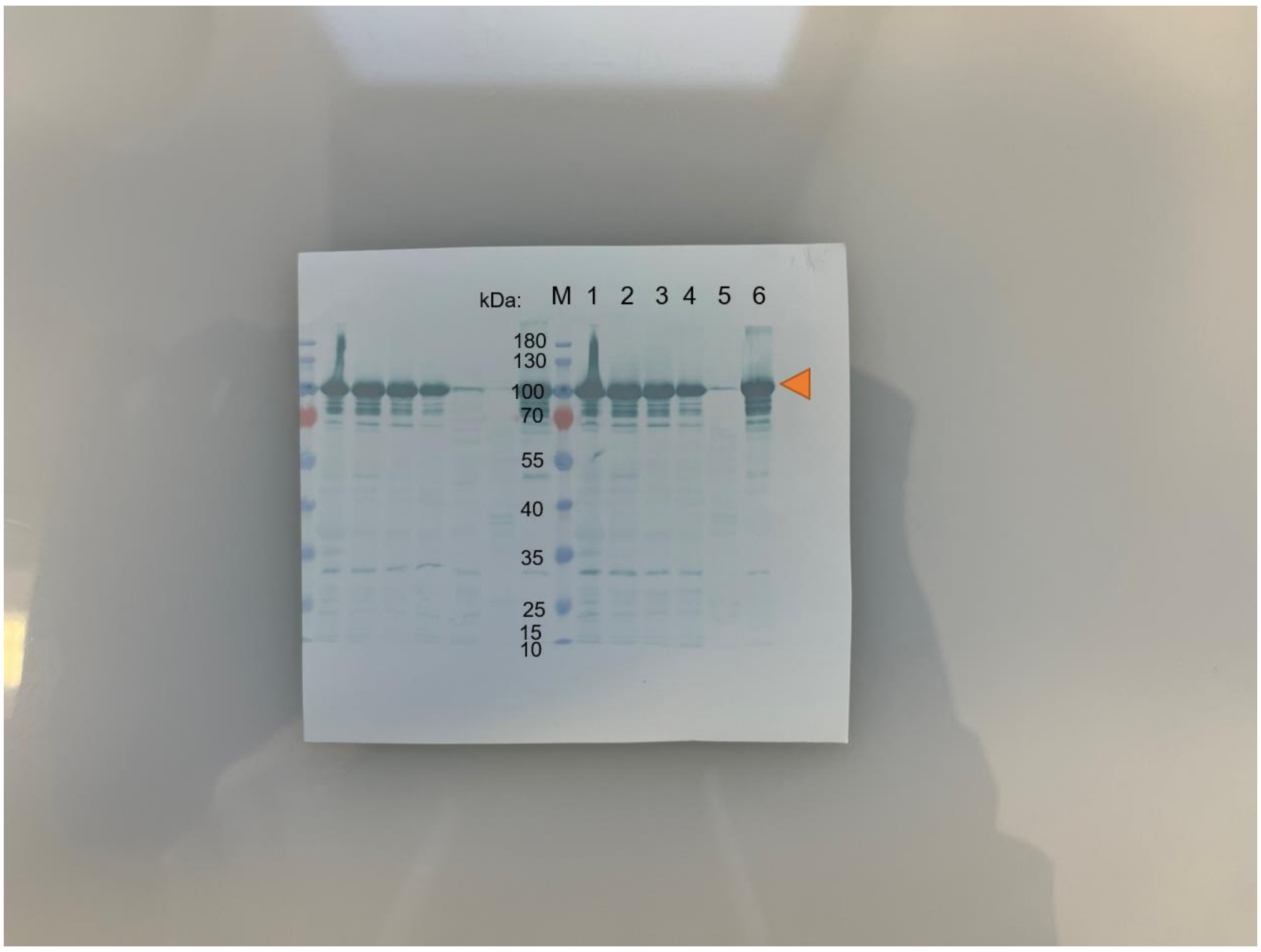

Fig. S6 Western blot of SpyC-WbgO. The orange arrow indicates the band of the purified enzyme. Lane layout: M: Marker; 1: Pellet; 2: Raw extract; 3: IMAC Flow; 4: IMAC 1. Wash; 5: IMAC 2. Wash; 6: IMAC Eluate.

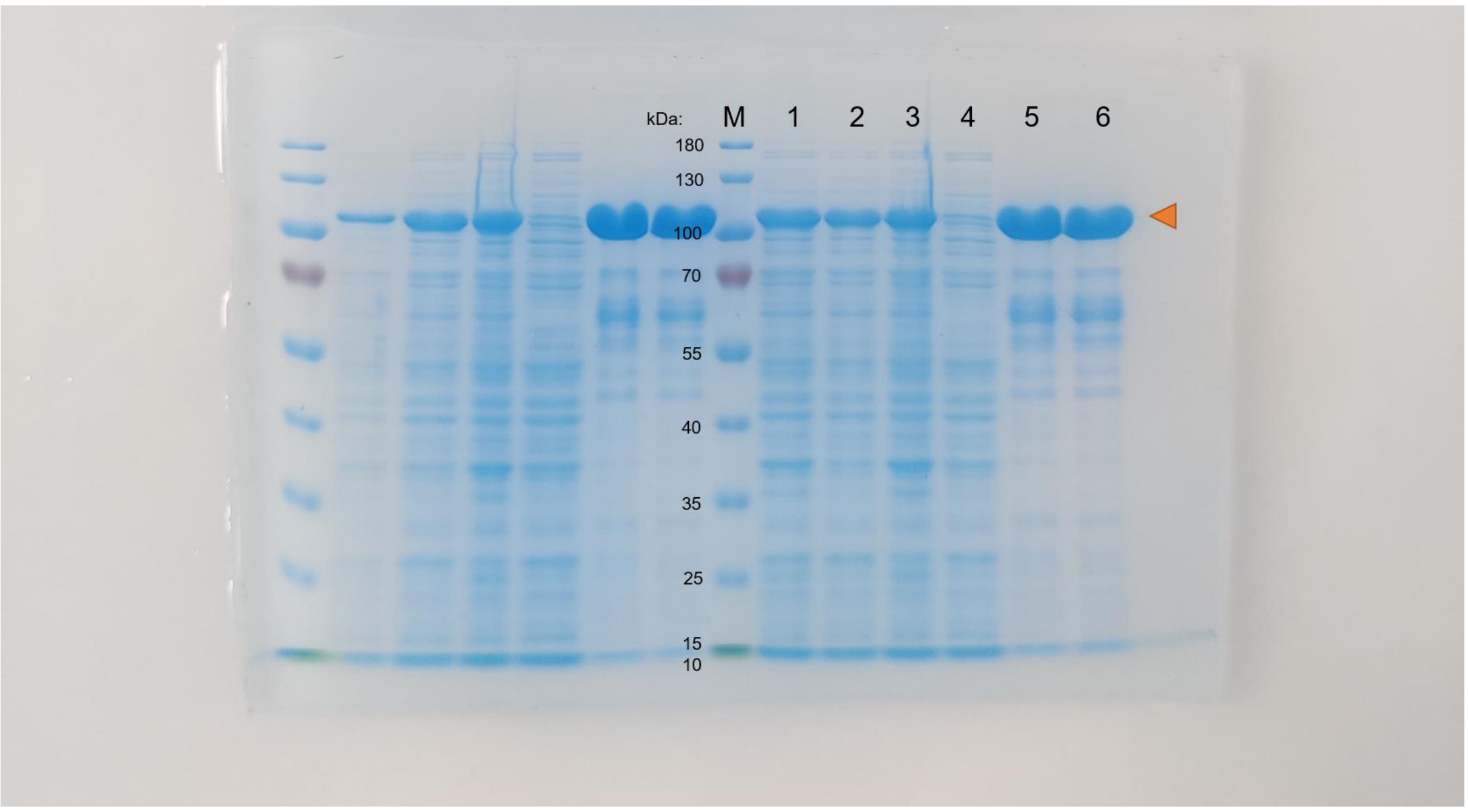

Fig. S7 SDS-PAGE of SpyC-FutC. The orange arrow indicates the band of the purified enzyme. M: Marker; 1: Raw extract; 2: Centrifuged raw extract; 3: Pellet; 4: IMAC Flow; 5: IMAC Eluate; 6: Dialysed Eluate.

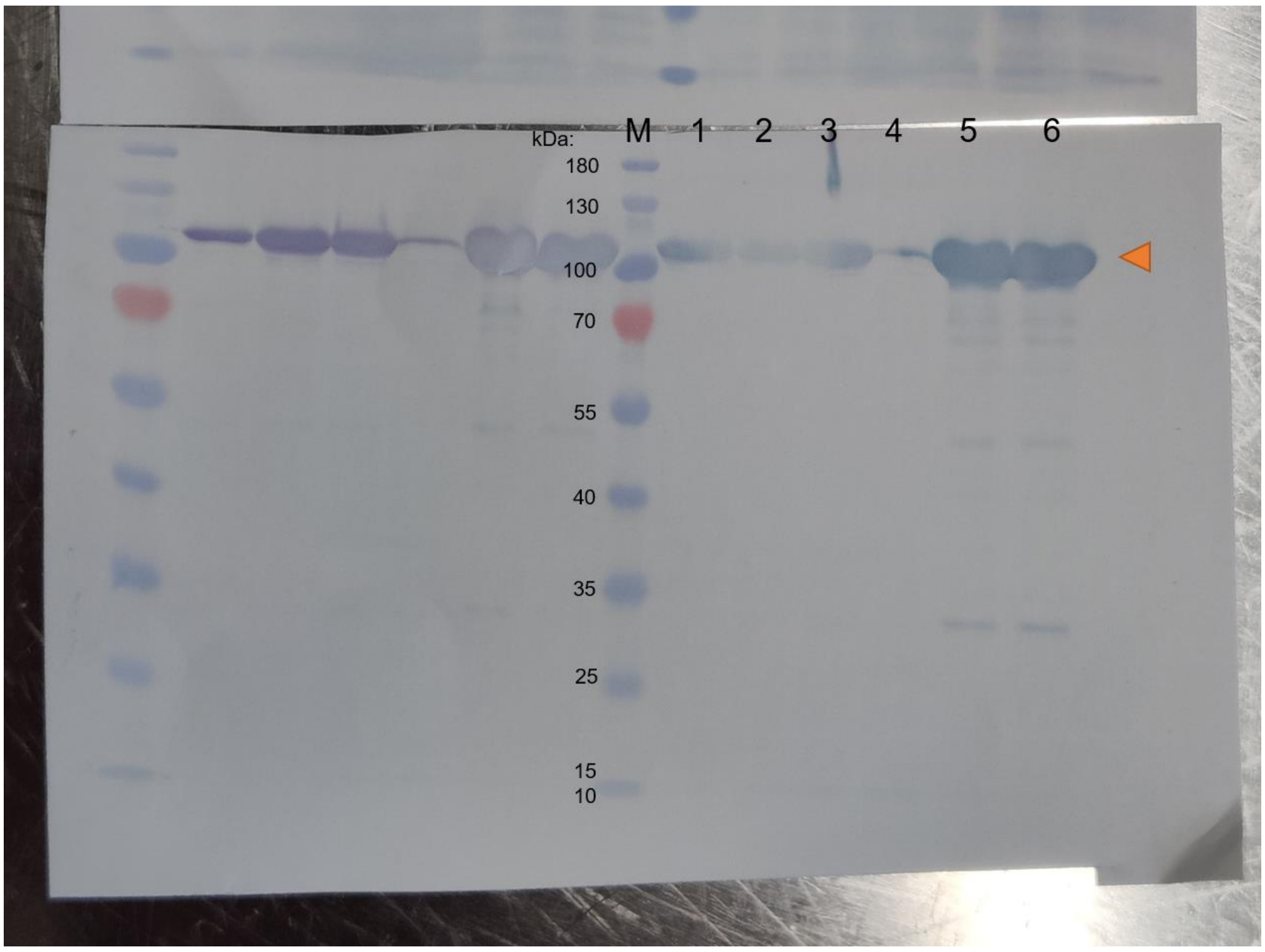

Fig. S8 Western blot of SpyC-FutC. The orange arrow indicates the band of the purified enzyme. M: Marker; 1: Raw extract; 2: Centrifuged raw extract; 3: Pellet; 4: IMAC Flow; 5: IMAC Eluate; 6: Dialysed Eluate.

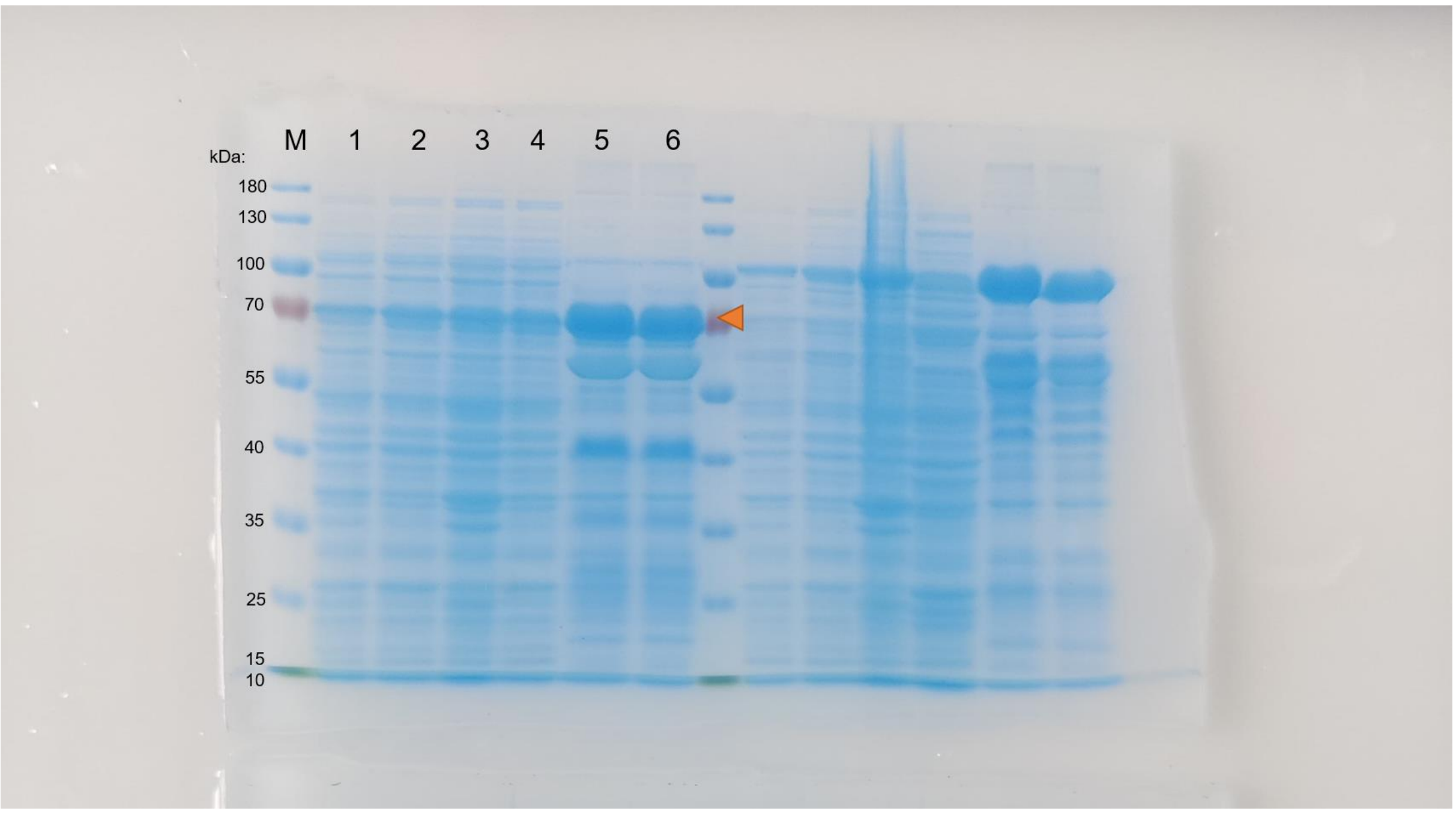

Fig. S9 SDS-PAGE of SpyC-GTA/R176G. The orange arrow indicates the band of the purified enzyme. M: Marker; 1: Raw extract; 2: Centrifuged raw extract; 3: Pellet; 4: IMAC Flow; 5: IMAC Eluate; 6: Dialysed Eluate.

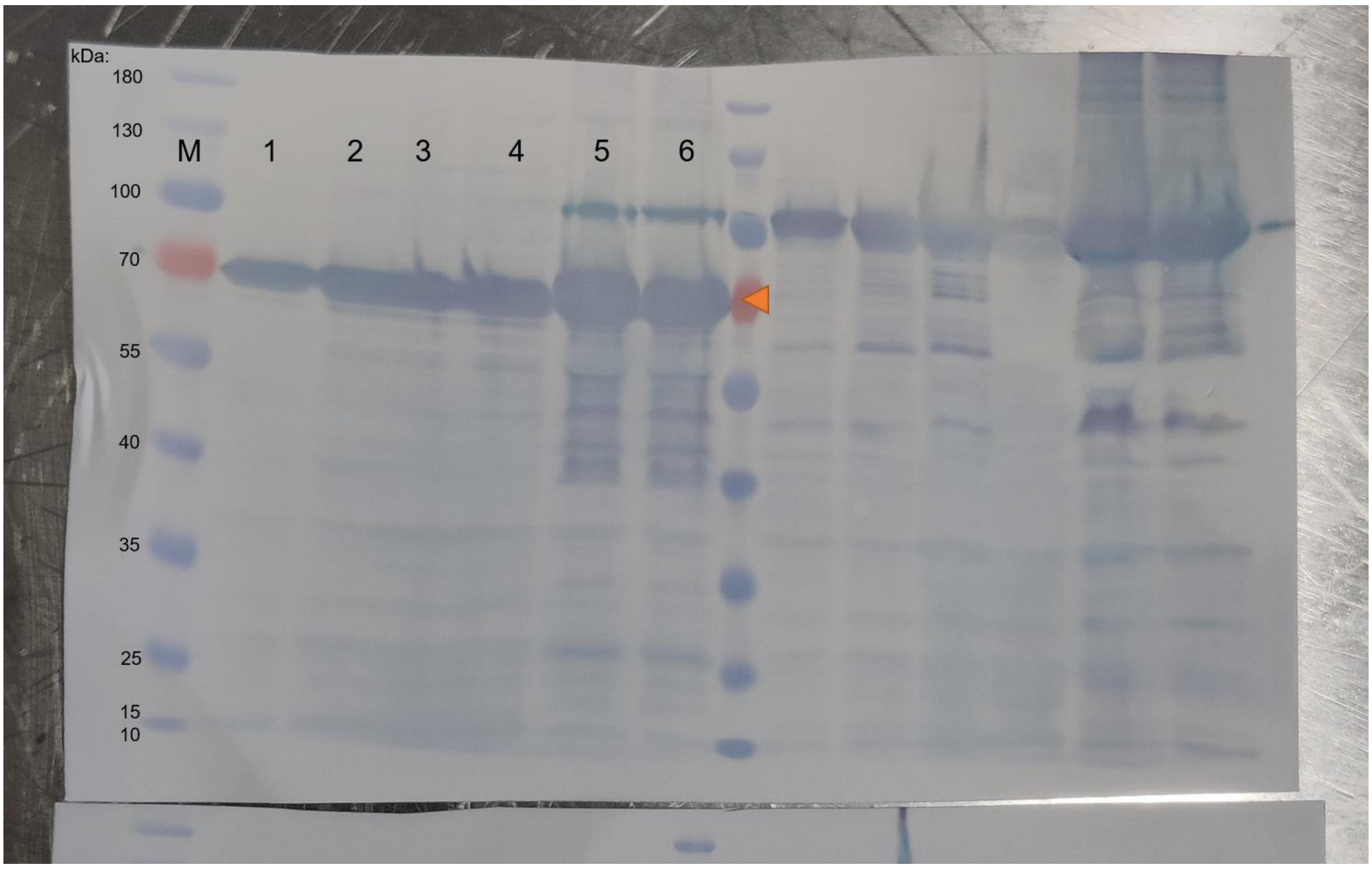

Fig. S10 SDS-PAGE of SpyC-GTA/R176G. The orange arrow indicates the band of the purified enzyme. M: Marker; 1: Raw extract; 2: Centrifuged raw extract; 3: Pellet; 4: IMAC Flow; 5: IMAC Eluate; 6: Dialysed Eluate.

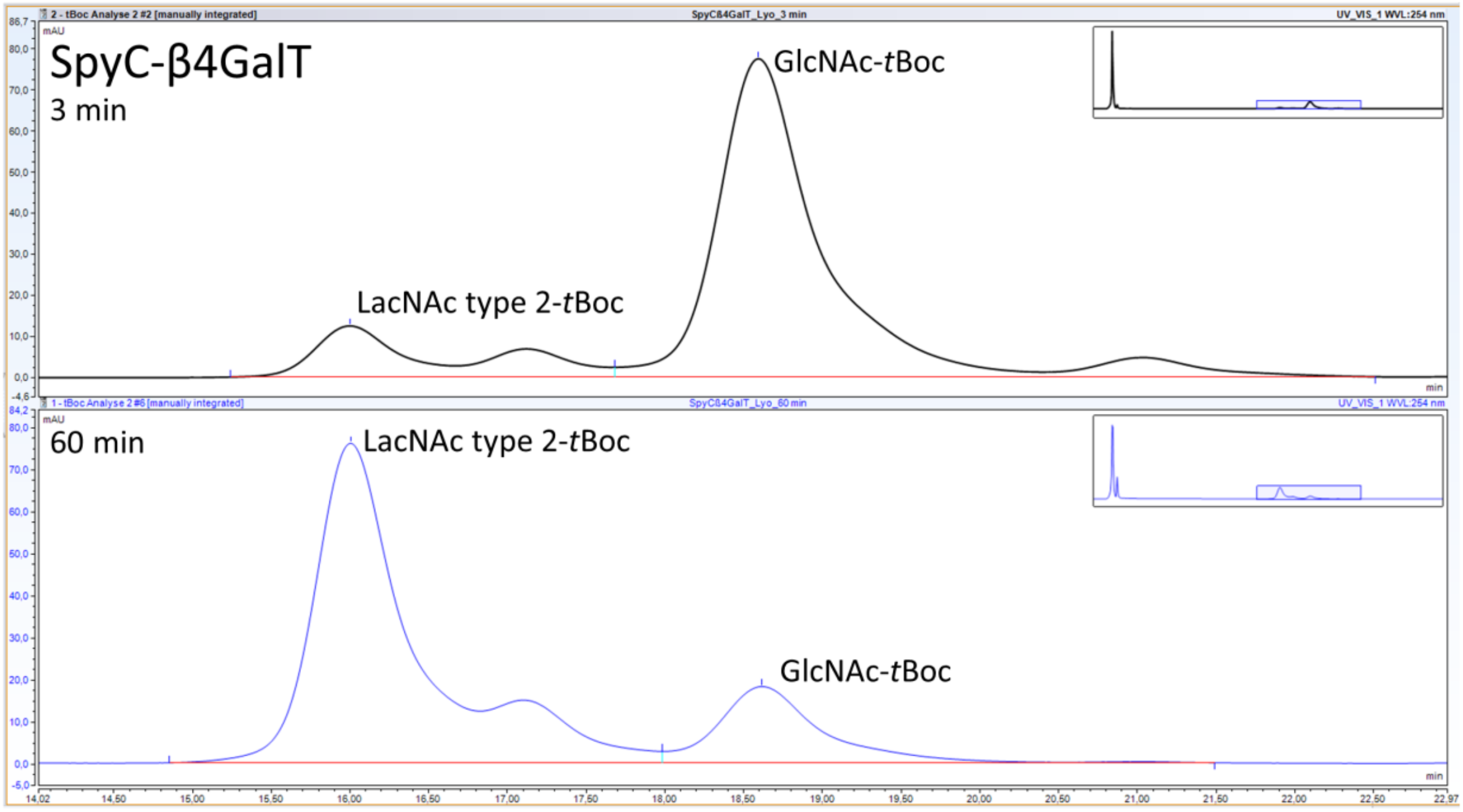

Fig. S11 HPLC chromatograms of SpyC-β4GalT activity assay. The acceptor GlcNAc-*t*Boc is elongated to LacNAc type 2-*t*Boc by the addition of galactose

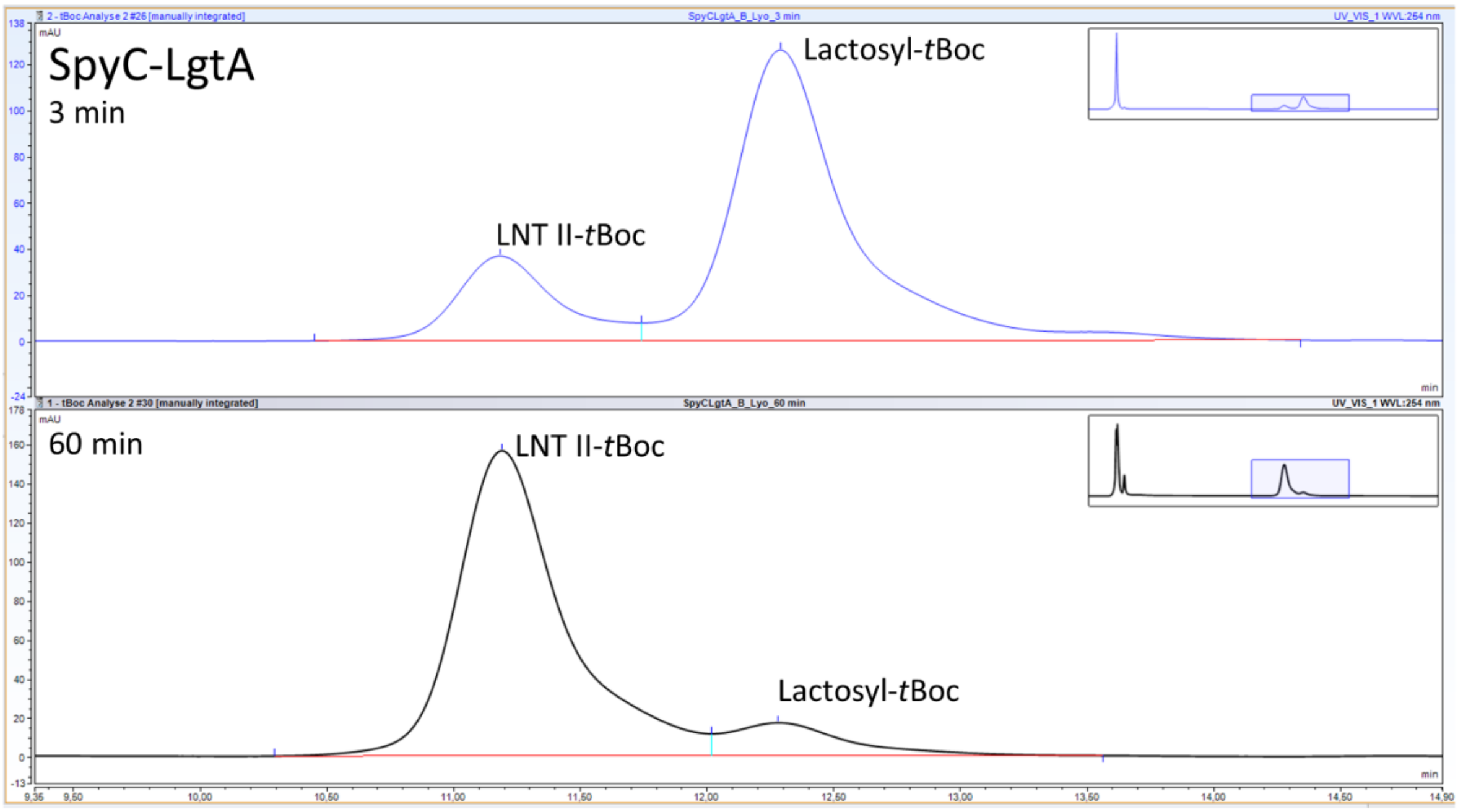

Fig. S12 HPLC chromatograms of SpyC-LgtA activity assay. The acceptor Lactosyl-*t*Boc is elongated to LNT II-*t*Boc by the addition of GlcNAc

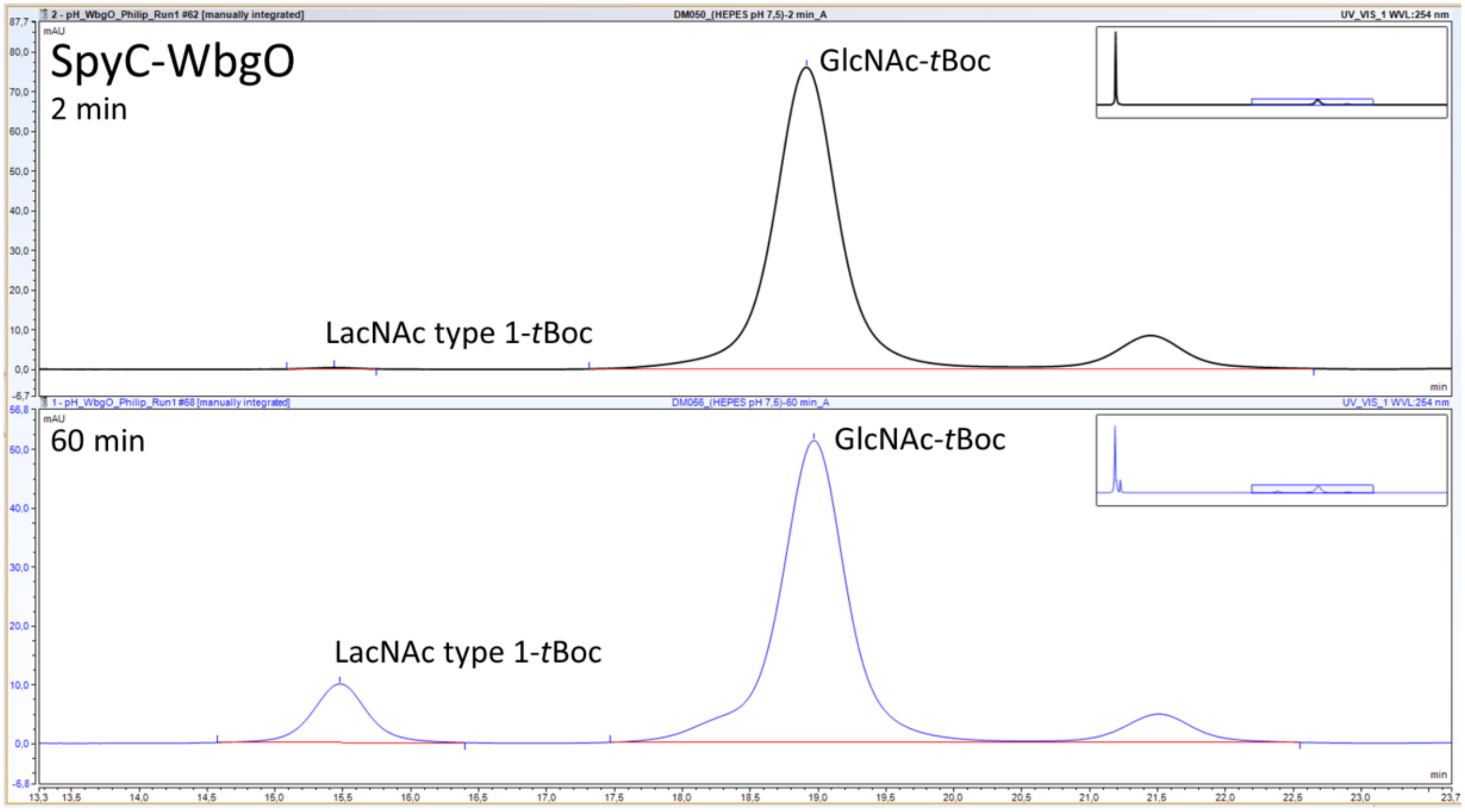

Fig. S13 HPLC chromatograms of SpyC-WbgO activity assay. The acceptor GlcNAc-*t*Boc is elongated to LacNAc type 1-*t*Boc by the addition of galactose

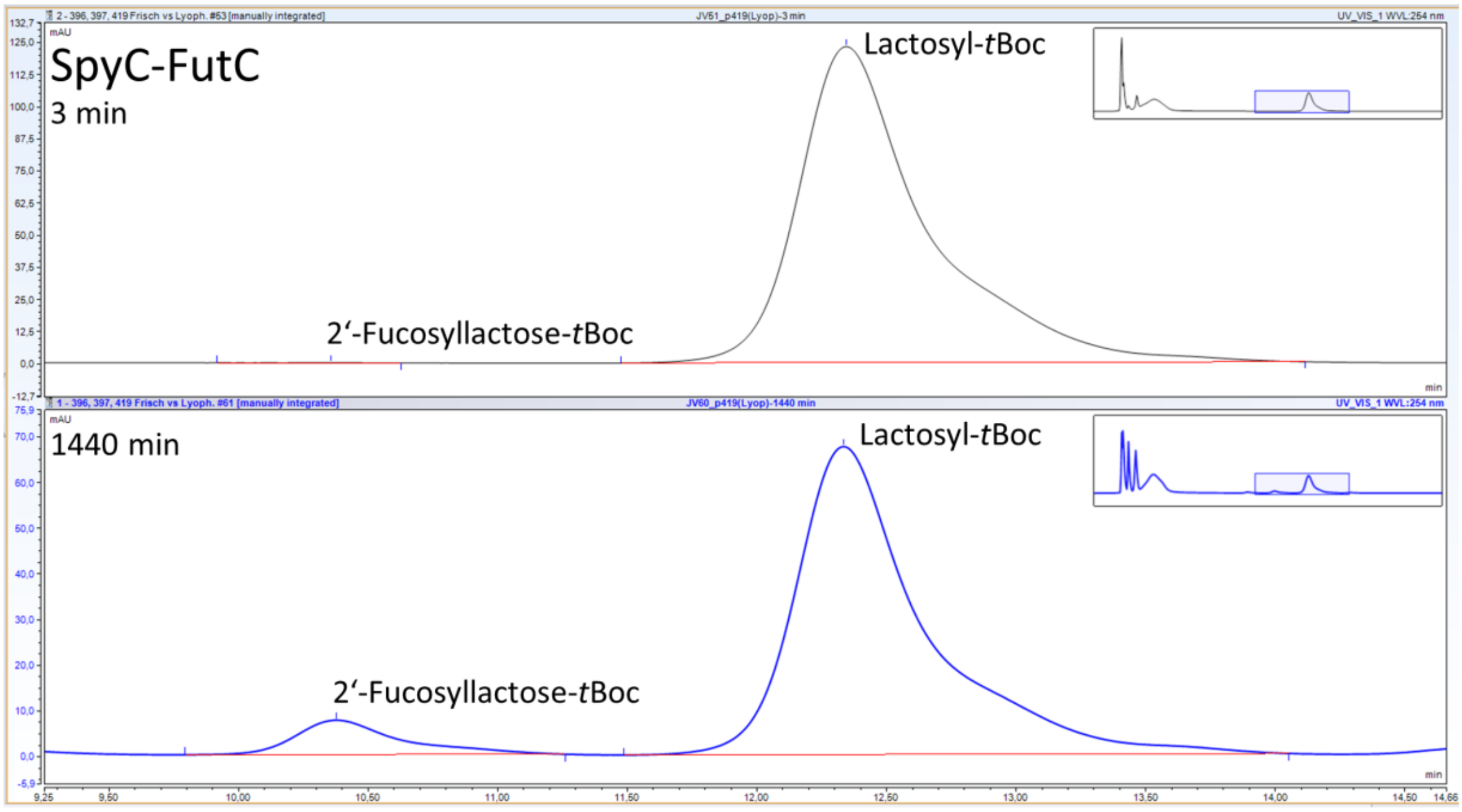

Fig. S14 HPLC chromatograms of SpyC-FutC activity assay. The acceptor Lactosyl-*t*Boc is elongated to 2'-FL-*t*Boc by the addition of fucose

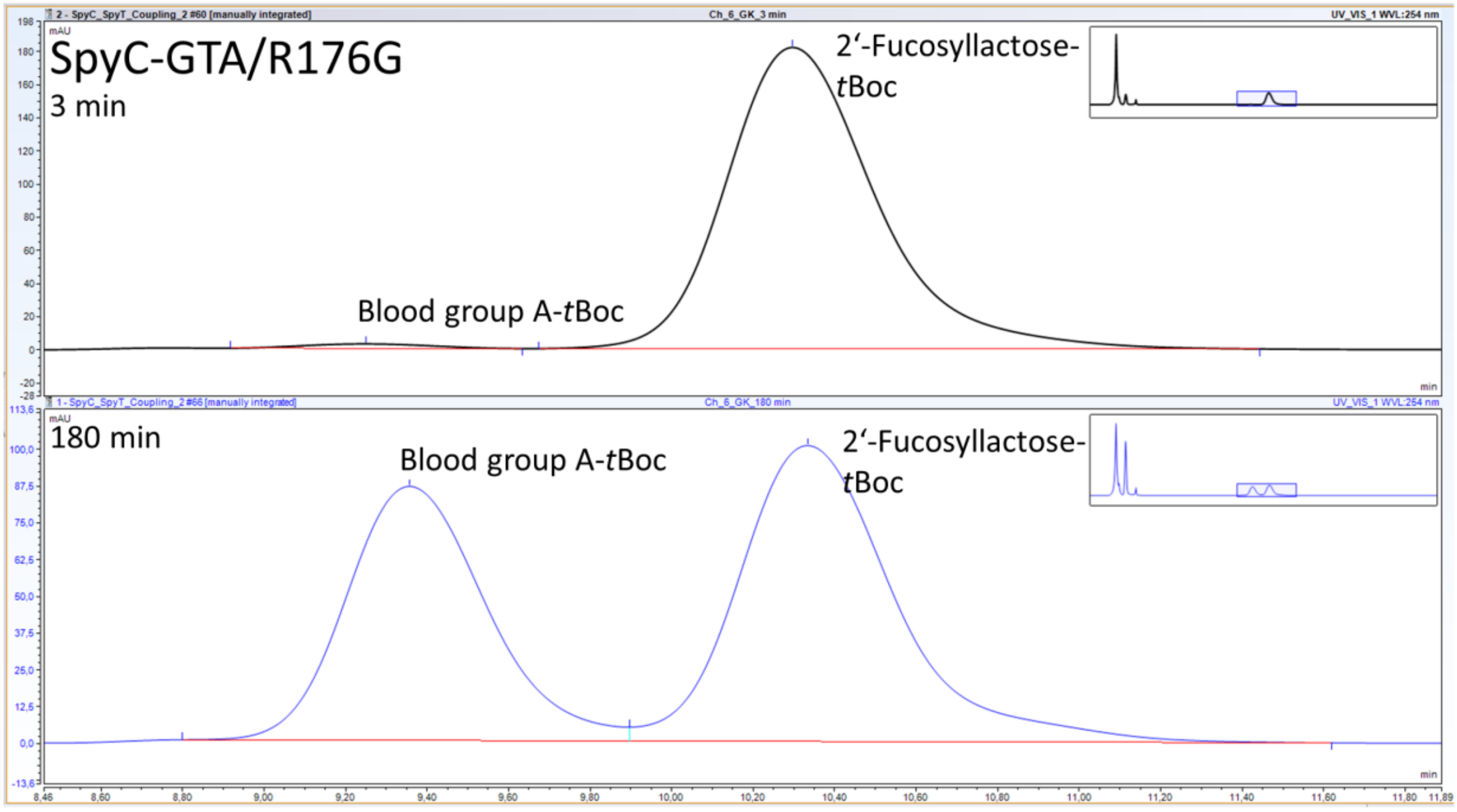

Fig. S15 HPLC chromatograms of SpyC-GTA/R176G activity assay. The acceptor 2'-FL-*t*Boc is elongated to blood group A-*t*Boc by the addition of GalNAc

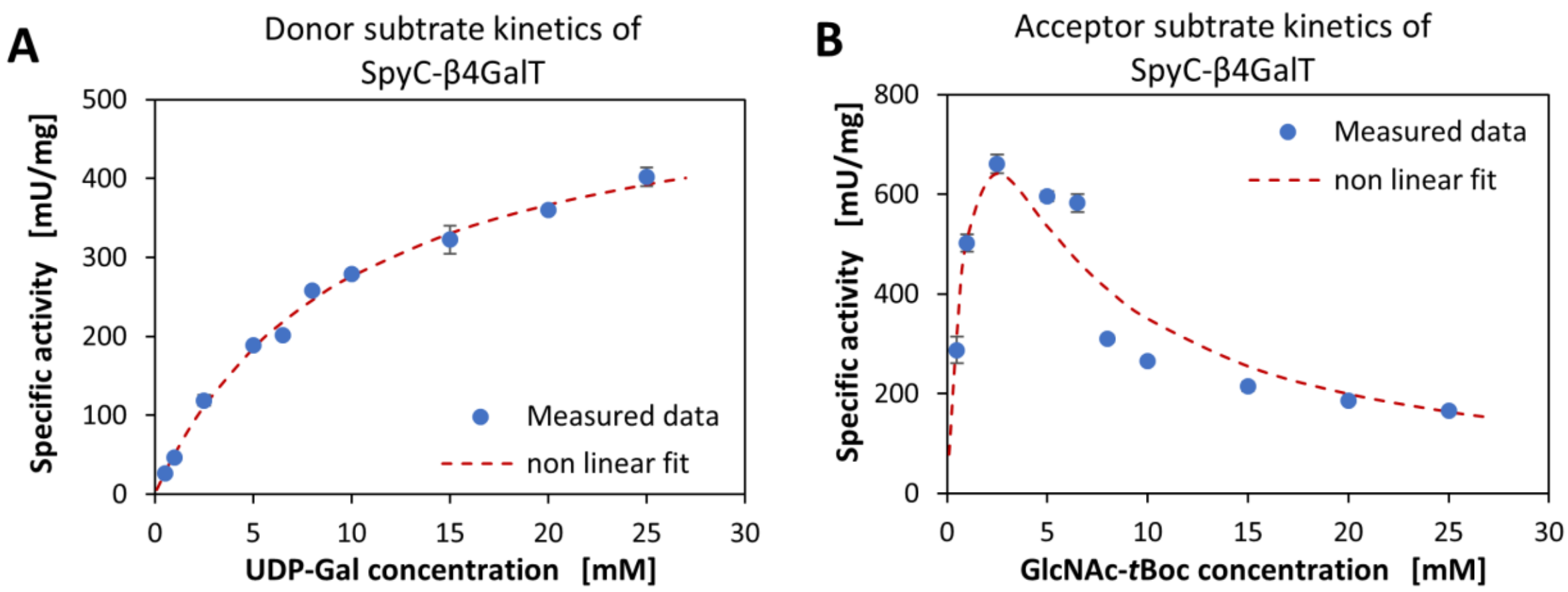

Fig. S16 Kinetic parameters for SpyC-β4GalT. **A)** Reactions were carried out at 30 °C in Glycine-NaOH pH 10, 5 mM $MnCl_2$, 5 mM GlcNAc-*t*Boc (**1**, Fig. 1B), 5 U FastAP, and 0.4 µg/µL SpyC-β4GalT with varying UDP-Gal concentrations (0.5, 1, 2.5, 5, 6.5, 8, 10, 15, 20, 25 mM). K_M: 9.79 mM, v_{max}: 546.48 mU·mg^{-1}, k_{cat}: 1.66 s^{-1}, $k_{cat}\cdot K_M^{-1}$: 0.17 mM$^{-1}\cdot$s^{-1}. **B)** Reactions were carried out at 30 °C in Glycine-NaOH pH 10, 5 mM $MnCl_2$, 5 mM UDP-Gal, 5 U FastAP, and 0.4 µg/µL SpyC-β4GalT with varying GlcNAc-*t*Boc (**1**) concentrations (0.5, 1, 2.5, 5, 6.5, 8, 10, 15, 20, 25 mM). k_M: 2.34 mM, v_{max}: 1925.51 mU·mg^{-1}, k_i: 2.34; $[S]_{opt}$: 2.34, k_{cat}: 5.86 s^{-1}, $k_{cat}\cdot K_M^{-1}$: 2.5 mM$^{-1}\cdot$s^{-1}

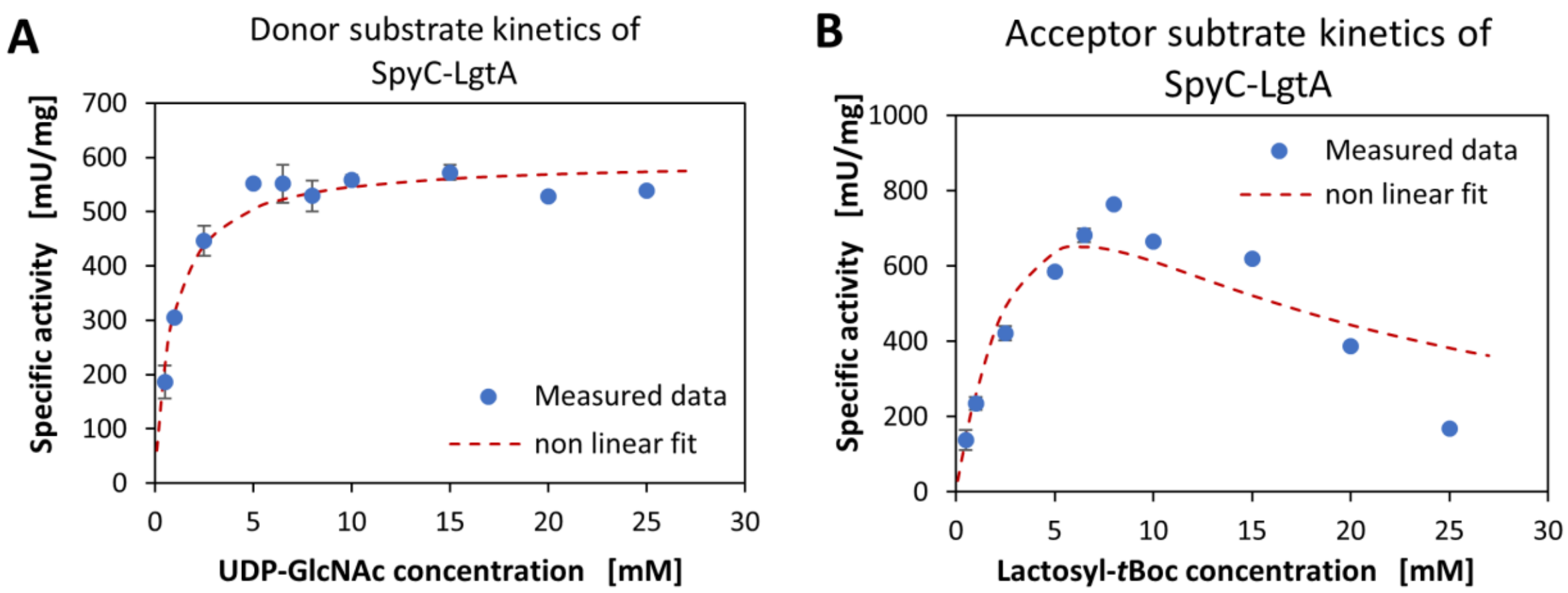

Fig. S17 Kinetic parameters for SpyC-LgtA. **A)** Reactions were carried out at 30 °C in Glycine-NaOH pH 10, 5 mM $MnCl_2$, 5 mM Lactosyl-*t*Boc (**6**, Fig. 1B), 3 U FastAP, and 0.2 µg/µL SpyC-LgtA with varying UDP-GlcNAc concentrations (0.5, 1, 2.5, 5, 6.5, 8, 10, 15, 20, 25 mM). K_M: 0.89 mM, v_{max}: 594.15 mU·mg^{-1}, k_{cat}: 9.34 s^{-1}, $k_{cat}\cdot K_M^{-1}$: 10.52 mM$^{-1}\cdot$s^{-1}. **B)** Reactions were carried out at 30 °C in Glycine-NaOH pH 10, 5 mM $MnCl_2$, 5 mM UDP-GlcNAc, 3 U FastAP, and 0.2 µg/µL SpyC-LgtA with varying Lactosyl-*t*Boc (**6**) concentrations (0.5, 1, 2.5, 5, 6.5, 8, 10, 15, 20, 25 mM). k_M: 6.5 mM, v_{max}: 1953.75 mU·mg^{-1}, k_i: 6.5, $[S]_{opt}$: 6.5, k_{cat}: 30.71 s^{-1}, $k_{cat}\cdot K_M^{-1}$: 4.72 mM$^{-1}\cdot$s^{-1}

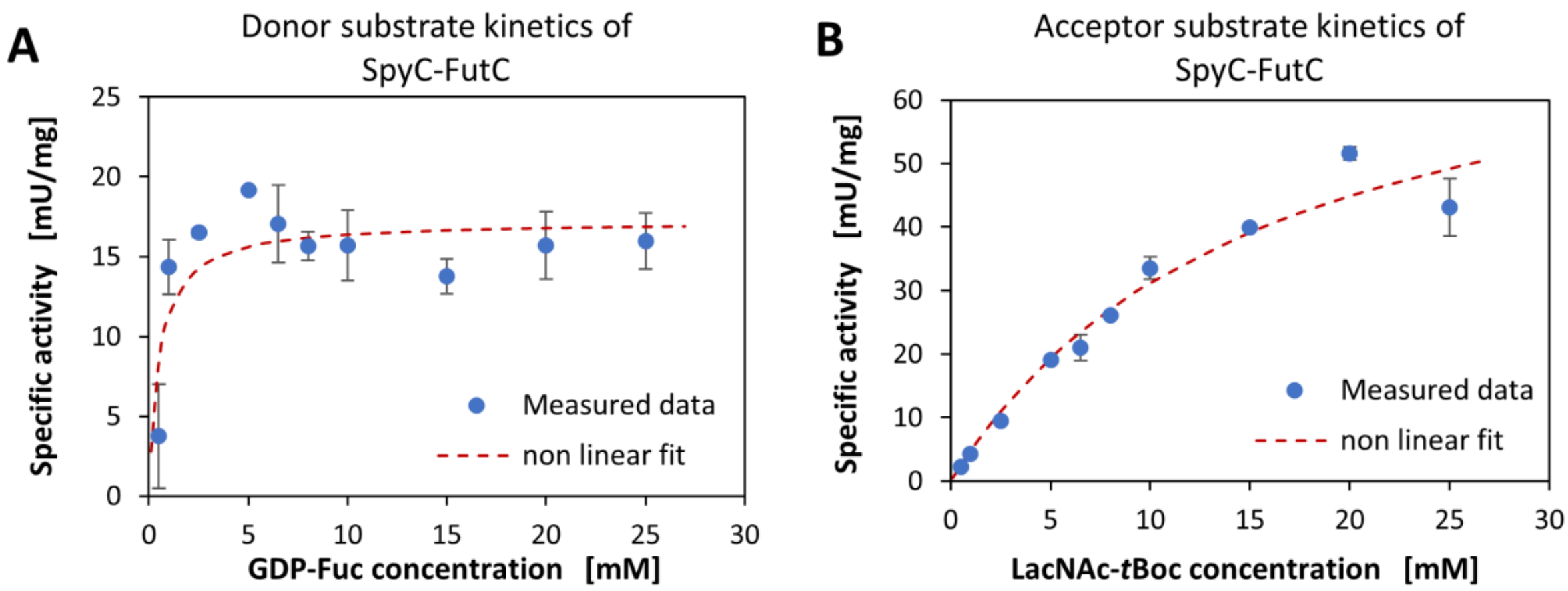

Fig. S18 Kinetic parameters for SpyC-FutC. **A)** Reactions were carried out at 30 °C in TRIS-HCl pH 6, 25 mM KCl, 5 mM $MnCl_2$, 5 mM $MgCl_2$, 5 mM LacNAc-*t*Boc (**3**, Fig. 1B), 1 U FastAP, and 0.5 µg/µL SpyC-FutC with varying GDP-Fuc concentrations (0.5, 1, 2.5, 5, 6.5, 8, 10, 15, 20, 25 mM). K_M: 0.52 mM, v_{max}: 17.2 mU·mg^{-1}, k_{cat}: 0.06 s^{-1}, k_{cat}·$K_M$$^{-1}$: 0.12 mM^{-1}·s^{-1}. **B)** Reactions were carried out at 30 °C in TRIS-HCl pH 6, 25 mM KCl, 5 mM $MnCl_2$, 5 mM $MgCl_2$, 5 mM GDP-Fuc, 1 U FastAP, and 0.5 µg/µL SpyC-FutC with varying LacNAc-*t*Boc (**3**) concentrations (0.5, 1, 2.5, 5, 6.5, 8, 10, 15, 20, 25 mM). K_M: 15.66 mM, v_{max}: 79.98 mU·mg^{-1}, k_{cat}: 0.29 s^{-1}, k_{cat}·$K_M$$^{-1}$: 0.02 mM^{-1}·s^{-1}

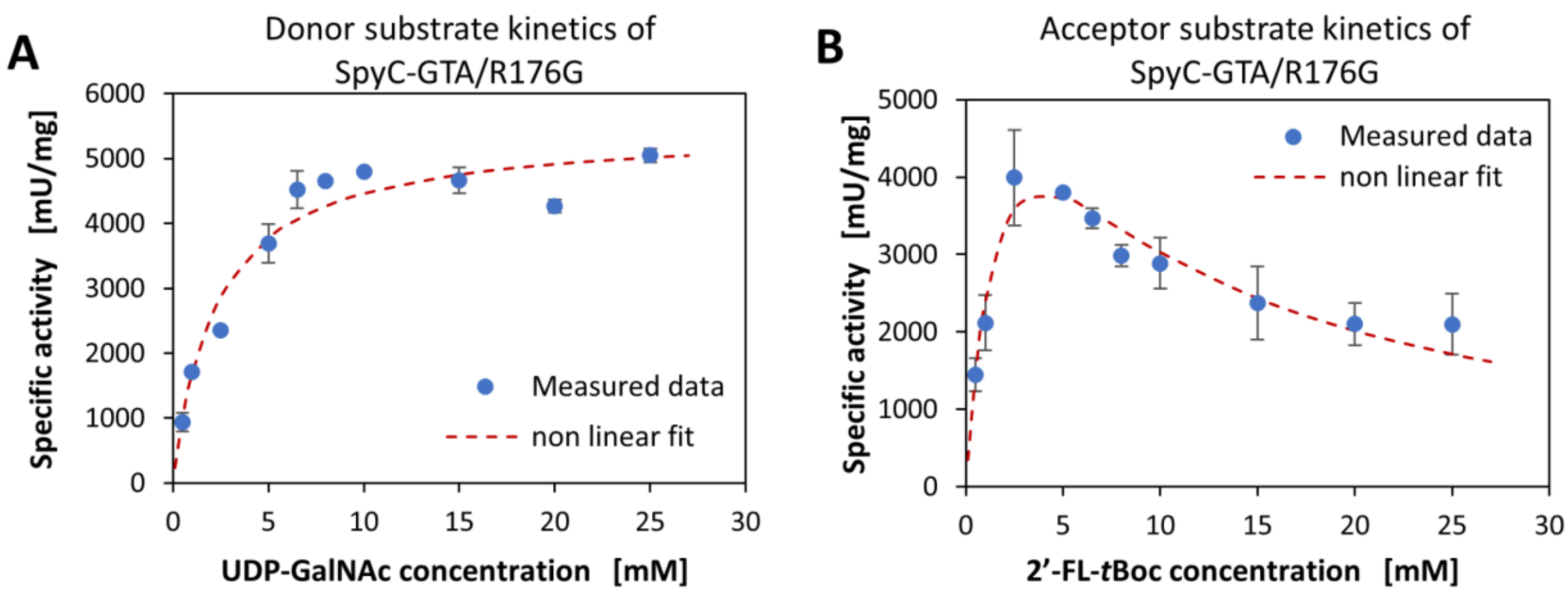

Fig. S19 Kinetic parameters for SpyC-GTA/R176G. **A)** Reactions were carried out at 30 °C in MOPS-NaOH pH 7, 20 mM $MgCl_2$, 5 mM 2'-FL-*t*Boc (**7**, Fig. 1B), 1 U FastAP, 1 mg/mL BSA, and 0.01 µg/µL SpyC-GTA/R176G with varying UDP-GalNAc concentrations (0.5, 1, 2.5, 5, 6.5, 8, 10, 15, 20, 25 mM). K_M: 2.26 mM, v_{max}: 5462.72 mU·mg^{-1}, k_{cat}: 62.36 s^{-1}, k_{cat}·$K_M$$^{-1}$: 27.59 mM^{-1}·s^{-1}. **B)** Reactions were carried out at 30 °C in MOPS-NaOH pH 7, 20 mM $MgCl_2$, 5 mM UDP-GalNAc, 1 U FastAP, 1 mg/mL BSA, and 0.01 µg/µL SpyC-GTA/R176G with varying 2'-FL-*t*Boc (**7**) concentrations (0.5, 1, 2.5, 5, 6.5, 8, 10, 15, 20, 25 mM). K_M: 2.28 mM, v_{max}: 8179.04 mU·mg^{-1}, k_i: 6.77, $[S]_{opt}$: 3.93, k_{cat}: 93.36 s^{-1}, k_{cat}·$K_M$$^{-1}$: 41.01 mM^{-1} ·s^{-1}

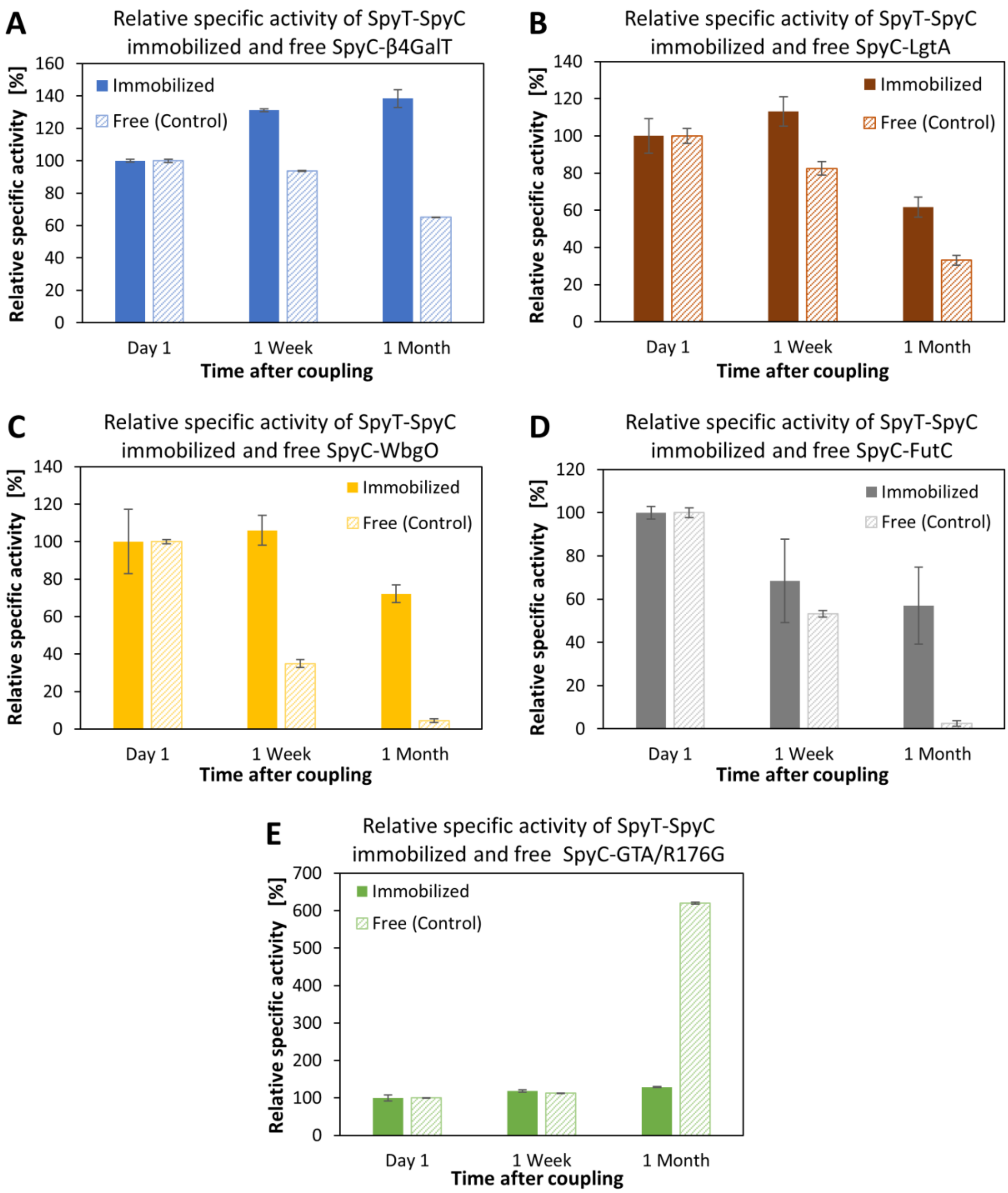

Fig. S20 Comparison of the storage stability of immobilized and free SpyC-GTs. Specific activity was measured over a time course of 1 month with storage of enzymes at 4 °C. Results were normalized to 100 % on day 1. Values are in the following denoted according to the scheme "SpyC-GT", "comparison day 1", "comparison 1 week", "comparison 1 month". **A)** SpyC-β, 100/100, 131/94, 138/65; **B)** SpyC-LgtA, 100/100, 113/82, 62/33; **C)** SpyC-WbgO, 100/100, 106/35; **D)** SpyC-FutC, 100/100, 69/53, 57/3; **E)** SpyC-GTA/R176G, 100/100, 118/113, 129/620

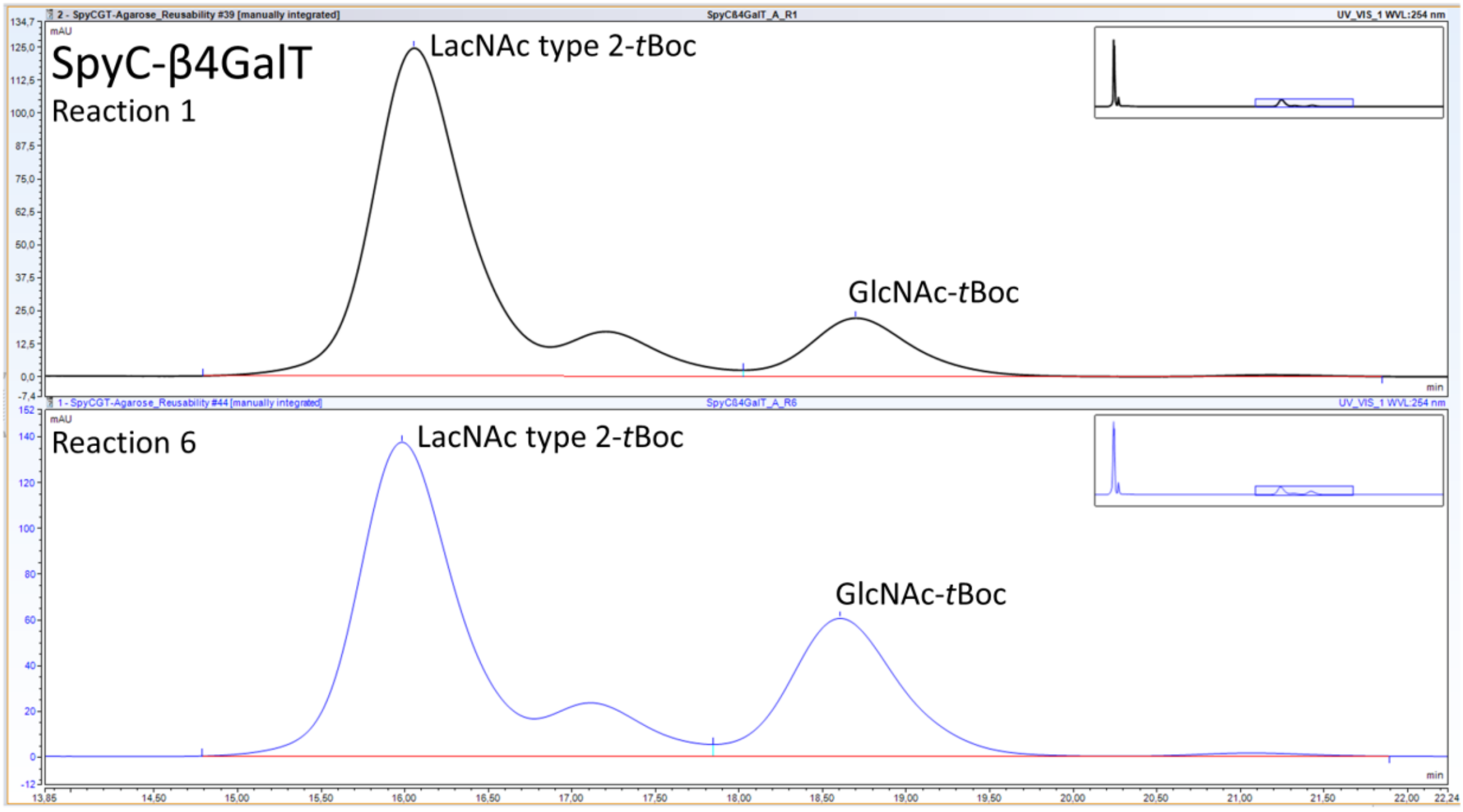

Fig. S21 HPLC chromatograms of SpyC-β4GalT reusability. A comparison between the first reaction on day 1 and the sixth reaction on day 3 is shown

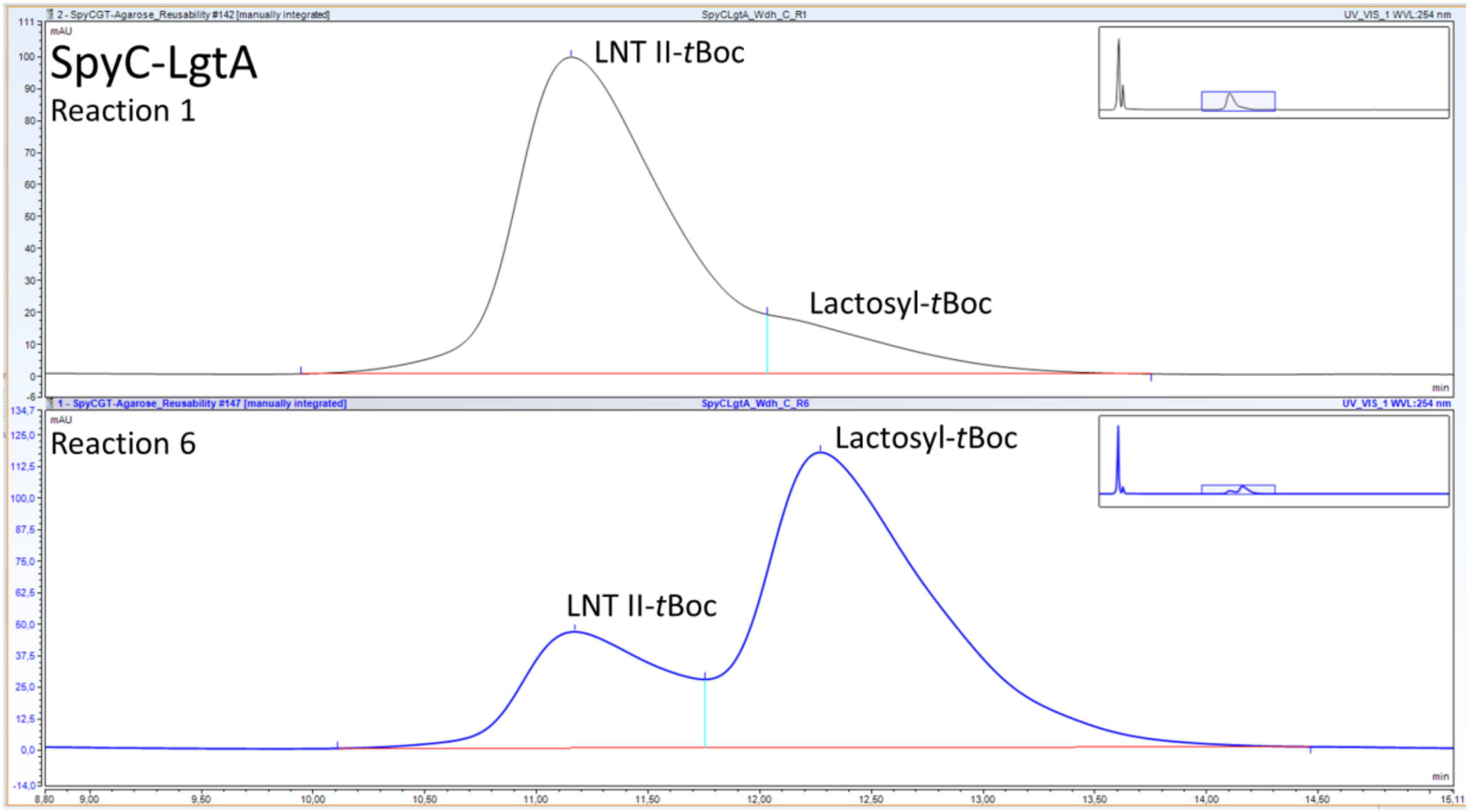

Fig. S22 HPLC chromatograms of SpyC-LgtA reusability. A comparison between the first reaction on day 1 and the sixth reaction on day 3 is shown

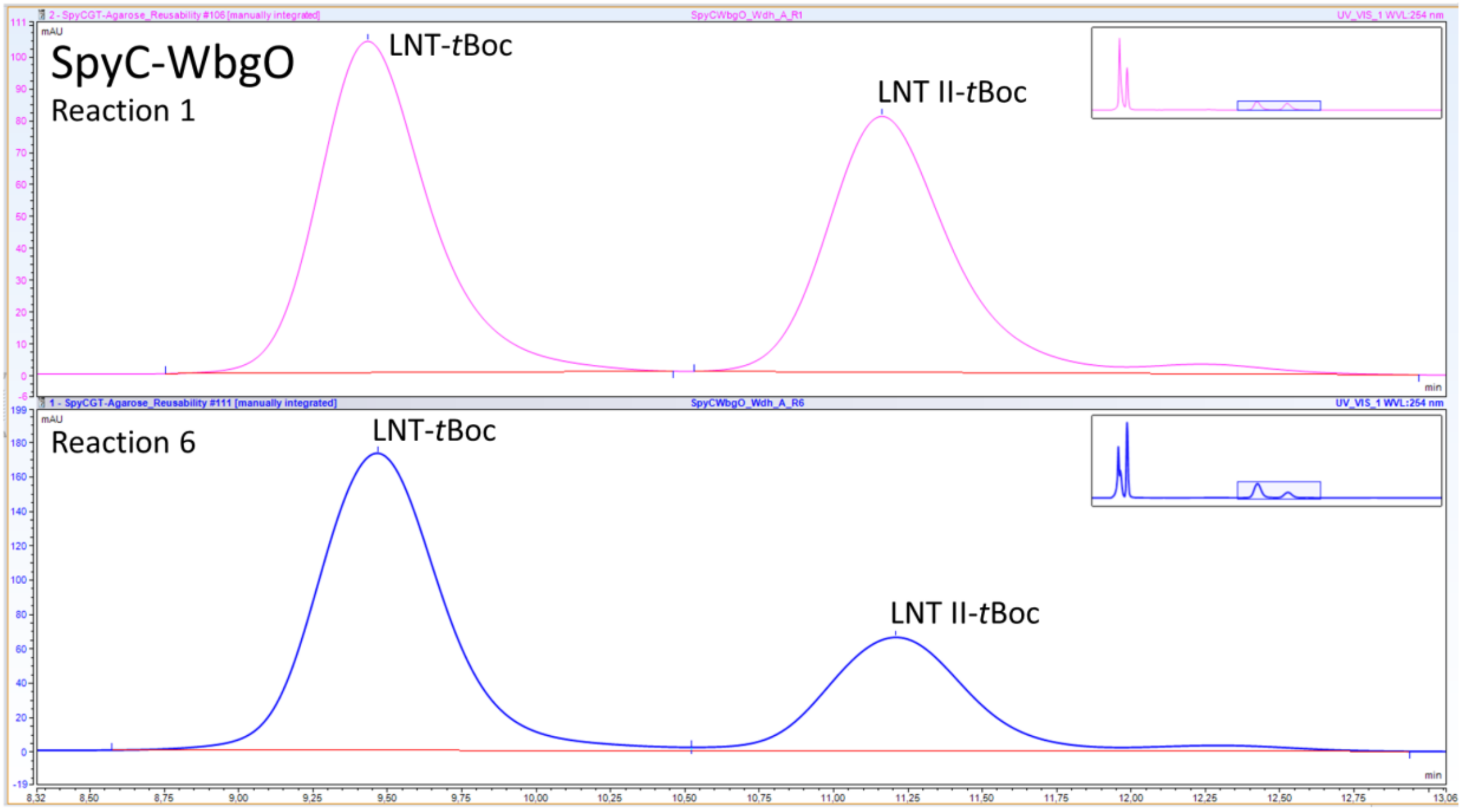

Fig. S23 HPLC chromatograms of SpyC-WbgO reusability. A comparison between the first reaction on day 1 and the sixth reaction on day 3 is shown

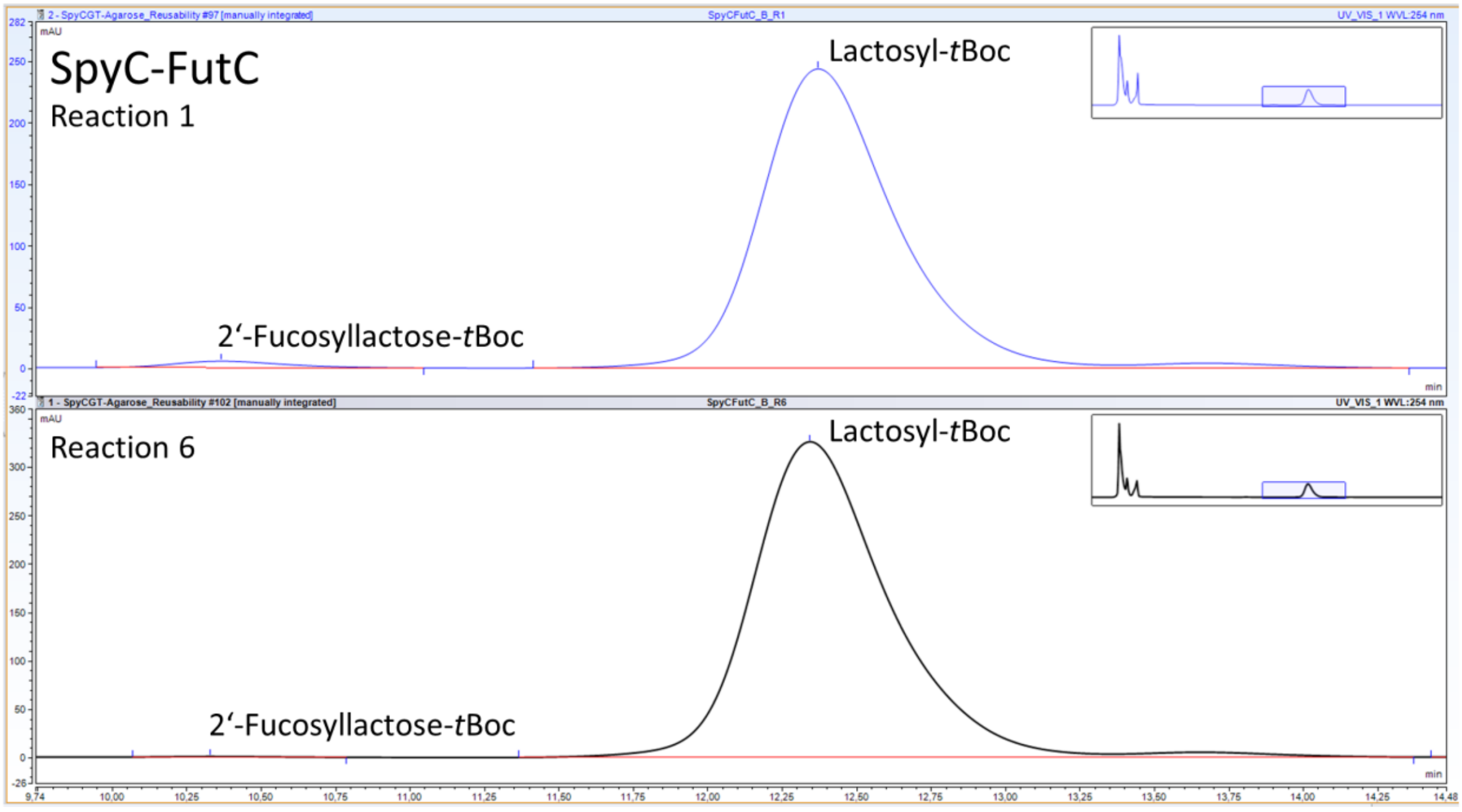

Fig. S24 HPLC chromatograms of SpyC-FutC reusability. A comparison between the first reaction on day 1 and the sixth reaction on day 3 is shown

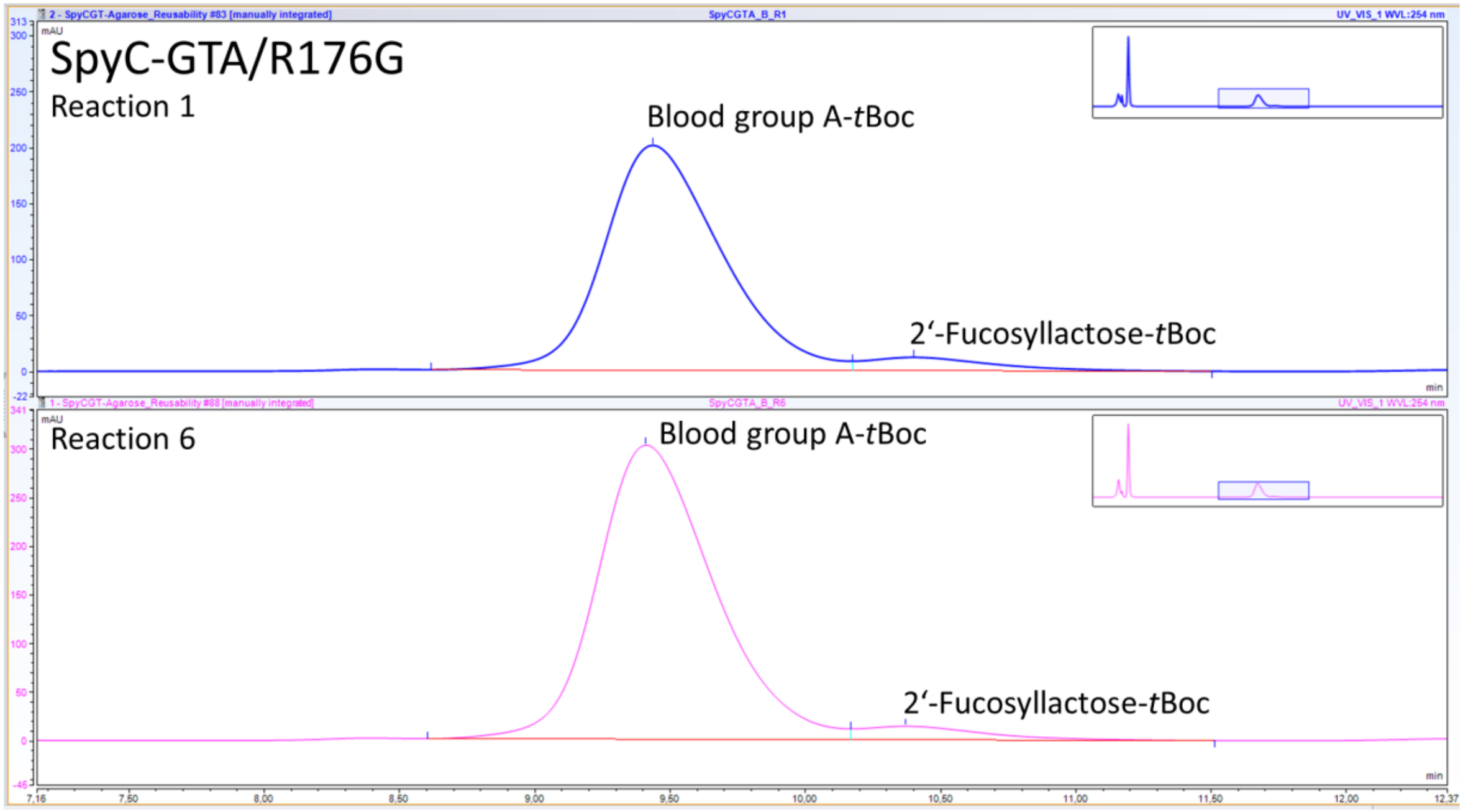

Fig. S25 HPLC chromatograms of SpyC-GTA/R176G reusability. A comparison between the first reaction on day 1 and the sixth reaction on day 3 is shown

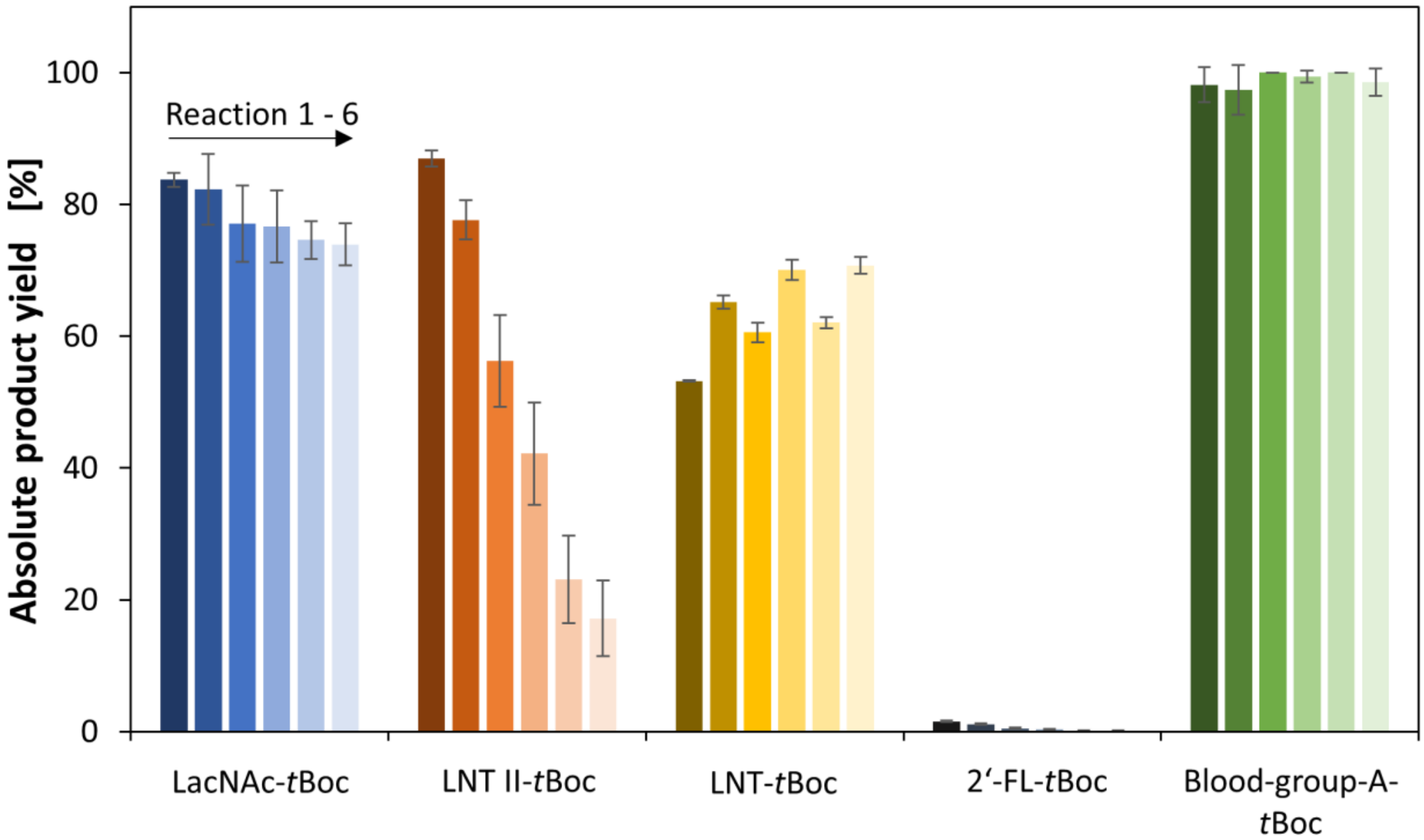

Fig. S26 Comparison of product yields for reusability assessment over six consecutive reactions with SpyT-agarose immobilized SpyC-GTs. The fading colors of bars represent subsequent reaction cycles over three days, with two reactions each day followed by storage overnight. LacNAc-*t*Boc (SpyC-β4GalT) [%]: 83.7, 82.3, 77.1, 76.6, 74.6, 73.9; LNT II-*t*Boc (SpyC-LgtA) [%]: 86.9, 77.6, 56.2, 42.2, 23.1, 17.2; LNT-*t*Boc (SpyC-WbgO) [%]: 53.2, 65.2, 60.6, 70.1, 62, 70.7; 2'-FL-*t*Boc (SpyC-FutC) [%]: 1.5, 1.1, 0.5, 0.4, 0.2, 0.2; Blood group A-*t*Boc (SpyC-GTA/R176G) [%]: 98.1, 97.4, 100, 99.4, 100, 98.5

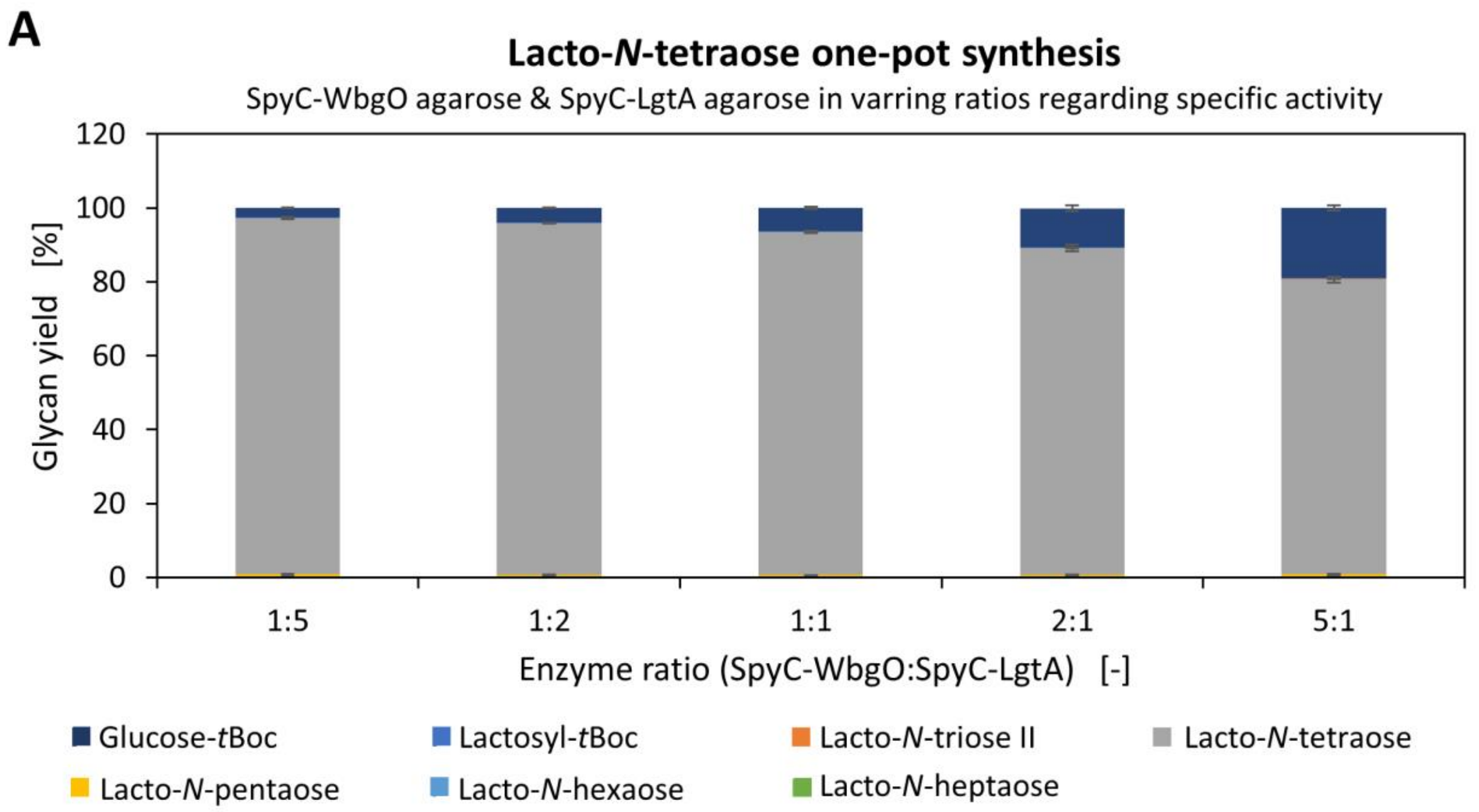

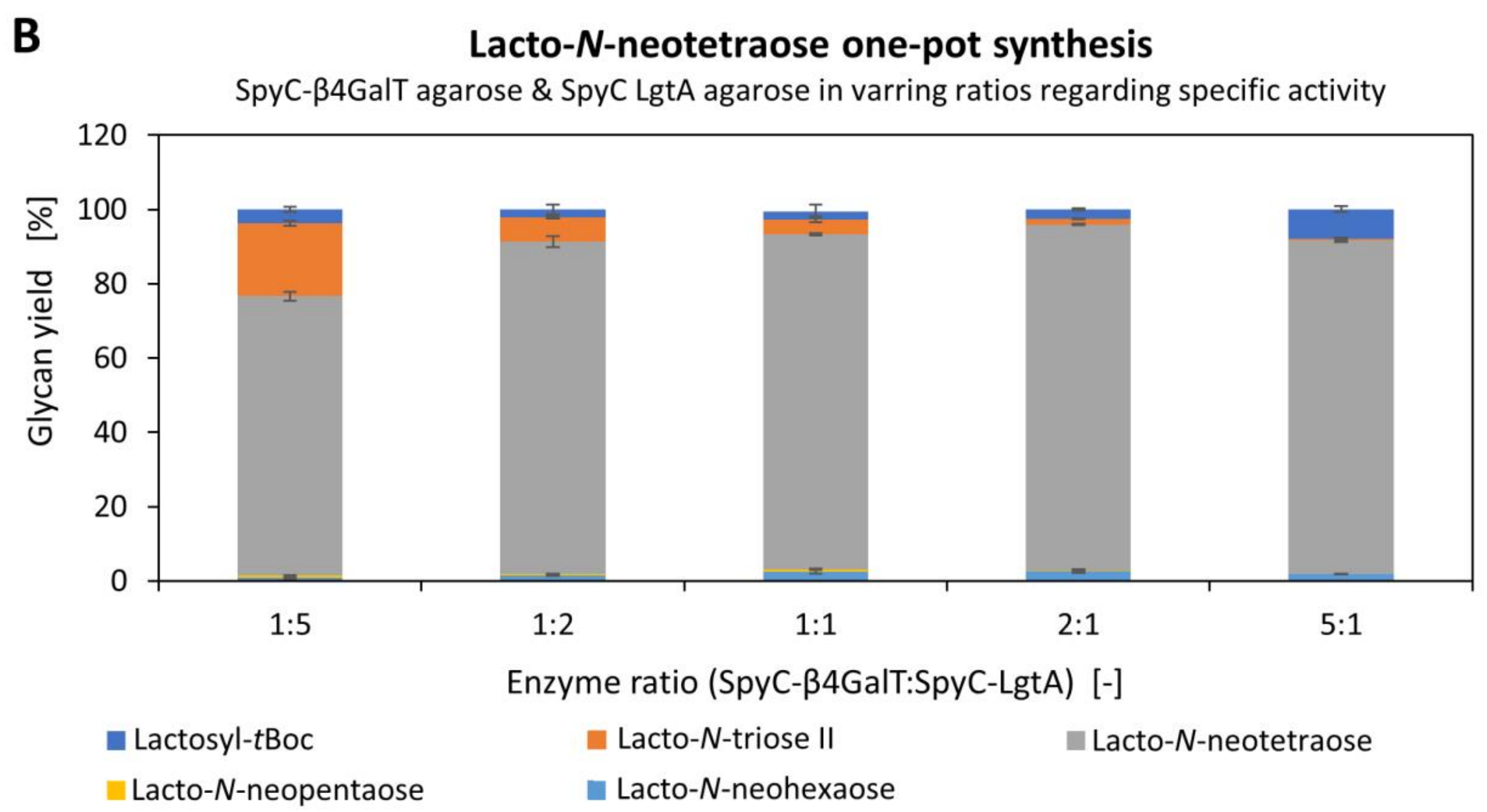

Fig. S27 Glycan yields for different enzyme ratios after 24 hours of reaction time. Glycan syntheses were carried out in one-pot approaches with varying enzyme ratios regarding their specific enzymatic activity (1:5, 1:2, 1:1, 2:1, and 5:1), starting with Lactosyl-*t*Boc (5 mM) as the initial acceptor substrate. Whereas tetraoses were the main product of all syntheses, residual educts and longer glycan structures were detected. **A)** Lacto-*N*-tetraose syntehses with SpyC-WbgO and SpyC-LgtA. Yields after 24 hours [%]. Ratio: 1:5: Glucose-*t*Boc (Glc): 2.6; Lactosyl-*t*Boc (Lac): < 1; Lacto-*N*-triose-*t*Boc (LNT II): 0; Lacto-*N*-tetraose-*t*Boc (LNT): 96.3; Lacto-*N*-pentaose-*t*Boc (LNP): < 1; Lacto-*N*-hexaose-*t*Boc (LNH): < 1; Lacto-*N*-heptaose-*t*Boc (LNHep): < 1. Ratio: 1:2: Glc: 4; Lac: < 1; LNT II: 0; LNT: 95.1; LNP: < 1; LNH: < 1; LNHep: < 1. Ratio 1:1: Glc: 6.4; Lac: < 1; LNT II: 0; LNT: 93; LNP: < 1; LNH: < 1; LNHep: < 1. Ratio 2:1: Glc: 10.6; Lac: < 1; LNT II: 0; LNT: 88.6; LNP: < 1; LNH: < 1; LNHep: < 1. Ratio 5:1: Glc: 19; Lac: < 1; LNT II: < 1; LNT: 80; LNP: < 1; LNH: < 1; LNHep: < 1. **B)** Lacto-*N*-neotetraose syntehses with SpyC-β4GalT and SpyC-LgtA. Yields after 24 hours [%]. Ratio 1:5: Lactosyl-*t*Boc (Lac): 3.7; Lacto-*N*-triose-*t*Boc (LNT II): 19.7; Lacto-*N*-neotetraose-*t*Boc (LNnT): 75.2; Lacto-*N*-neopentaose-*t*Boc (LNnP): < 1; Lacto-*N*-neohexaose-*t*Boc (LNnH): < 1. Ratio 1:2: Lac: 2.2; LNT II: 6.5; LNnT: 89.3; LNnP: < 1; LNnH: 1.5. Ratio 1:1: Lac: 2.2; LNT II: 4; LNnT: 90.3; LNnP: < 1; LNnH: 2.5. Ratio 2:1: Lac: 2.5; LNT II: 1.6; LNnT: 93.1; LNnP: < 1; LNnH: 2.6. Ratio 5:1: Lac: 8; LNT II: < 1; LNnT: 90; LNnP: 0; LNnH: 1.8

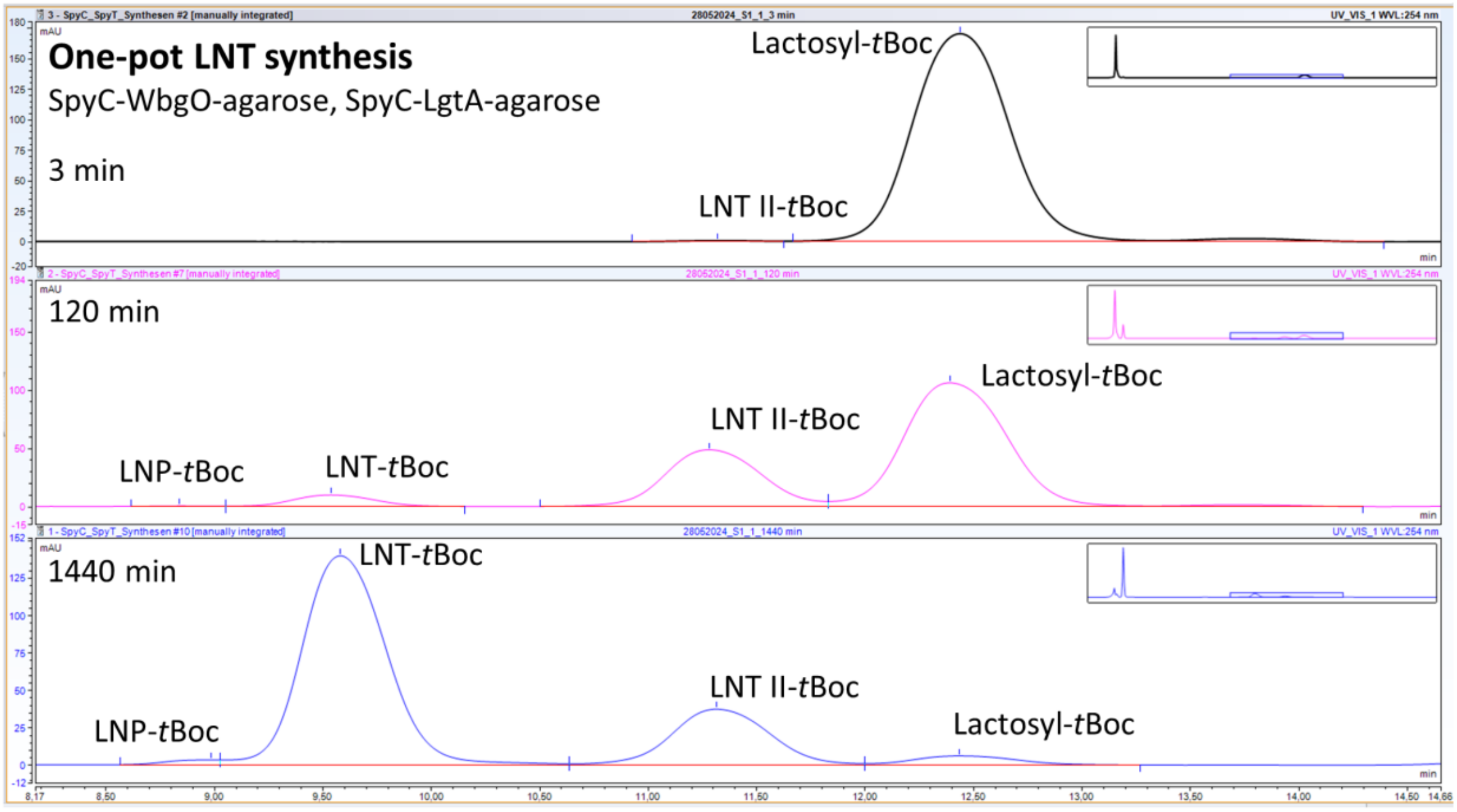

Fig. S28 HPLC chromatograms of the one-pot LNT synthesis with SpyT-agarose immobilized SpyC-WbgO and SpyC-LgtA. Peak areas represent product yields. 3 min: 99.7 % Lactosyl-*t*Boc, 0.3 % LNT II-*t*Boc ; 120 min: 66.7 % Lactosyl-*t*Boc, 28.5 % LNT II-*t*Boc , 4.8 % LNT-*t*Boc, 0.07 % LNP-*t*Boc; 1440 min: 3.5 % Lactosyl-*t*Boc, 21.8 % LNT II-*t*Boc , 73.9 % LNT-*t*Boc, 0.9 % LNP-*t*Boc

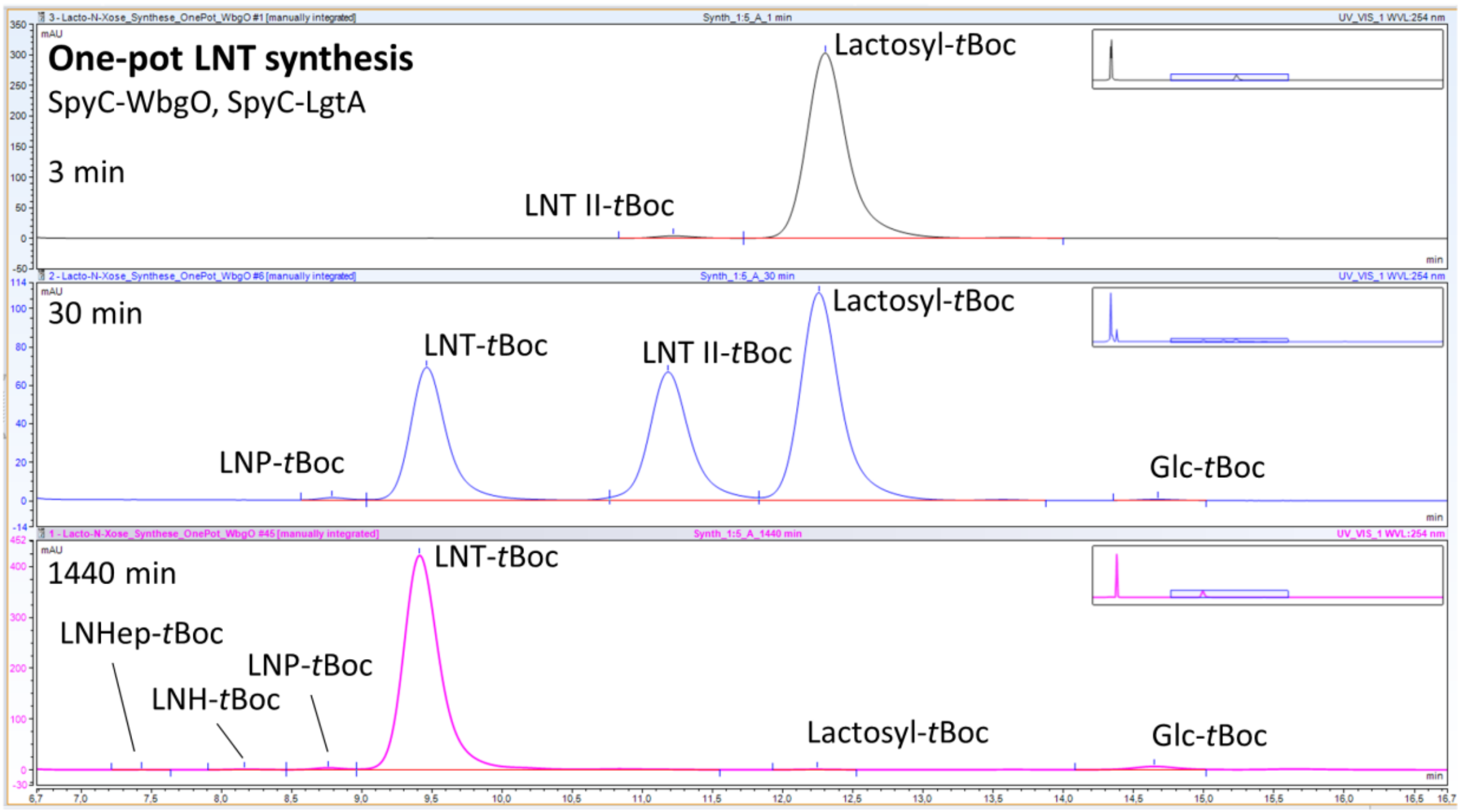

Fig. S29 HPLC chromatograms of the one-pot LNT synthesis with free SpyC-WbgO and SpyC-LgtA. Peak areas represent product yields. 3 min: 99.0 % Lactosyl-*t*Boc, 1.0 % LNT II-*t*Boc ; 30 min: 0.2 % Glc-*t*Boc; 48.8 % Lactosyl-*t*Boc, 26.3 % LNT II-*t*Boc , 24.3 % LNT-*t*Boc, 0.3 % LNP-*t*Boc; 1440 min: 2.6 % Glc-*t*Boc; 0.2 % Lactosyl-*t*Boc, 96.3 % LNT-*t*Boc, 0.8 % LNP-*t*Boc, 0.2 % LNH-*t*Boc, 0.02 % LNHep-*t*Boc

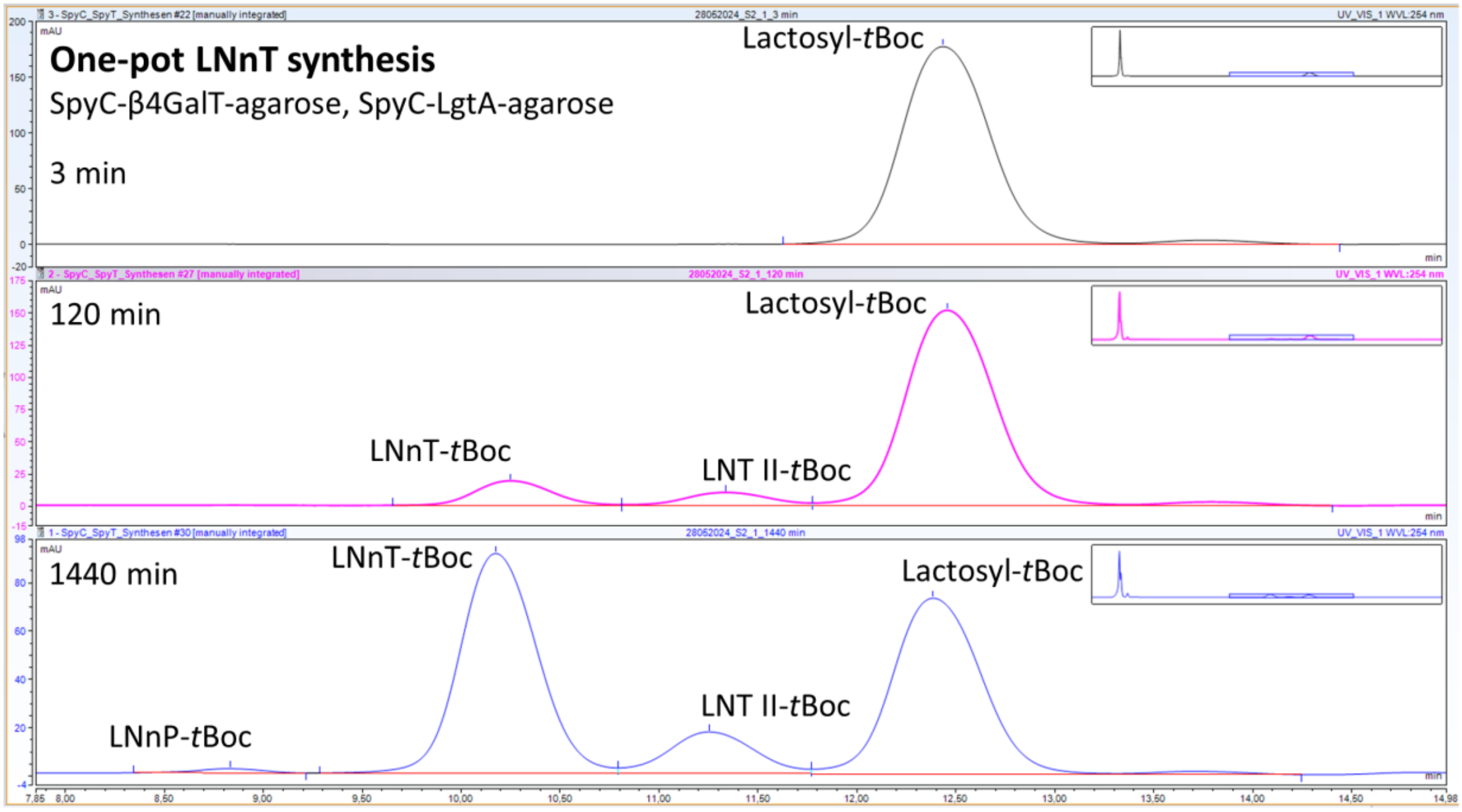

Fig. S30 HPLC chromatograms of the one-pot LNnT synthesis with SpyT-agarose immobilized SpyC-β4GalT and SpyC-LgtA. Peak areas represent product yields. 3 min: 100 % Lactosyl-*t*Boc; 120 min: 84.9 % Lactosyl-*t*Boc, 5.4 % LNT II-*t*Boc , 9.7 % LNnT-*t*Boc; 1440 min: 43.1 % Lactosyl-*t*Boc, 9.5 % LNT II-*t*Boc , 46.6 % LNnT-*t*Boc, 0.8 % LNnP-*t*Boc

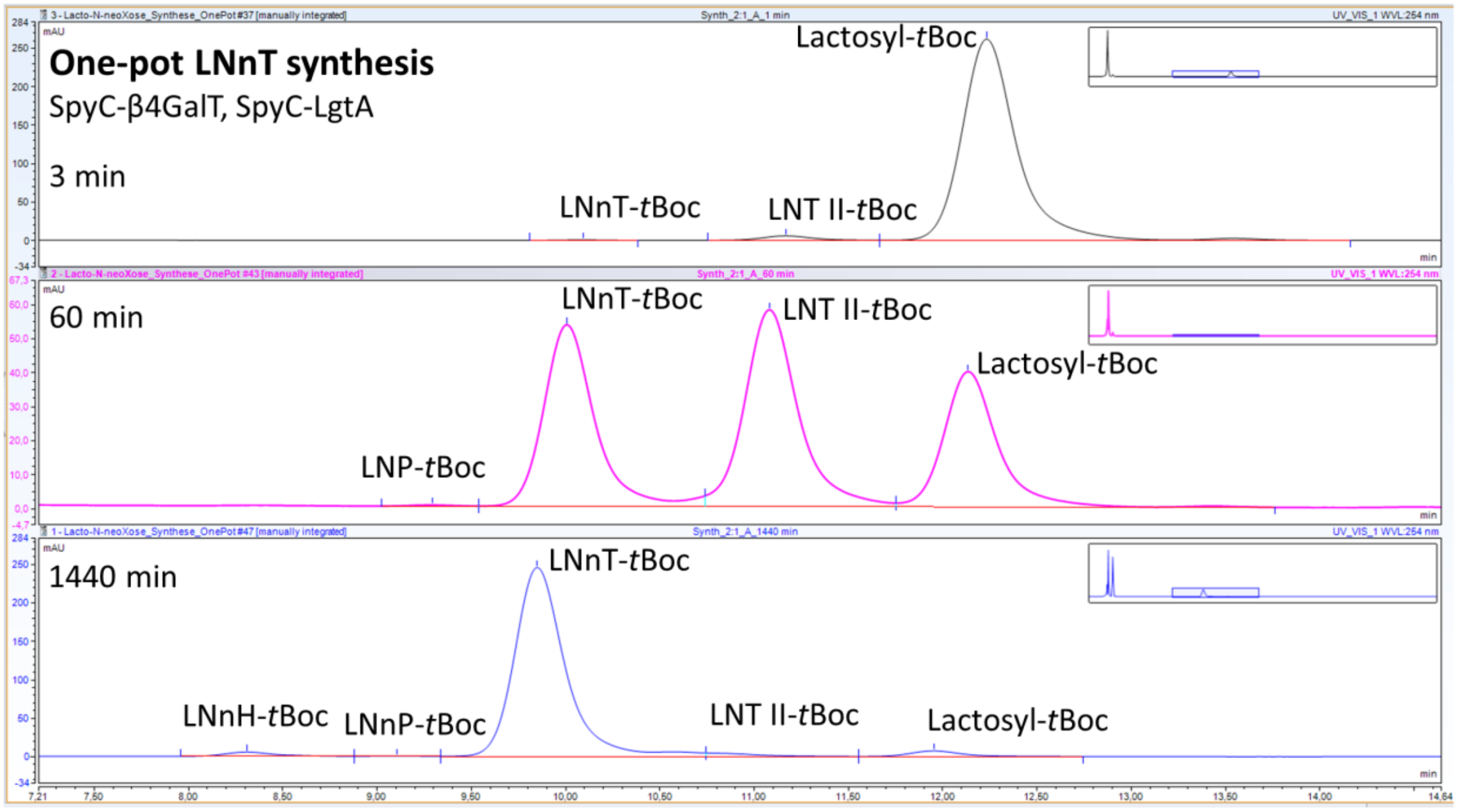

Fig. S31 HPLC chromatograms of the one-pot LNnT synthesis with free SpyC-β4GalT and SpyC-LgtA. Peak areas represent product yields. 3 min: 97.8 % Lactosyl-*t*Boc, 2.1 % LNT II-*t*Boc, 0.2 % LNnT-*t*Boc; 60 min: 24.6 % Lactosyl-*t*Boc, 40.9 % LNT II-*t*Boc , 34.3 % LNnT-*t*Boc, 0.2 % LNnP-*t*Boc; 1440 min: 2.5 % Lactosyl-*t*Boc, 1.6 % LNT II-*t*Boc , 93.1 % LNnT-*t*Boc, 0.2 % LNnP-*t*Boc, 2.6 % LNnH-*t*Boc

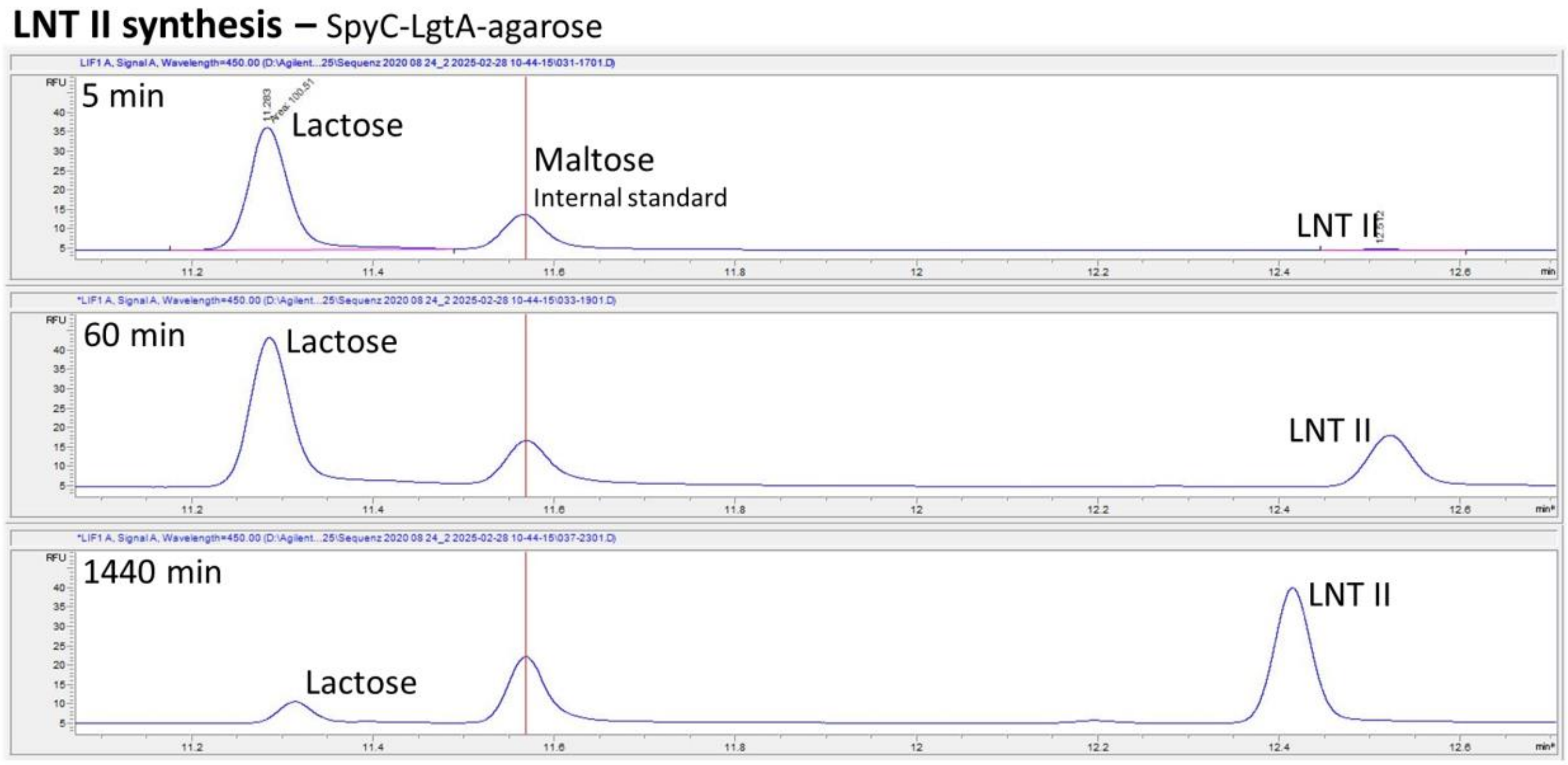

Fig. S32 CE-LIF chromatograms of the LNT II synthesis with SpyT-agarose immobilized SpyC-LgtA. Peak areas represent product yields. 1 mM Maltose was used as internal standard. 5 min: 99.5 % Lactose, 0.5 % LNT II; 60 min: 71.9 % Lactose, 28.1 % LNT II; 1440 min: 13.9 % Lactose, 86.1 % LNT II

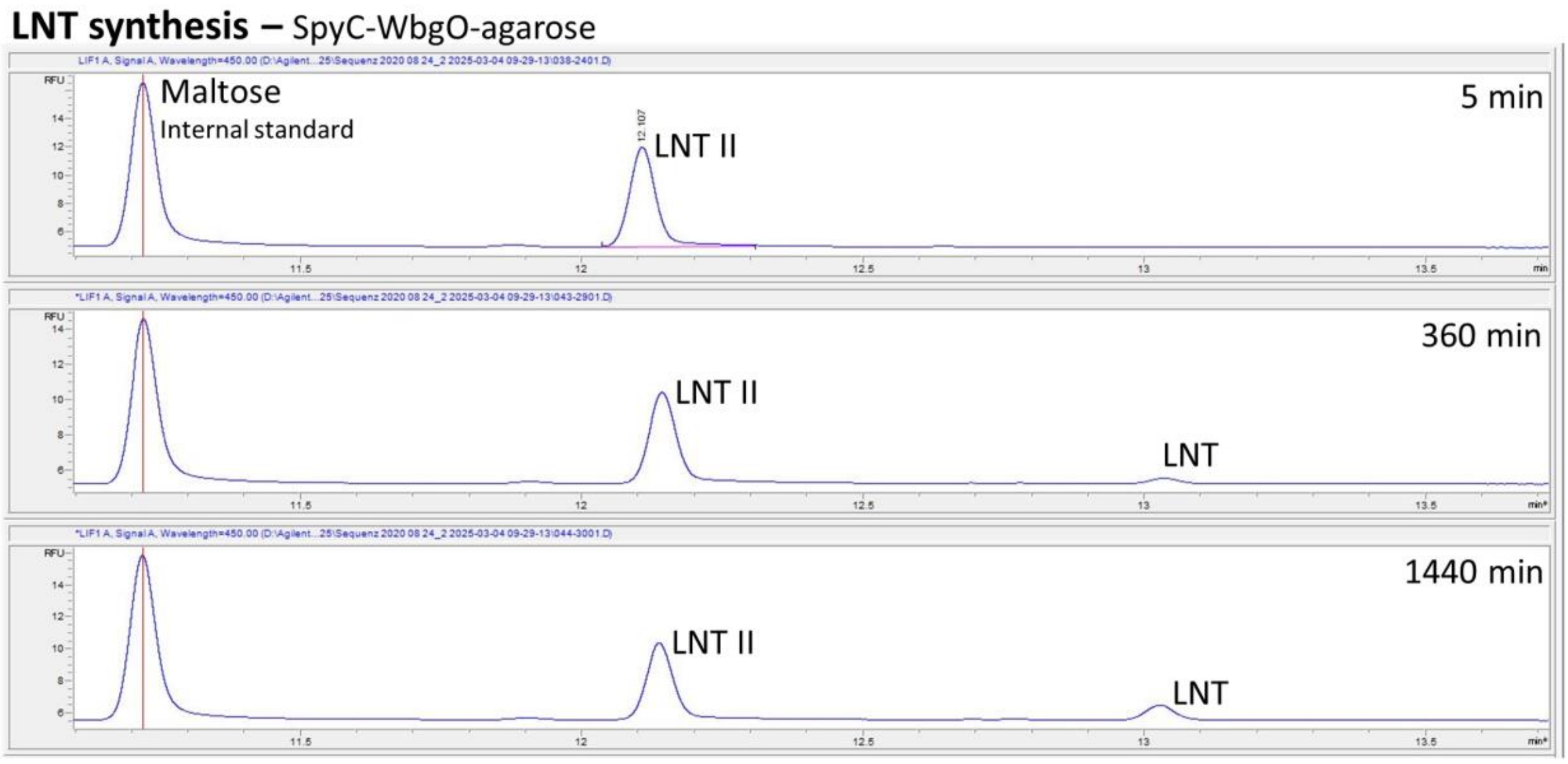

Fig. S33 CE-LIF chromatograms of the LNT synthesis with SpyT-agarose immobilized SpyC-WbgO. Peak areas represent product yields. 1 mM Maltose was used as internal standard. 5 min: 100 % LNT II, 0 % LNT; 360 min: 93.6 % LNT II, 6.4 % LNTI; 1440 min: 80.1 % LNT II, 19.9 % LNT

LNnT synthesis – SpyC-β4GalT-agarose

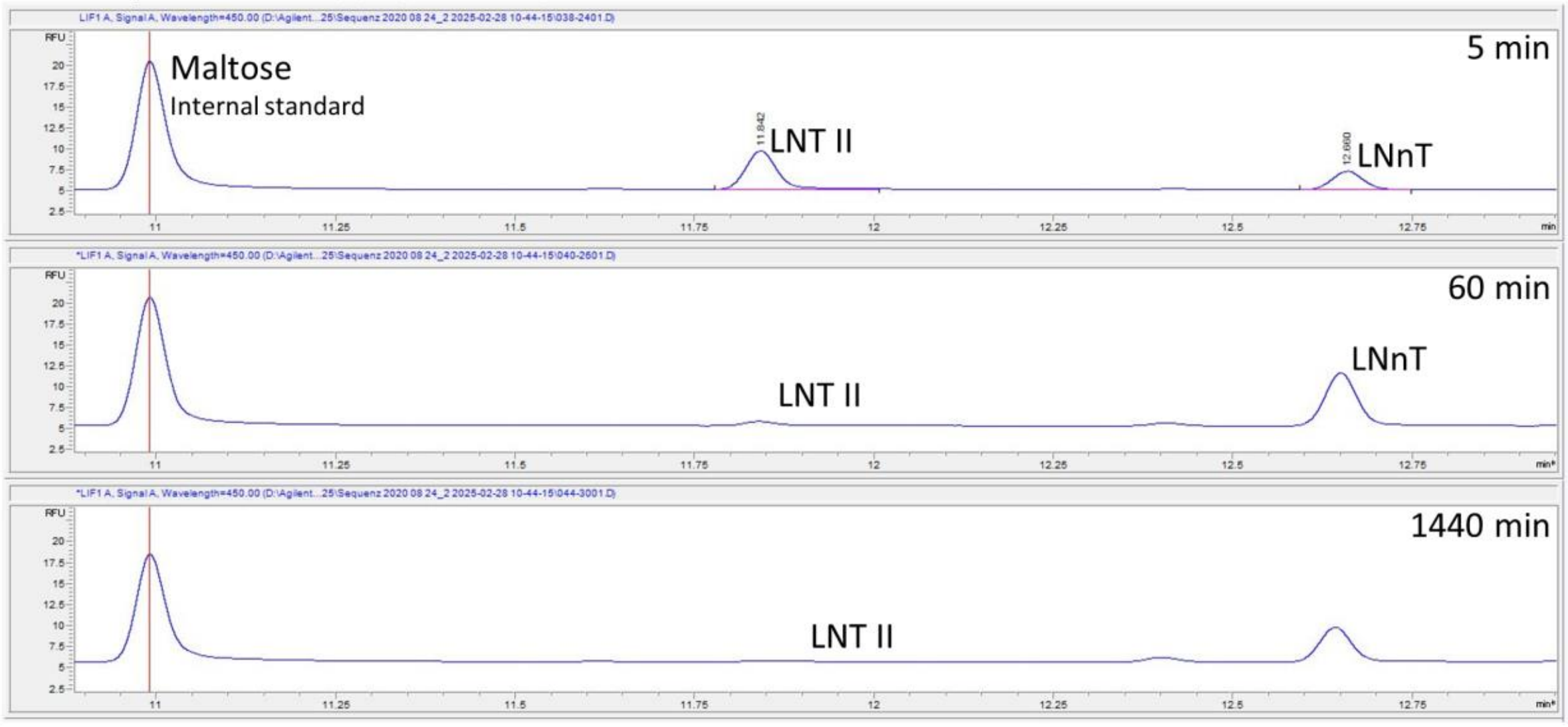

Fig. S34 CE-LIF chromatograms of the LNnT synthesis with SpyT-agarose immobilized SpyC-β4GalT. Peak areas represent product yields. 1 mM Maltose was used as internal standard. 5 min: 72.1 % LNT II, 27.9 % LNnT; 60 min: 8.8 % LNT II, 91.2 % LNnT; 1440 min: 6.8 % LNT II, 93.2 % LNnT

LNFP I synthesis – His_6-LPP-FutC

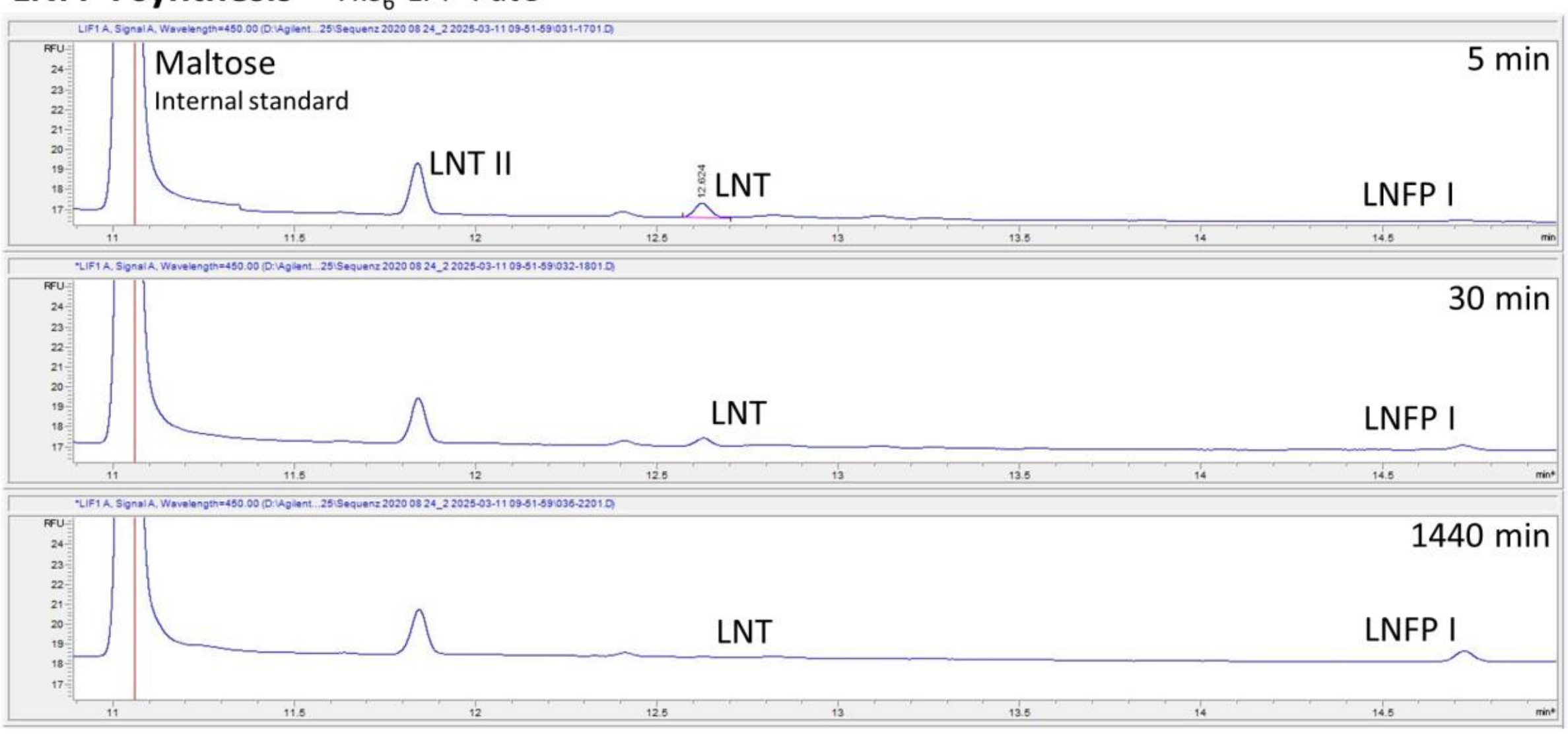

Fig. S35 CE-LIF chromatograms of the LNFP I synthesis with His_6-LPP-FutC. Peak areas represent product yields. 1 mM Maltose was used as internal standard. Residual LNT II can be seen in all chromatograms. 5 min: 93.0 % LNT, 7.0 % LNFP I; 30 min: 60.0 % LNT, 40.0 % LNFP I; 1440 min: 4.4 % LNT, 95.6 % LNFP I

LNnFP I synthesis – His$_6$-LPP-FutC

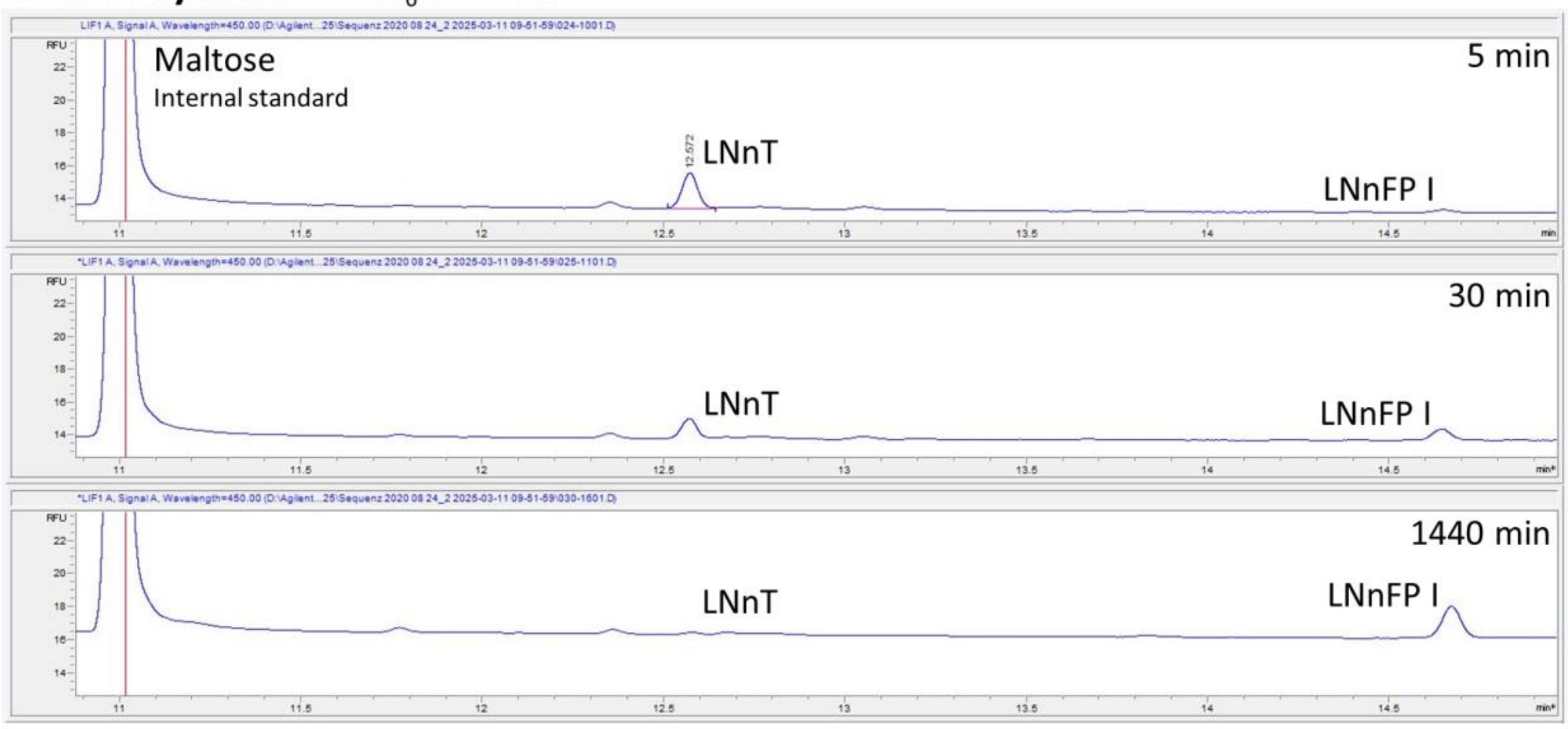

Fig. S36 CE-LIF chromatograms of the LNnFP I synthesis with His$_6$-LPP-FutC. Peak areas represent product yields. 1 mM Maltose was used as internal standard. 5 min: 85.5 % LNnT, 14.5 % LNnFP I; 30 min: 58.2 % LNnT, 41.8 % LNnFP I; 1440 min: 12.2 % LNnT, 87.8 % LNnFP I

BGA hexaose I synthesis – SpyC-GTA/R176G-agarose

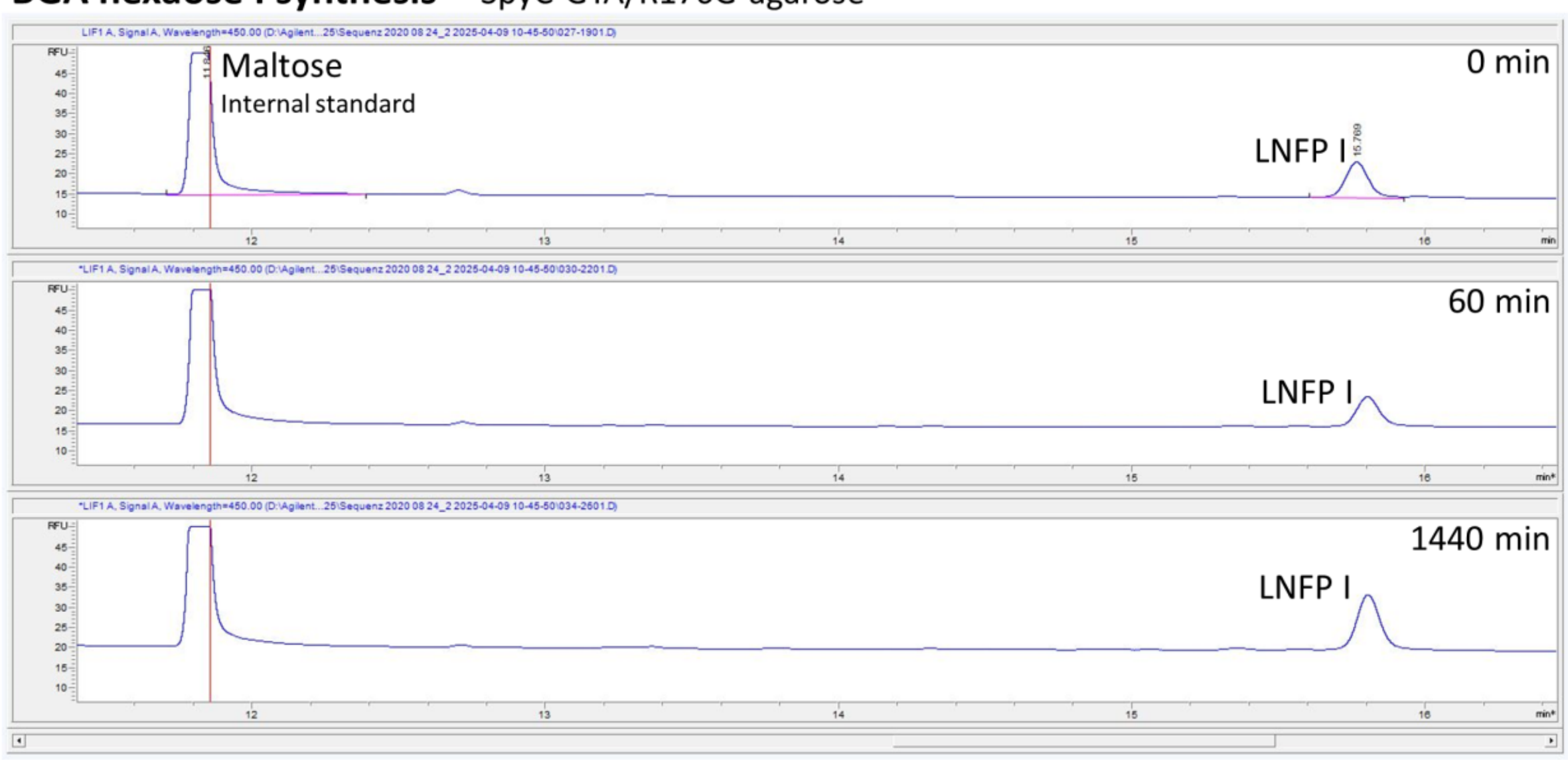

Fig. S37 CE-LIF chromatograms of the BGA hexaose I synthesis with SpyT-agarose immobilized SpyC-GTA/R176G. Peak areas represent product yields. 1 mM Maltose was used as internal standard. Even after 24 hours, BGA hexaose I was not detectable

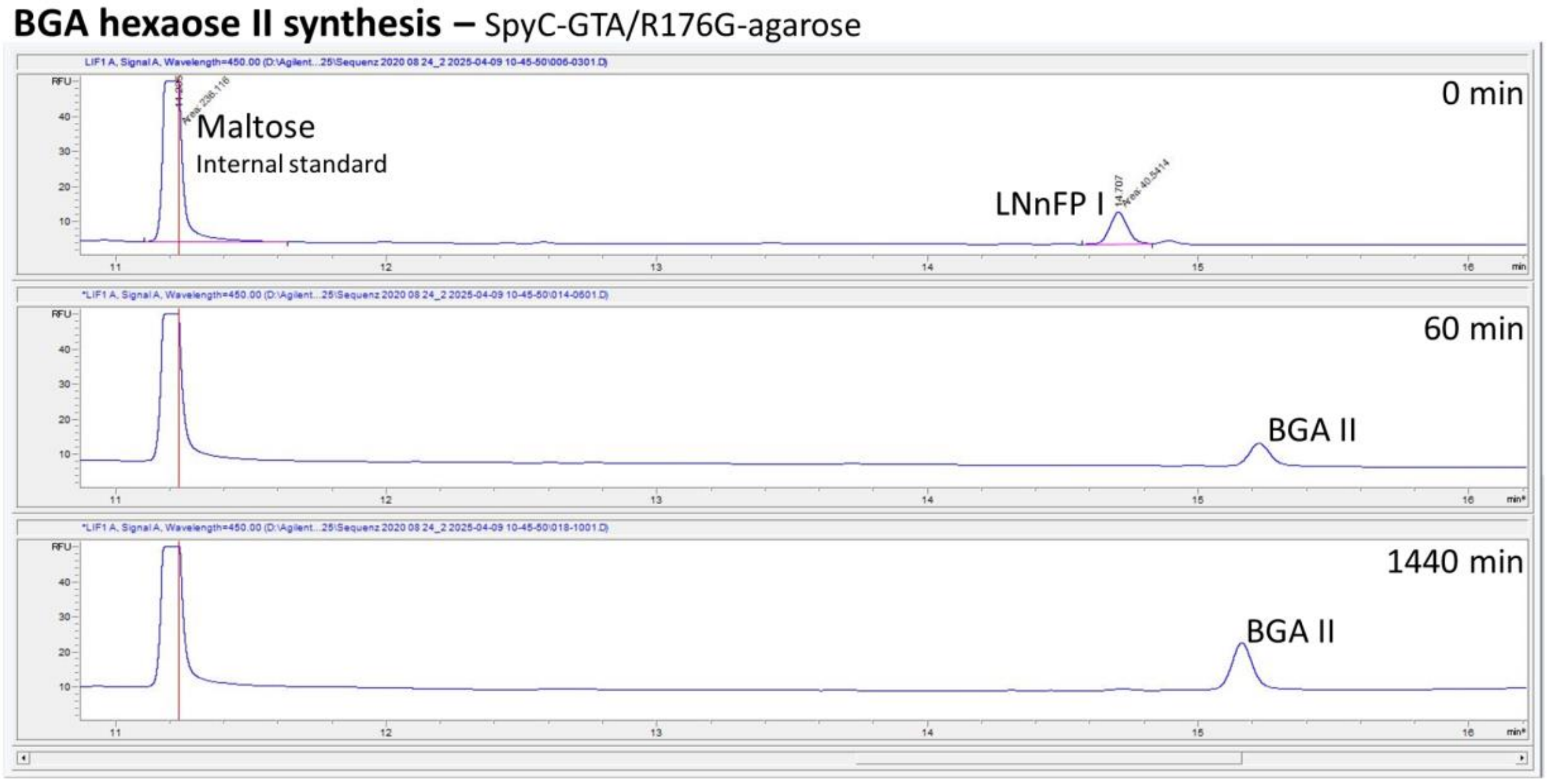

Fig. S38 CE-LIF chromatograms of the BGA hexaose II synthesis with SpyT-agarose immobilized SpyC-GTA/R176G. Peak areas represent product yields. 1 mM Maltose was used as internal standard. 0 min: 100 % LNnFP I, 0 % BGA II; 60 min: 0 % LNnFP I, 100 % BGA II

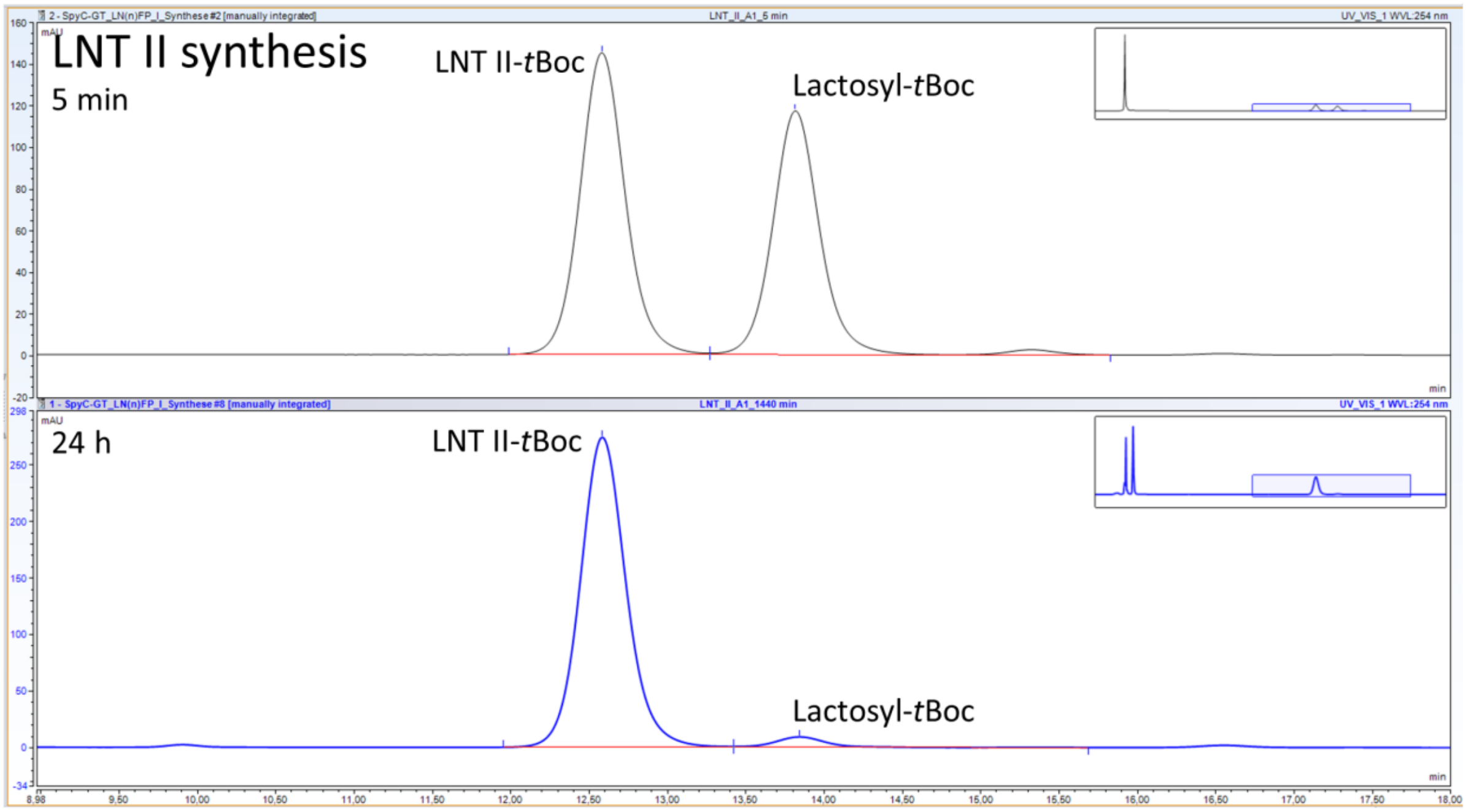

Fig. S39 HPLC chromatograms of the LNT II synthesis with SpyC-LgtA. Peak areas represent product yields. 5 min: 45.9 % Lactosyl-*t*Boc, 54.1 % LNT II-*t*Boc; 24 h: 4.1 % Lactosyl-*t*Boc, 95.9 % LNT II-*t*Boc

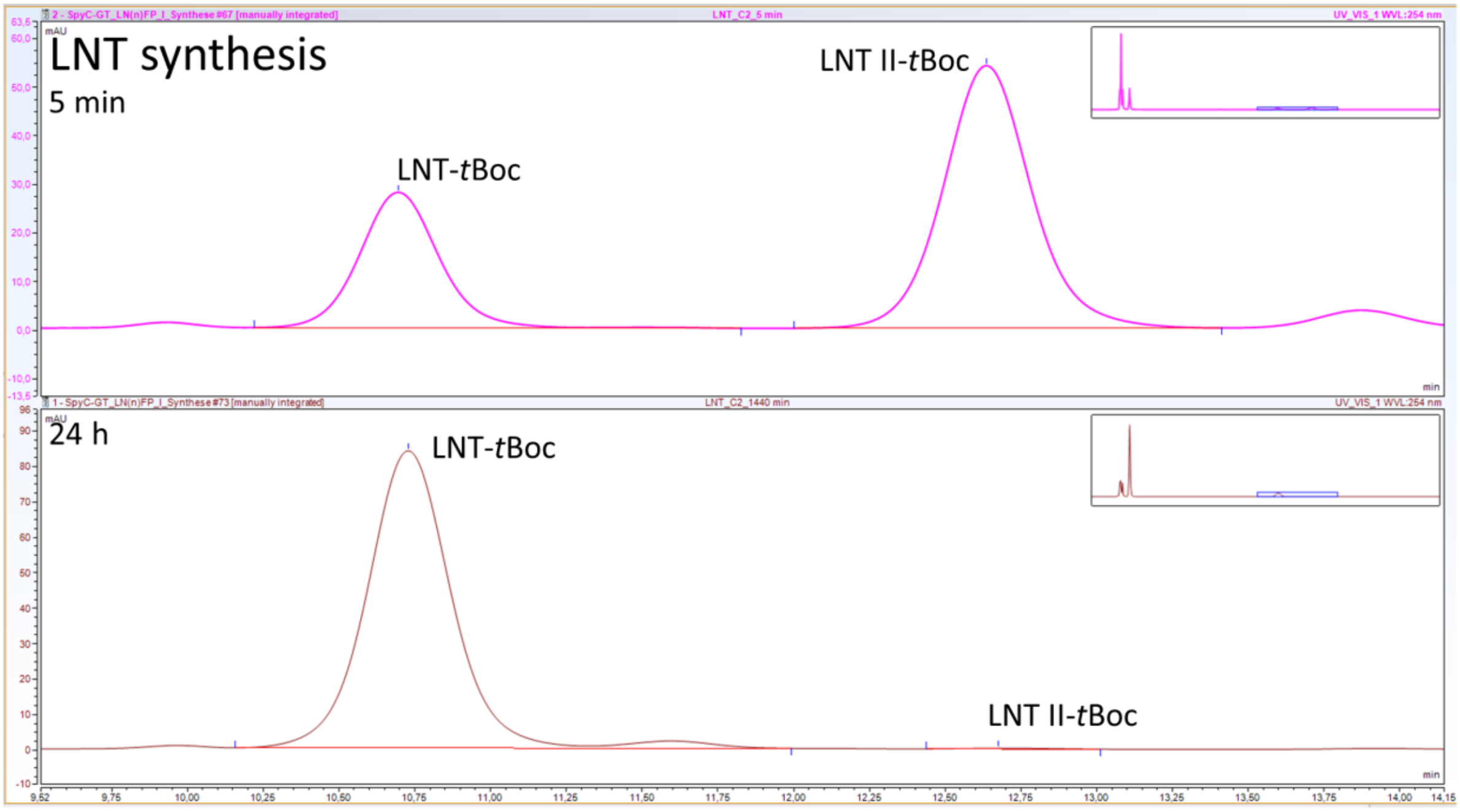

Fig. S40 HPLC chromatograms of the LNT synthesis with SpyC-WbgO. Peak areas represent product yields. 5 min: 68.3 % LNT II-*t*Boc, 31.7 % LNT-*t*Boc; 24 h: 0.3 % LNT II-*t*Boc, 99.7 % LNT-*t*Boc

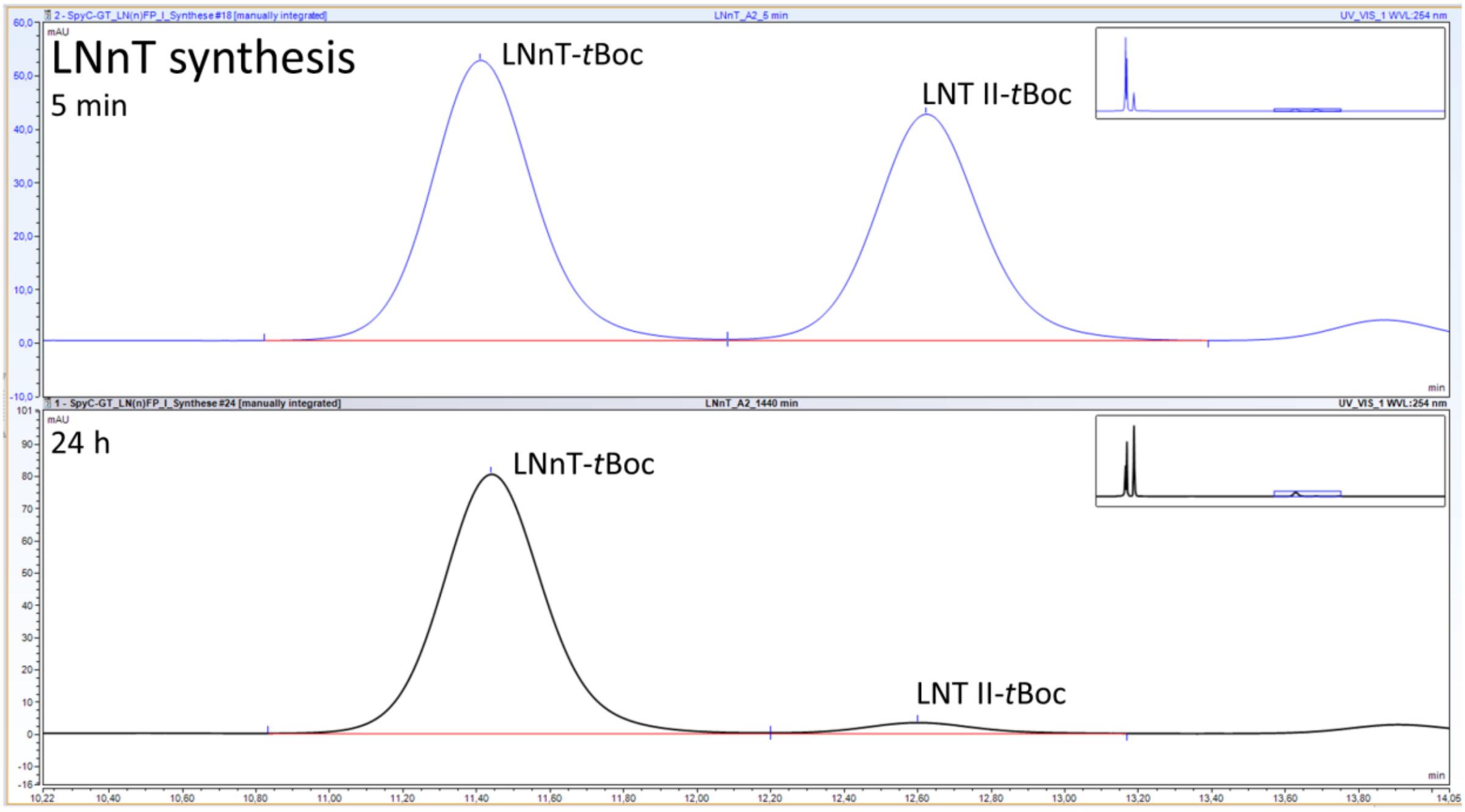

Fig. S41 HPLC chromatograms of the LNnT synthesis with SpyC-β4GalT. Peak areas represent product yields. 5 min: 45.3 % LNT II-*t*Boc, 54.7 % LNnT-*t*Boc; 24 h: 4.3 % LNT II-*t*Boc, 95.7 % LNnT-*t*Boc

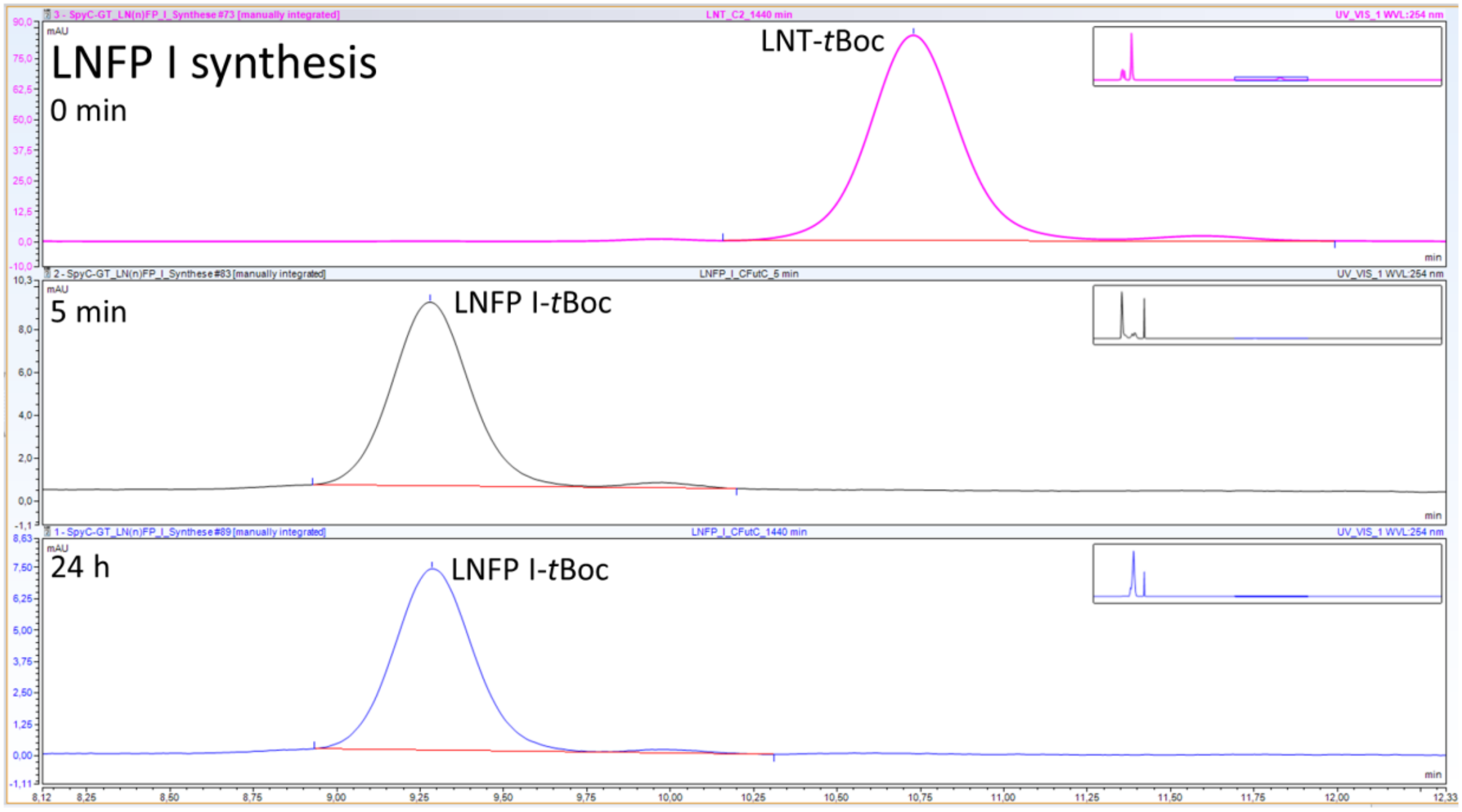

Fig. S42 HPLC chromatograms of the LNFP I synthesis with His_6-LPP-FutC. Peak areas represent product yields. 5 min: 0 % LNT-*t*Boc, 100 % LNFP I-*t*Boc

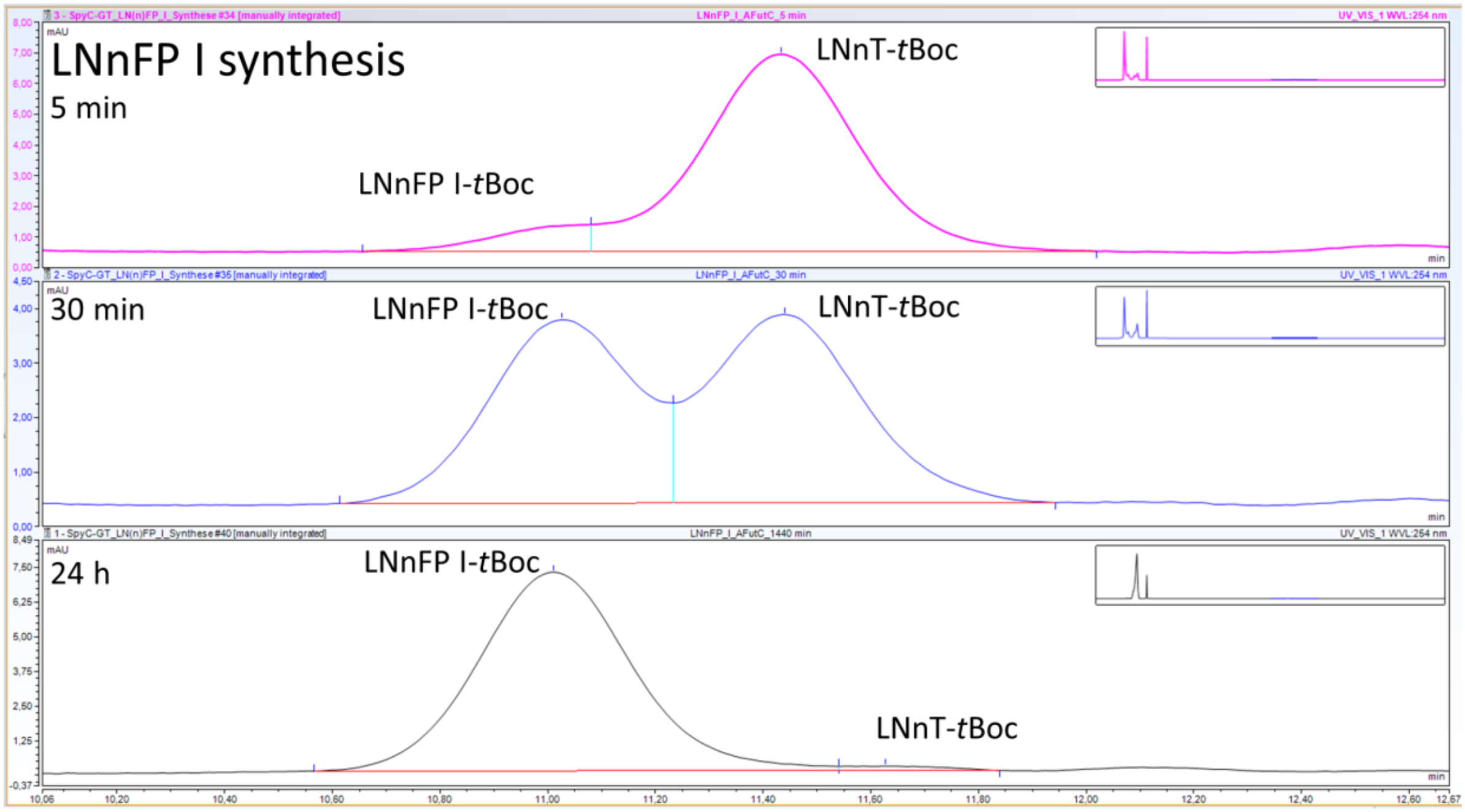

Fig. S43 HPLC chromatograms of the LNnFP I synthesis with His_6-LPP-FutC. Peak areas represent product yields. 5 min: 93.4 % LNnT-*t*Boc, 6.6 % LNnFP I-*t*Boc; 30 min: 52.1 % LNnT-*t*Boc, 47.9 % LNnFP I-*t*Boc; 24 h: 1.5 % LNnT-*t*Boc, 98.5 % LNnFP I-*t*Boc

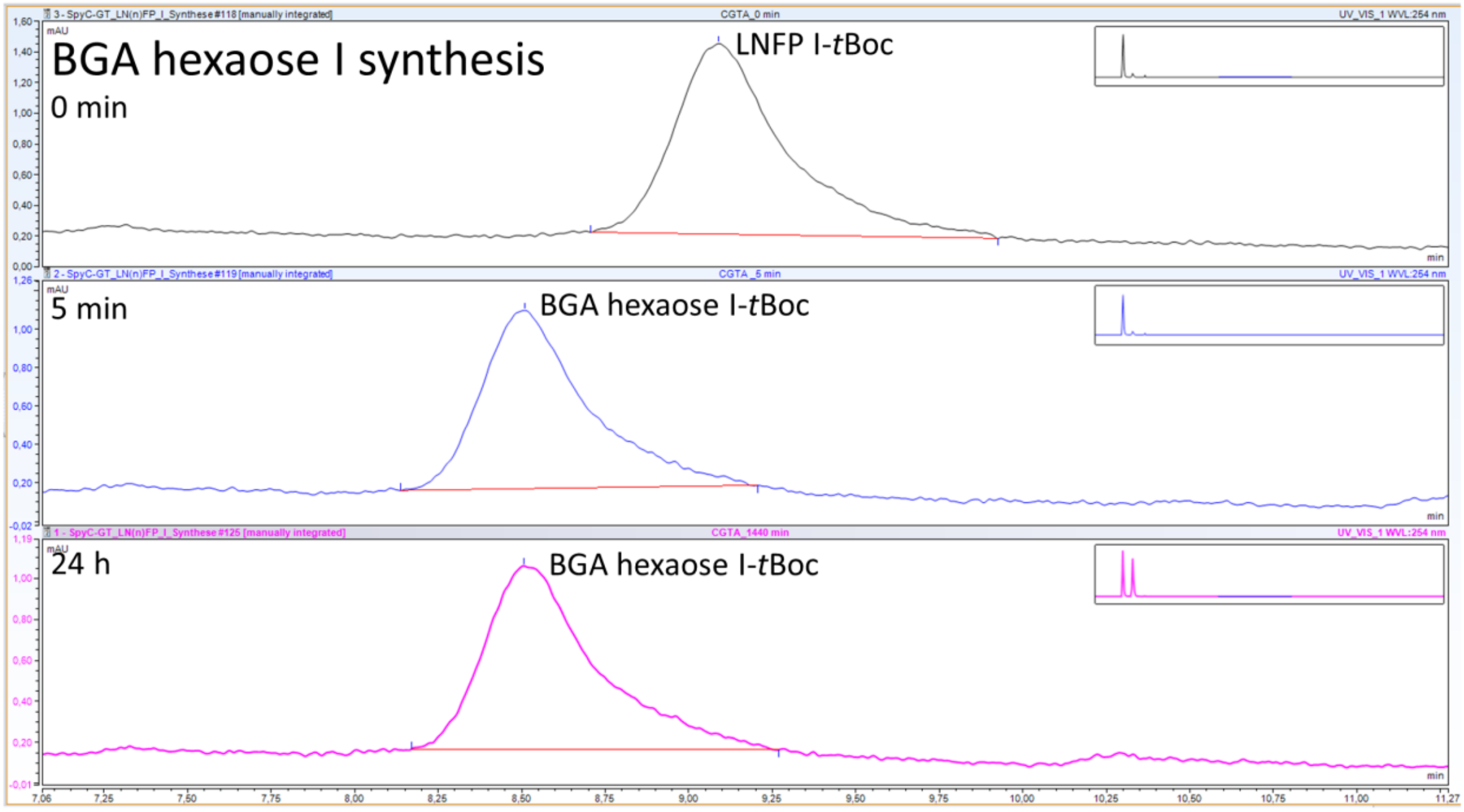

Fig. S44 HPLC chromatograms of the BGA hexaose I synthesis with SpyC-GTA/R176G. Peak areas represent product yields. 5 min: 0 % LNFP I-*t*Boc, 100 % BGA hexaose I-*t*Boc

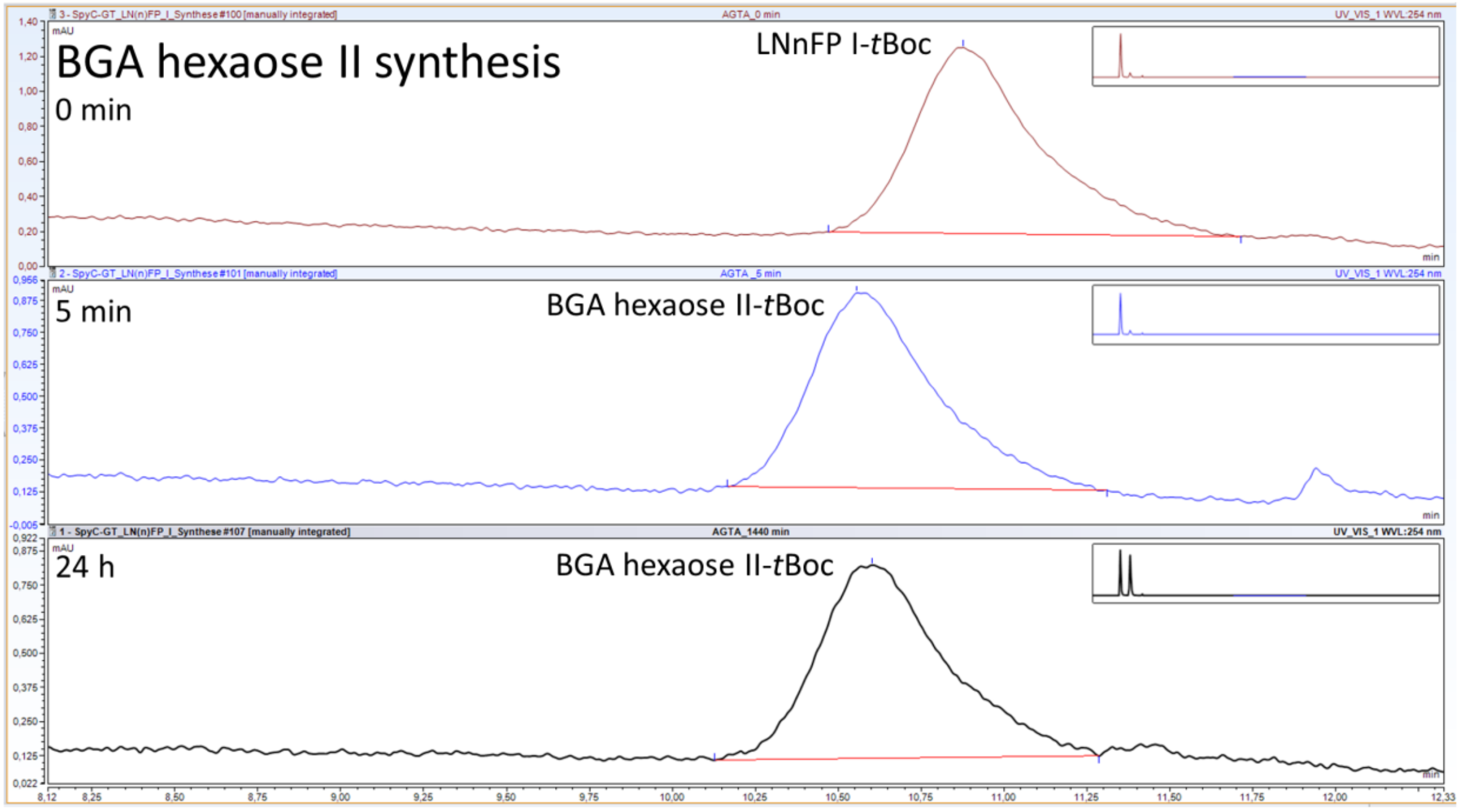

Fig. S45 HPLC chromatograms of the BGA hexaose II synthesis with SpyC-GTA/R176G. Peak areas represent product yields. 5 min: 0 % LNnFP I-*t*Boc, 100 % BGA hexaose II-*t*Boc

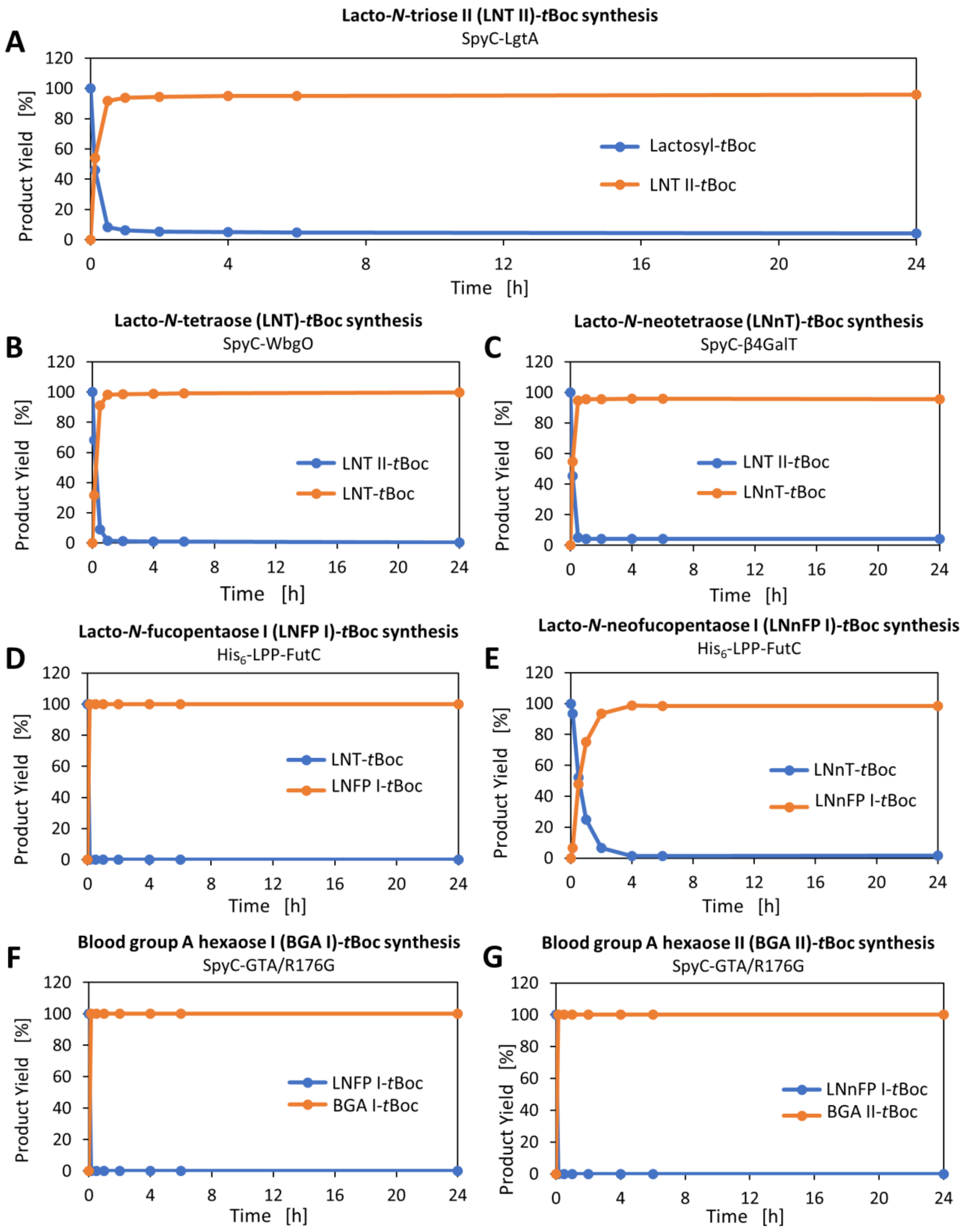

Fig. S46 Sequential glycan syntheses. Lactosyl-*t*Boc (5 mM) as initial acceptor. LNT II-*t*Boc, LNT-*t*Boc, LNnT-*t*Boc, Blood group A hexaose I, and Blood group A hexaose II syntheses were done with SpyC-GTs. Fucosylation was achieved with His_6-LPP-FutC, obtaining the products Lacto-*N*-fucopentaose I (LNFP I)-*t*Boc and Lacto-*N*-neofucopentaose I (LNnFP I)-*t*Boc. **A)** LNT II-*t*Boc synthesis. Yield after 30 min: 91 %. **B)** LNT-*t*Boc synthesis. Yield after 30 min: 91 %. **C)** LNnT-*t*Boc synthesis. Yield after 30 min: 94 %. **D)** LNFP I synthesis. Yield after 5 min: 100 %. **E)** LNnFP I synthesis. Yield after 2 hours: 93 %. **F)** BGA I synthesis. Yield after 5 min: 100 %. **G)** BGA II synthesis. Yield after 5 min: 100 %

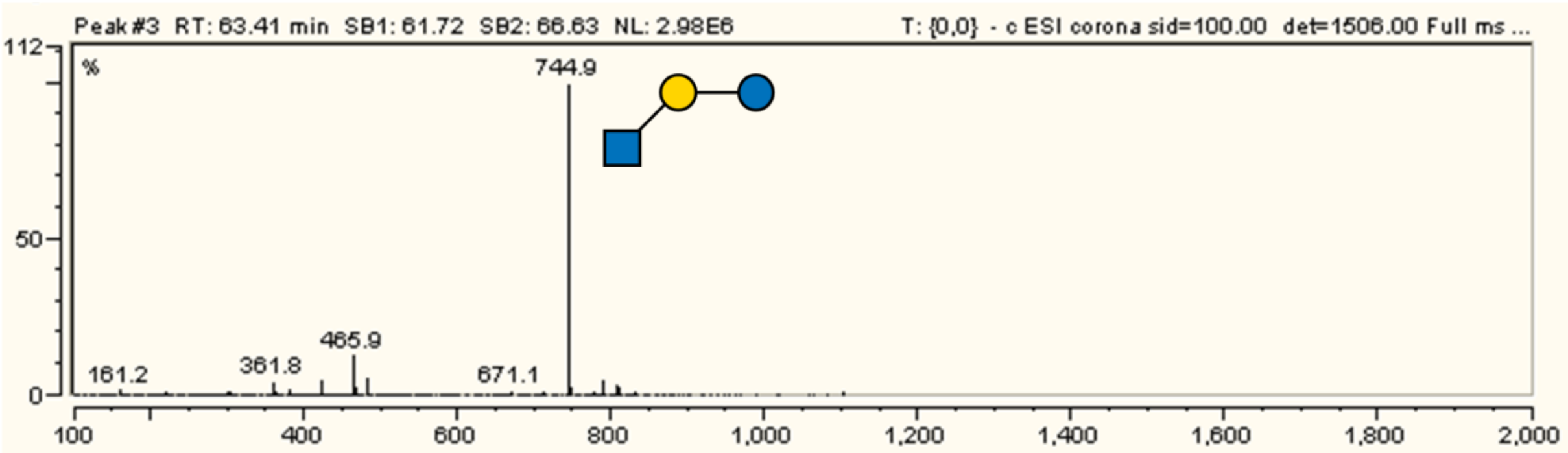

Fig. S47 Mass spectrum (ESI-) of LNT II-*t*Boc. [M-H]⁻, m/z 744.9

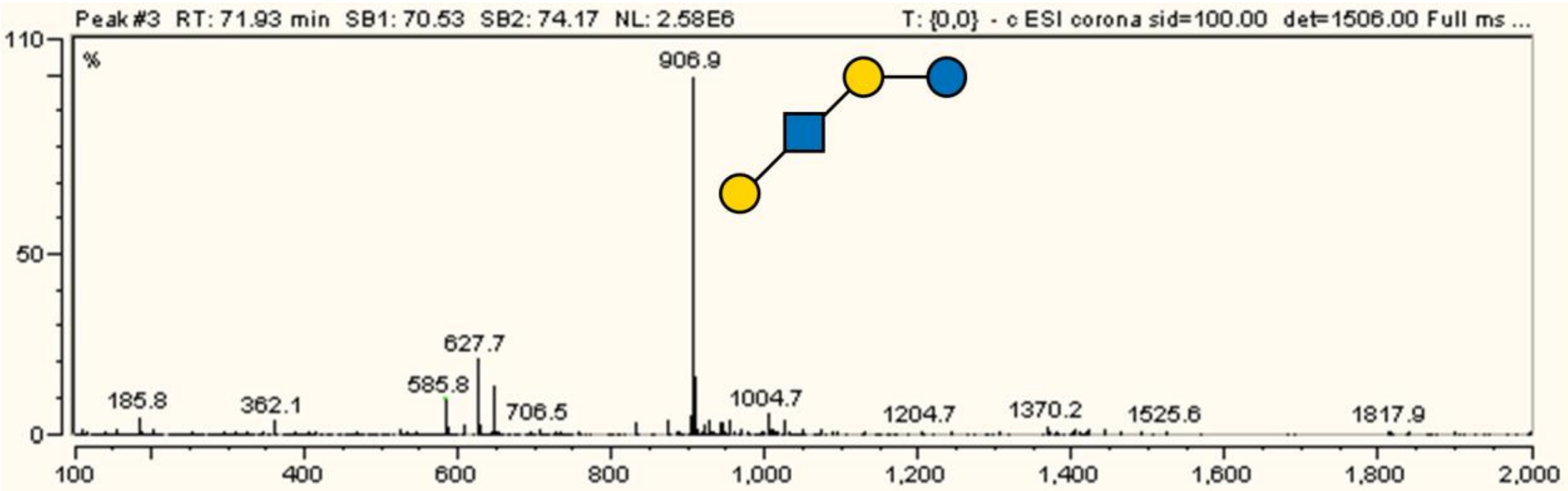

Fig. S48 Mass spectrum (ESI-) of LNT-*t*Boc. [M-H]⁻, m/z 906.9

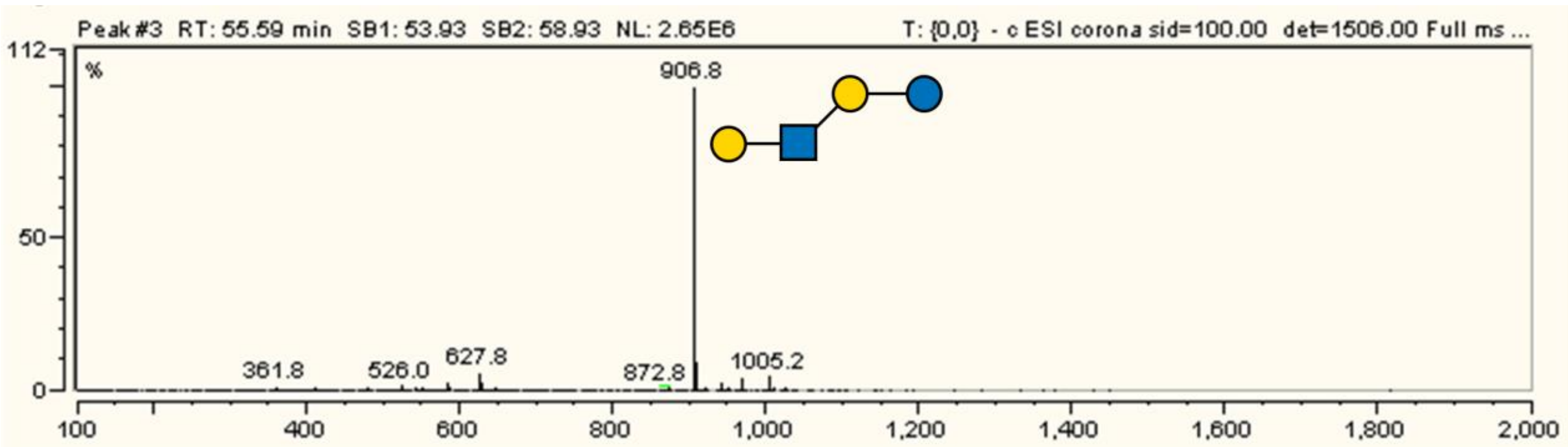

Fig. S49 Mass spectrum (ESI-) of LNnT-*t*Boc. [M-H]⁻, m/z 906.8

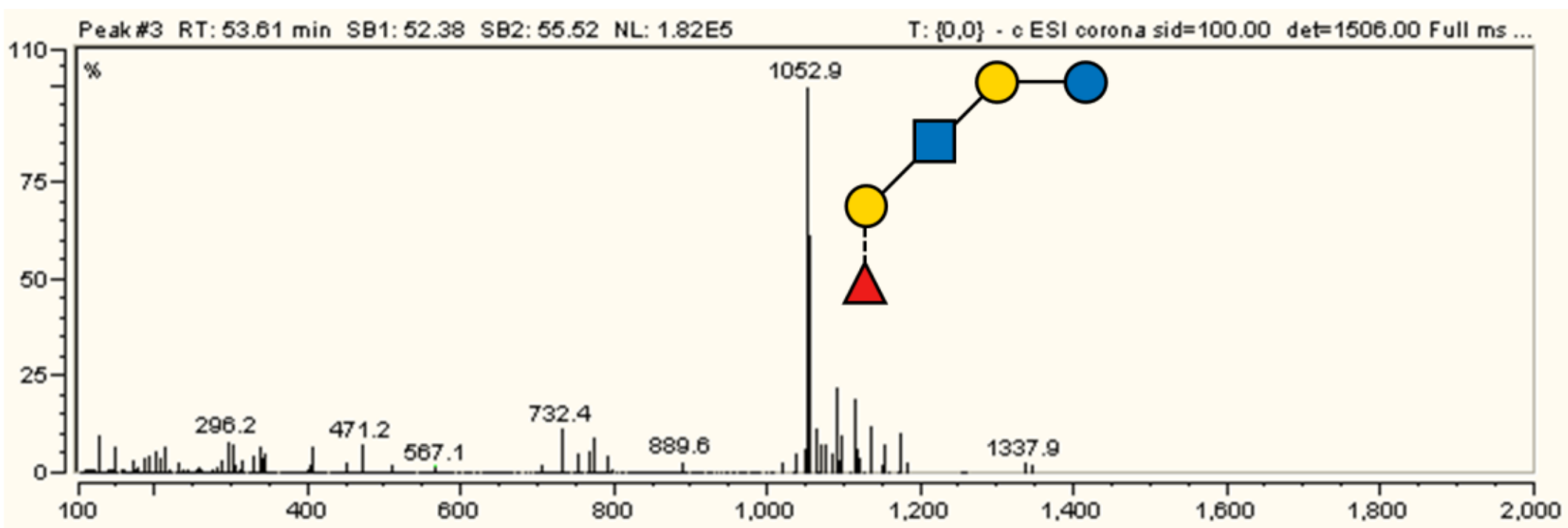

Fig. S50 Mass spectrum (ESI-) of LNFP I-*t*Boc. [M-H]⁻, m/z 1052.9

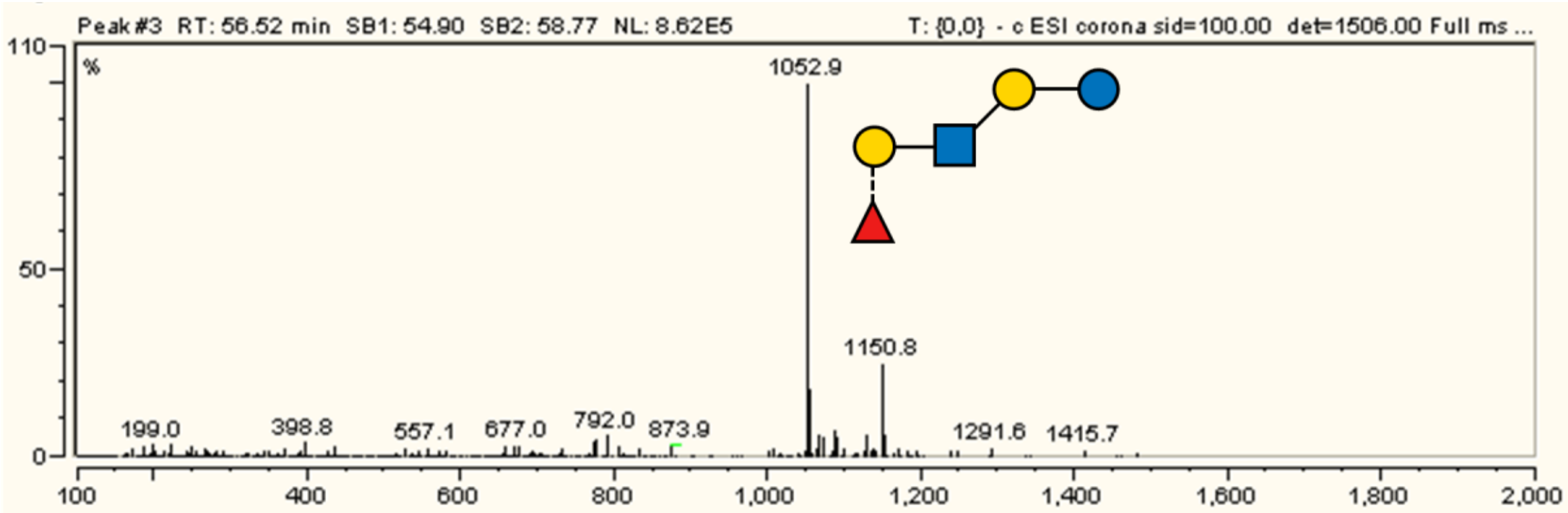

Fig. S51 Mass spectrum (ESI-) of LNnFP I-*t*Boc. [M-H]-, m/z 1052.9

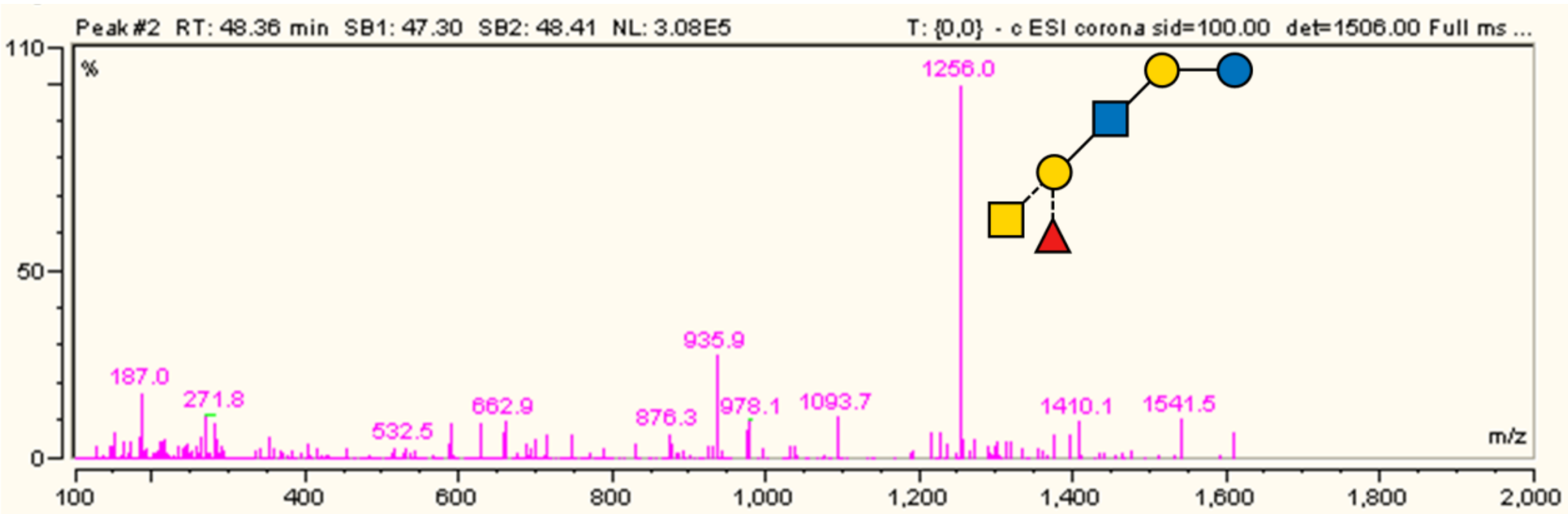

Fig. S52 Mass spectrum (ESI-) of Blood group A antigen hexaose type I-*t*Boc. [M-H]-, m/z 1256.0

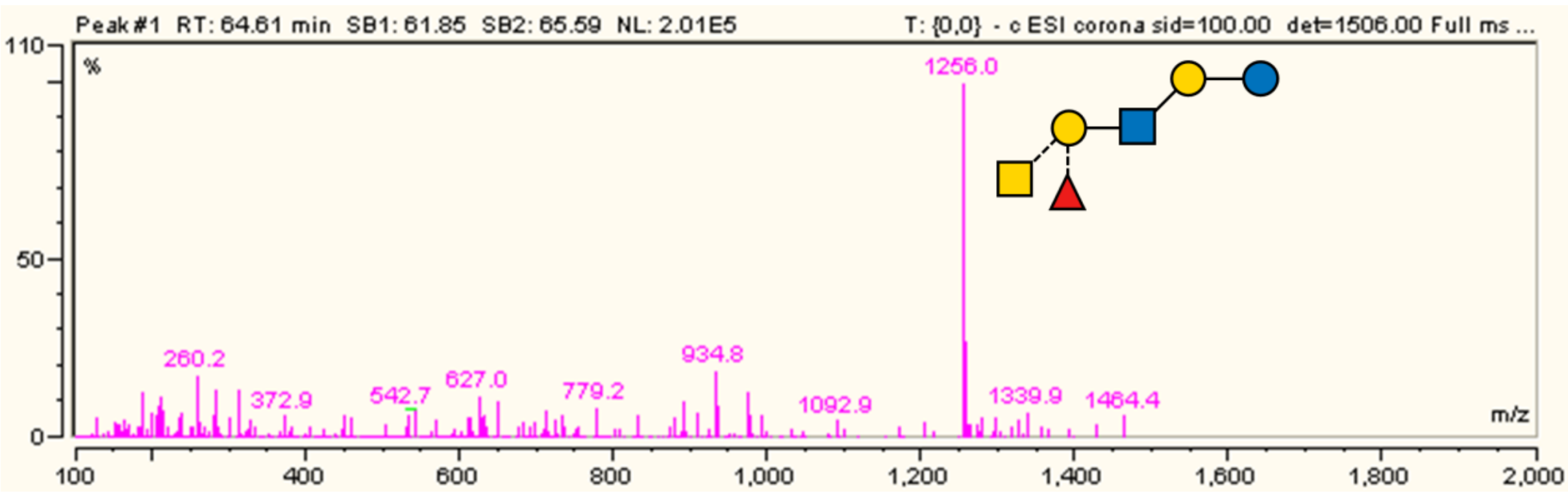

Fig. S53 Mass spectrum (ESI-) of Blood group A antigen hexaose type II-*t*Boc. [M-H]-, m/z 1256.0

6. References

Blackler, R.J., Gagnon, S.M., Polakowski, R., Rose, N.L., Zheng, R.B., Letts, J.A., Johal, A.R., Schuman, B., Borisova, S.N., Palcic, M.M., Evans, S.V., 2017. Glycosyltransfer in mutants of putative catalytic residue Glu303 of the human ABO(H) A and B blood group glycosyltransferases GTA and GTB proceeds through a labile active site. Glycobiology 27, 370-380. https://doi.org/10.1093/glycob/cww117.

Fischöder, T., Wahl, C., Zerhusen, C., Elling, L., 2019. Repetitive Batch Mode Facilitates Enzymatic Synthesis of the Nucleotide Sugars UDP-Gal, UDP-GlcNAc, and UDP-GalNAc on a Multi-Gram Scale. Biotechnol J 14. https://doi.org/10.1002/biot.201800386.

Frohnmeyer, H., Rueben, S., Elling, L., 2022. Gram-Scale Production of GDP-beta-l-fucose with Multi-Enzyme Cascades in a Repetitive-Batch Mode. Chemcatchem 14. https://doi.org/10.1002/cctc.202200443.

Hennig, R., Rapp, E., Kottler, R., Cajic, S., Borowiak, M., Reichl, U., 2015. N-Glycosylation Fingerprinting of Viral Glycoproteins by xCGE-LIF. Methods Mol Biol 1331, 123-143. https://doi.org/10.1007/978-1-4939-2874-3_8.

Liu, W., Tang, S., Peng, J., Pan, L., Wang, J., Cheng, H., Chen, Z., Wang, Y., Zhou, H., 2022. Enhancing heterologous expression of a key enzyme for the biosynthesis of 2'-fucosyllactose. J Sci Food Agric. https://doi.org/10.1002/jsfa.11868.

Naruchi, K., Hamamoto, T., Kurogochi, M., Hinou, H., Shimizu, H., Matsushita, T., Fujitani, N., Kondo, H., Nishimura, S., 2006. Construction and structural characterization of versatile lactosaminoglycan-related compound library for the synthesis of complex glycopeptides and glycosphingolipids. J Org Chem 71, 9609-9621. https://doi.org/10.1021/jo0617161.

Sauerzapfe, B., Krenek, K., Schmiedel, J., Wakarchuk, W.W., Pelantova, H., Kren, V., Elling, L., 2009. Chemo-enzymatic synthesis of poly-N-acetyllactosamine (poly-LacNAc) structures and their characterization for CGL2-galectin-mediated binding of ECM glycoproteins to biomaterial surfaces. Glycoconj J 26, 141-159. https://doi.org/10.1007/s10719-008-9172-2.

Sauerzapfe, B., Namdjou, D.J., Schumacher, T., Linden, N., Krenek, K., Kren, V., Elling, L., 2008. Characterization of recombinant fusion constructs of human beta 1,4-galactosyltransferase 1 and the lipase pre-propeptide from Staphylococcus hyicus. J Mol Catal B-Enzym 50, 128-140. https://doi.org/10.1016/j.molcatb.2007.09.009.

Seto, N.O.L., Palcic, M.M., Compston, C.A., Li, H., Bundle, D.R., Narang, S.A., 1997. Sequential interchange of four amino acids from blood group B to blood group A glycosyltransferase boosts catalytic activity and progressively modifies substrate recognition in human recombinant enzymes. J Biol Chem 272, 14133-14138. https://doi.org/10.1074/jbc.272.22.14133.

Slamova, K., Cerveny, J., Meszaros, Z., Friede, T., Vrbata, D., Kren, V., Bojarova, P., 2023. Oligosaccharide Ligands of Galectin-4 and Its Subunits: Multivalency Scores Highly. Molecules 28. https://doi.org/10.3390/molecules28104039.

Sonnad, J.R., Goudar, C.T., 2004. Solution of the haldane equation for substrate inhibition enzyme kinetics using the decomposition method. Math Comput Model 40, 573-582. https://doi.org/10.1016/j.mcm.2003.10.051.

Stein, D.B., Lin, Y.N., Lin, C.H., 2008. Characterization of α1,2-Fucosyltransferase for Enzymatic Synthesis of Tumor-Associated Antigens. Adv Synth Catal 350, 2313-2321. https://doi.org/10.1002/adsc.200800435.

Wahl, C., Hirtz, D., Elling, L., 2016. Multiplexed capillary electrophoresis as analytical tool for fast optimization of multi-enzyme cascade reactions synthesis of nucleotide sugars. Biotechnol J 11, 1298-1308. https://doi.org/10.1002/biot.201600265.

Yi, W., Liu, X., Li, Y., Li, J., Xia, C., Zhou, G., Zhang, W., Zhao, W., Chen, X., Wang, P.G., 2009. Remodeling bacterial polysaccharides by metabolic pathway engineering. Proc Natl Acad Sci U S A 106, 4207-4212. https://doi.org/10.1073/pnas.0812432106.
